# Supplementary material for: Cohort profile: The Scottish SHARE Mental Health (SHARE-MH) cohort – linkable survey, genetic and routinely collected data for mental health research
Source: BMJ Open. 2024 Jan 12;14(1):e078246. doi: 10.1136/bmjopen-2023-078246 (PMC10806588; doi:10.1136/bmjopen-2023-078246)
Supplement: Supplementary data [file bmjopen-2023-078246supp001.pdf]

Mental Health Data Science Scotland

Block 1: Introduction

Mental health questionnaire for SHARE participants

Questions about your experiences in relation to mental health

Thank you for taking the time to assist us. We are hoping to understand the causes of mental health conditions so that we can try to improve the lives of people who are affected by mental illness. We are interested in your responses whether or not you have ever experienced mental illness yourself.

We are interested in knowing more about the stresses and strains of life and your mental health. Some of the questions are sensitive and may be difficult to answer. Please try to answer as many questions as you can and give the most accurate answers possible. However, if you **cannot** answer a question, you can choose the **"don't know"** or **"prefer not to answer"** option where available. In some instances, it is possible to skip sections and move on.

All responses to questions asked in this questionnaire will be kept strictly confidential, they will only be shared with researchers in a way that means you cannot be identified.

To make it easier for you to participate, you can start the questionnaire and if you leave part way, it will remember where you were when you return to it later. You can see your progress through the survey in the blue bar at the top of the page.

Information on where to find help for the issues covered in this questionnaire is given below and at the end of the survey.

If you feel you need any further help with the issues in this questionnaire, we recommend talking

Iveson MH, *et al. BMJ Open* 2024; 14:e078246. doi: 10.1136/bmjopen-2023-078246

it through with someone you trust, including your GP. For more information and support, visit The Scottish Association for Mental Health (SAMH).

If you have been upset by remembering sexual violence or any other kind of abuse/crime, please contact support groups for aid or information, such as Scottish Women's Aid (for urgent assistance, give them a call on 0800 027 1234), ManKind (01823 334244 10am-4pm), LGBTdomesticabuse or online Victim Support Scotland.

If you are in distress and need urgent help or advice, please contact someone as soon as possible, such as the **Samaritans on 116 123**

If you would like to take part in the research, please read and select **all** of the statements below.

- ☐ I understand that continuing with this questionnaire is **voluntary** and that I can stop at any point without giving a reason
- ☐ I confirm that I am 16 or older
- ☐ I understand that researchers will combine my answers with an anonymised version of my health records for use in research
- ☐ I consent to take part in this study

Block 2: Basics

What age are you now?

- ○ ______ Current age (years)

Which **gender** do you **identify** with?

- ○ Male
- ○ Female
- ○ Non-binary
- ○ Another gender identity
- ○ Don't know
- ○ Prefer not to answer

Thank you for your interest in taking part in this study. We appreciate your support and willingness to take part in research. You have received this message because you have said that you are **under 16** and we are currently only recruiting participants who are **16 or over**. We would be very interested in hearing from you once you are 16.

Block 3: Demographics/lifestyle

What is your **current marital/relationship status**?

- Single
- Relationship (not living together)
- Relationship (living together)
- Married
- Civil partnership
- Separated
- Divorced
- Widowed
- Other
- Prefer not to say

What is your **sexual orientation?**

- Heterosexual/straight
- Homosexual/gay/lesbian
- Bisexual/bi
- Another sexual orientation
- Don't know
- Prefer not to answer

Do you consider yourself to have a disability?

- Yes, a physical disability
- Yes, a mental health disability
- Yes, both a physical and mental health disability
- No, I don't have a disability
- Don't know
- Prefer not to answer

How old were you when you left High School?

- ○ [ ] Age (years)
- ○ I am still in High School

How old were you when you left full-time education?

- ○ [ ] Age (years)
- ○ I am still in full-time education

Which of these qualifications do you have?

*Please select **ALL** that apply*

- ☐ O Grade, Standard Grade, Access 3 Cluster, Intermediate 1 or 3, GCSE, CSE, Senior Certificate or equivalent
- ☐ SCE Higher Grade, Higher, Advanced Higher, CSYS, A Level, AS Level, Advanced Senior Certificate or equivalent
- ☐ GSVQ Foundation or Intermediate, SVQ level 1 or 2, SCOTVEC Module, City and Guilds Craft or equivalent
- ☐ GSVQ Advanced, SVQ level 3, ONC, OND, SCOTVEC National Diploma, City and Guilds Advanced Craft or equivalent
- ☐ HNC, HND, SVQ level 4 or equivalent
- ☐ Degree, Postgraduate qualifications, Masters, PhD, SVQ level 5 or equivalent
- ☐ Professional qualifications (for example, teaching, nursing, accountancy)
- ☐ Other school qualifications not already mentioned (including foreign qualifications)
- ☐ Other post-school but pre-Higher Education qualifications not already mentioned (including foreign qualifications)
- ☐ Other Higher Education qualifications not already mentioned (including foreign qualifications)
- ☐ No qualifications

What is your **current employment status**?
You can only choose one option so please choose the one that best describes your employment status.

- ○ In paid employment or self-employed - Full Time

- In paid employment or self-employed - Part Time
- Retired (may include volunteering and/or caring for home and/or family)
- Looking after home and/or family
- Unable to work because of sickness or disability
- Unemployed
- Doing unpaid or voluntary work
- Full or part-time student
- None of the above
- Prefer not to answer

How would you describe your ethnic origin?

- White
- Mixed
- Asian or Asian British
- Black or Black British
- Other ethnic group
- Don't know
- Prefer not to answer

The next question is about your **height**. Please select your preferred units of measure.

- Feet/inches
- Centimetres

What is your **current** height?
(If you are unsure, please give your best estimate)

- [ ] ____ Height (cm)
- [ ] ____ Height (feet)
- [ ] ____ Height (inches)

The next question is about your **weight**. Please select your preferred units of measure.

- Stones/pounds
- Kilograms

What is your **current** weight?
(If you are unsure, please give your best estimate)

- ☐ ______ Weight (kg)
- ☐ ______ Weight (stones)
- ☐ ______ Weight (pounds)

Has a health professional (doctor, nurse, physiotherapist or similar) ever told you that you were overweight?
Please do not include times during pregnancy

- ○ Yes
- ○ No
- ○ Don't know
- ○ Prefer not to answer

How many **hours per day** do you **sleep** on average?
Please include night time sleep **and** day time naps.

- ○ ______ Average number of hours per day

Do you have a specific diet?

- ○ Vegetarian
- ○ Vegan
- ○ Pescatarian
- ○ Other
- ○ No
- ○ Prefer not to answer

Block 4: Mental Health History

This section has some questions about your mental health

Iveson MH, *et al. BMJ Open* 2024; 14:e078246. doi: 10.1136/bmjopen-2023-078246

In your life, have you suffered from a **period of mental distress** that prevented you from doing your **usual activities**?

- Yes
- No
- Don't know
- Prefer not to answer

In your life, did you **seek or receive help** from a **professional** (medical doctor, psychologist, social worker, counsellor, nurse, clergy or other helping professional) for mental distress, psychological problems or unusual experiences?

- Yes
- No
- Don't know
- Prefer not to answer

Have you ever hurt yourself on purpose in any way (e.g. by taking an overdose of pills, or by cutting yourself)?

- Yes
- No
- Don't know
- Prefer not to answer

Have **YOU** ever been **diagnosed** with one or more of the following illnesses or conditions by a **professional**, even if you don't have it currently?
By professional we mean: any doctor, nurse or person with specialist training (such as a psychologist, psychiatrist etc.).

Please include illnesses or conditions even if you did not need treatment for them or if you did not agree with the diagnosis.

*Please select **ALL** that apply*

- ☐ Addiction or substance use disorder (such as alcohol addiction, drug addiction)

Iveson MH, *et al. BMJ Open* 2024; 14:e078246. doi: 10.1136/bmjopen-2023-078246

- [ ] Anxiety (such as generalised anxiety disorder, social anxiety, panic disorder or post-traumatic stress disorder)
- [ ] Autism, Asperger's or autistic spectrum disorder
- [ ] Attention deficit or attention deficit and hyperactivity disorder (ADD/ADHD)
- [ ] Bipolar disorder, mania, hypomania, manic-depression
- [ ] Depression (such as major depressive disorder, seasonal affective disorder, postnatal depression)
- [ ] Eating disorder (such as anorexia, bulimia, binge eating disorder)
- [ ] Obsessive-compulsive disorder (OCD)
- [ ] Personality disorder
- [ ] Phobia (such as social phobia, agoraphobia or another phobia, for example a disabling fear of heights or spiders
- [ ] Schizophrenia or psychosis
- [ ] None of the above
- [ ] Don't know
- [ ] Prefer not to answer

You mentioned that you have been diagnosed with anxiety (such as generalised anxiety disorder, social anxiety, panic disorder or post-traumatic stress disorder). *Please tell us what type(s) of anxiety you have been diagnosed with*

- [ ] Anxiety, nerves or generalised anxiety disorder
- [ ] Panic disorder
- [ ] Post-traumatic stress disorder (PTSD)
- [ ] Social anxiety
- [ ] Don't know
- [ ] Prefer not to answer

You mentioned that you have been diagnosed with depression (such as major depressive disorder, seasonal affective disorder or postnatal depression). *Please tell us what type(s) of depression you have been diagnosed with*

- [ ] Major depressive disorder
- [ ] Postnatal depression (depression during pregnancy for you or your partner or within a year of giving birth)
- [ ] Seasonal Affective Disorder (SAD)
- [ ] Don't know
- [ ] Prefer not to answer

Iveson MH, *et al. BMJ Open* 2024; 14:e078246. doi: 10.1136/bmjopen-2023-078246

You mentioned that you have had postnatal depression. Were you the person who was pregnant?

- Yes, I was pregnant
- No, my partner was pregnant

You mentioned that you have been diagnosed with an eating disorder.
Please tell us which eating disorder(s) you have been diagnosed with

- Anorexia nervosa
- Atypical anorexia nervosa
- Bulimia nervosa
- Psychological over-eating or binge eating
- Don't know
- Prefer not to answer

You mentioned that you have been diagnosed with a personality disorder.
Please tell us which personality disorder(s) you have been diagnosed with

- Borderline personality disorder
- Other personality disorder
- Don't know
- Prefer not to answer

You mentioned that you have been diagnosed with a phobia.
Please tell us which phobia(s) you have been diagnosed with

- Social phobia
- Agoraphobia
- Other phobia (such as a disabling fear of heights or spiders)
- Don't know
- Prefer not to answer

You mentioned that you have been diagnosed with schizophrenia or another type of psychosis.

Please tell us which type of psychosis you have been diagnosed with.

- [ ] Schizophrenia
- [ ] Other type of psychosis
- [ ] Don't know
- [ ] Prefer not to answer

Block 5: Mood

The next section asks a few questions about your **mood** and **feelings.**

Have you ever had a time in your life when you have felt sad, blue or depressed for **two weeks or more** in a row?

- Yes
- No
- Prefer not to answer

Have you ever had a time in your life lasting **two weeks or more** when you lost interest in most things like hobbies, work, or activities that usually give you pleasure?

- Yes
- No
- Prefer not to answer

In the next section, we would like to know more about your **depression or low mood**. If you think you may find this distressing, you can **skip** these questions.

- Continue
- Skip

Please think of the **two-week period** in your life when your feelings of depression or loss of interest were worst

How much of the day did these feelings usually last?

- All day long
- Most of the day
- About half of the day
- Less than half of the day

- Don’t know
- Prefer not to answer

Please think of the **two-week period** in your life when your feelings of depression or loss of interest were worst

Did you feel this way:

- Every day
- Almost every day
- Less often
- Don’t know
- Prefer not to answer

Please think of the **two-week period** in your life when your feelings of depression or loss of interest were worst

Did you feel more **tired** out or **low on energy** than is usual for you?

- Yes
- No
- Don't know
- Prefer not to say

Did you gain or lose weight without trying, or did you stay about the same weight?

- Gained weight
- Lost weight
- Both gained and lost weight during the same episode
- Stayed about the same or was on a diet
- Don't know
- Prefer not to say

Did your weight change by about **10lbs (4kg)** or more?

- Yes
- No
- Don't know
- Prefer not to say

Please think of the **two-week period** in your life when your feelings of depression or loss of interest were worst

Did your **sleep** change?

- Yes
- No
- Don't know
- Prefer not to say

Was that:

Please answer all four questions

	Yes	No
Trouble falling asleep	○	○
Waking too early	○	○
Sleeping too much	○	○
Both sleeping too much and too little during the same episode of depression or loss of interest	○	○

How many **hours per day** did you **sleep on average** during the episode of depression or loss of interest? Please include night time sleep **and** day time naps.

- ____ Hours sleep per day

How many **hours per day** did you used to **sleep** on average when you were **not depressed**?
Please include night time sleep **and** day time naps.

- ____ Hours sleep per day

Please think of the **two-week period** in your life when your feelings of depression or loss of interest were worst

Did you experience a **change** in your appetite?

- Increased appetite
- Decreased appetite

- Both increased and decreased appetite during the same episode of depression or loss interest
- No changes in appetite
- Don't know
- Prefer not to answer

Please think of the **two-week period** in your life when your feelings of depression or loss of interest were worst

Did your **mood brighten** in response to **positive events**?

- Yes
- No
- Don't know
- Prefer not to answer

Please think of the **two-week period** in your life when your feelings of depression or loss of interest were worst

Were you **overly sensitive** to comments from other people ?

- Yes, and this **significantly** impaired your social or work relationships
- Yes, but this did **not significantly** impair your social or work relationships
- No
- Don’t know
- Prefer not to answer

Please think of the **two-week period** in your life when your feelings of depression or loss of interest were worst

Was your mood **worse:**

- In the **morning**
- In the **afternoon**
- At **night**
- My mood did **not** vary
- Don't know
- Prefer not to answer

Please think of the **two-week period** in your life when your feelings of depression or loss of interest were worst

Did you have a lot more **trouble concentrating than usual?**

- Yes
- No
- Don't know
- Prefer not to answer

Please think of the **two-week period** in your life when your feelings of depression or loss of interest were worst

Did you notice a change in your interest in sex?

- Yes
- No
- Don't know
- Prefer not to answer

Please think of the **two-week period** in your life when your feelings of depression or loss of interest were worst

People sometimes **feel down** on themselves, no good, worthless. Did you feel this way?

- Yes
- No
- Don't know
- Prefer not to answer

Please think of the **two-week period** in your life when your feelings of depression or loss of interest were worst

Did you **think** a lot about **death** – either your own, or someone else’s, or death in general?

- Yes
- No
- Don't know
- Prefer not to answer

Did you feel so low you thought a lot about ending your life?

- Yes
- No
- Don't know
- Prefer not to answer

Did you make a suicide plan?

- Yes
- No
- Don't know
- Prefer not to answer

Did you attempt to end your life?

- Yes
- No
- Don't know
- Prefer not to answer

Please think of the **two-week period** in your life when your feelings of depression or loss of interest were worst

Think about your **roles** at the time of this episode, including study/employment, childcare, housework and leisure pursuits. How much did your feelings of depression or loss of interest **interfere** with your life or activities?

- A lot
- Some
- A little
- Not at all
- Don't know
- Prefer not to answer

How many periods of depression or low mood have you had in your life **lasting two or more weeks?**

- One

- Two or three
- More than three
- Don't know
- Prefer not to answer

Please estimate the number of separate episodes of depression or low mood in your life lasting two or more weeks:

- Estimate of number of depressive episodes

Did any of these episodes occur **during pregnancy (for you or your partner)** or **within the first year of giving birth?** Or has it been suggested you had postnatal depression?

(We are interested in your answer regardless of gender)

- Yes
- No
- Don't know
- Prefer not to answer

You mentioned that you have had postnatal depression. Were you the person who was pregnant?"

- Yes, I was pregnant
- No, my partner was pregnant

About how old were you the **first** time you had a **period of two weeks** like this? (Whether or not you received any help for it.)
An approximate age is fine

- Approx. age of **first** depressive episode (years)

About how old were you the **last** time you had a **period of two weeks** like this? (Whether or not you received any help for it.)
An approximate age is fine

- Approx. age of **most recent** depressive episode (years)

Did you **ever** tell a professional about these problems? *(Medical doctor, psychologist, social worker, counselor, nurse, clergy, or other helping professional)*

- ○ Yes
- ○ No
- ○ Don't know
- ○ Prefer not to say

Did you **ever** try or are you **currently** trying any of the following for these problems?
*(Please select **ALL** that apply)*

- ☐ Medications prescribed to you for at least two weeks
- ☐ Unprescribed medication more than once
- ☐ Drugs or alcohol more than once
- ☐ Psychotherapy or other talking therapy more than once
- ☐ None of the above
- ☐ Prefer not to answer

Did you take your medication **as advised**?

- ○ Yes, at least mostly
- ○ No
- ○ Don't know
- ○ Prefer not to answer

Do you think that the prescribed medications helped or are helping you?

- ○ Yes
- ○ No
- ○ Don't know
- ○ Prefer not to answer

You mention that one (or more) medications was helpful or not helpful to you. Some commonly prescribed medications are given below. Please tell us which you found helpful or unhelpful.

The same medications can have many different names. Each that we mention has been given its common name (e.g. Citalopram) and some common brand names (e.g. celexa, cipramil)

*Please choose an option for **ALL** medications*

	Medication **helpful**	Medication **somewhat helpful**	Medication **not helpful**	Not relevant (medication **no** **prescribed**)
Amitriptyline *Also called: Domical, Elavil, Lentizol, Tryptizol*	○	○	○	○
Citalopram *Also called: Celexa, Cipramil*	○	○	○	○
Fluoxetine *Also called: Prozac, Prozep, Olena, Oxactin*	○	○	○	○
Mirtazapine *Also called: Zispin*	○	○	○	○
Sertraline *Also called: Lustral*	○	○	○	○
Venlafaxine *Also called: Efexor*	○	○	○	○
Other medication not mentioned above	○	○	○	○

You mention that you have tried or are trying psychotherapy or another talking therapy more than once. Please tell us which you have tried and whether you found it helpful or unhelpful.

*Please choose an option for **ALL** therapy types*

	Therapy **helpful**	Therapy **somewhat helpful**	Therapy **not helpful**	Not relevant (not tried this therapy)	Don't know	Prefer not to answe
Counselling	○	○	○	○	○	○
Mindfulness	○	○	○	○	○	○

Iveson MH, *et al. BMJ Open* 2024; 14:e078246. doi: 10.1136/bmjopen-2023-078246

	Therapy **helpful**	Therapy **somewhat helpful**	Therapy **not helpful**	Not relevant (not tried this therapy)	Don't know	Prefer not to answer
[illegible]						
Relationship therapy						
Group therapy						
Guided self-help						
Family therapy						
Cognitive behavioural therapy (CBT)						
Workshops						
Online therapy						
Other						

We have been asking you about a two-week period but **altogether** over your life, **how long** have your feelings of depression or loss of interest lasted?

If you are unsure, please estimate

- Less than a month
- Between **one** and **three months**
- **Over** three months, but **less** than six months
- **Over** six months, but **less** than 12 months
- Between **one** and **two years**
- **Over** two years
- Don't know
- Prefer not to answer

Was your depression or loss of interest **worse:**

- In the **Spring**
- In the **Summer**
- In the **Autumn**
- In the **Winter**
- My mood did **not** vary with the time of year
- Don't know
- Prefer not to answer

Block 6: Bipolar illness and psychosis

Now we would like to know about some different symptoms in your **lifetime**. Please answer each question to the best of your ability.

Has there **ever** been a **period of time** when you were not your usual self and...

(Please respond to each item)

	Yes	No
...you felt so **good** or so **hyper** that other people thought you were not your normal self or you were so hyper that you got into **trouble**?	○	○
...you were so **irritable** that you shouted at people or started fights or arguments?	○	○
...you felt **much more self-confident** than usual?	○	○
...you got much **less** sleep than usual and found you didn't really **miss** it?	○	○
…you were much more talkative or spoke **much faster** than usual?	○	○
...thoughts raced through your head or you couldn't slow your mind down?	○	○
...you were so **easily distracted** by things around you that you had trouble concentrating or staying on track?	○	○
...you had **much more energy** than usual?	○	○
...you were **much more active** or did many more things than usual?	○	○
...you were **much more social** or outgoing than usual, for example, you telephoned friends in the middle of the night?	○	○
...you were **much more interested** in sex than usual?	○	○
...you did things that were unusual for you or that other people might have thought were **excessive, foolish**, or **risky**?	○	○
...spending money got you or your family into trouble?	○	○

You ticked **'yes'** to more than one of the previous symptoms – have **several** of these ever happened during the **same period of time**?

- ○ Yes
- ○ No

You indicated that you experienced the following. Please select **all** that occurred during the **same period of time**

- ☐ Prefer not to answer
- ☐ Don't know

What is the **longest time** that these "high" or "irritable" periods have lasted?

- ○ Less than 24 hours

Iveson MH, *et al. BMJ Open* 2024; 14:e078246. doi: 10.1136/bmjopen-2023-078246

- At least a day, but less than a week
- A week or more
- Don't know
- Prefer not to answer

How much of a **problem** did any of these cause you - like being unable to work; having family, money or legal troubles; getting into arguments or fights?

- No problem
- Minor problem
- Moderate problem
- Serious problem
- Prefer not to answer

Block 7: Anxiety or nerves

This section asks some questions about **anxiety** or **nerves**.

People differ a lot in how much they worry about things. Did you ever have a time when you worried a lot **more than most people** would in your situation?

- Yes
- No
- Don't know
- Prefer not to answer

Have you ever had a period lasting **one month or longer** when most of the time you felt worried, tense, or anxious?

- Yes
- No
- Don't know
- Prefer not to answer

What is the **longest period of time** that this kind of worrying has ever continued? *(If you are not sure of the exact amount of time, please give an estimate)*

☐ ______ Years
☐ ______ Months

Please think of the period in your life when you have felt **worried, tense, anxious,** or **more worried** than most people would in your situation. This could be in the past, or it could be continuing now.
The following questions refer to this period of time.

During that period, was your worry **stronger** than in other people?

- Yes
- No
- Don't know
- Prefer not to answer

Please think of the period in your life when you have felt **worried, tense, anxious,** or **more worried** than most people would in your situation. This could be in the past, or it could be continuing now.
The following questions refer to this period of time.

Did you worry **most days**?

- Yes
- No
- Don't know
- Prefer not to answer

Please think of the period in your life when you have felt **worried, tense, anxious,** or **more worried** than most people would in your situation. This could be in the past, or it could be continuing now.
The following questions refer to this period of time.

Did you usually worry about **one particular thing**, such as your job security or the failing health of a loved one, or **more than one thing**?

Iveson MH, *et al. BMJ Open* 2024; 14:e078246. doi: 10.1136/bmjopen-2023-078246

- One thing
- More than one thing
- Don't know
- Prefer not to say

Please think of the period in your life when you have felt **worried, tense, anxious,** or **more worried** than most people would in your situation. This could be in the past, or it could be continuing now.
The following questions refer to this period of time.

Did you find it **difficult to stop** worrying?

- Yes
- No
- Don't know
- Prefer not to answer

Please think of the period in your life when you have felt **worried, tense, anxious,** or **more worried** than most people would in your situation. This could be in the past, or it could be continuing now.
The following questions refer to this period of time.

Did you ever have different worries on your mind **at the same time**?

- Yes
- No
- Don't know
- Prefer not to answer

Please think of the period in your life when you have felt **worried, tense, anxious,** or **more worried** than most people would in your situation. This could be in the past, or it could be continuing now.
The following questions refer to this period of time.

How often was your worry so strong that you **couldn't** put it out of your mind no matter how hard you tried?

Iveson MH, *et al. BMJ Open* 2024; 14:e078246. doi: 10.1136/bmjopen-2023-078246

- Often
- Sometimes
- Rarely
- Never
- Don't know
- Prefer not to answer

Please think of the period in your life when you have felt **worried, tense, anxious,** or **more worried** than most people would in your situation. This could be in the past, or it could be continuing now.
The following questions refer to this period of time.

How often did you find it **difficult to control** your worry?

- Often
- Sometimes
- Rarely
- Never
- Don't know
- Prefer not to answer

When you were **worried or anxious**, were you also:

	Yes	No	Don't know
Restless?	○	○	○
Keyed up or on edge?	○	○	○
Easily tired?	○	○	○
Having difficulty keeping your mind on what you were doing?	○	○	○
More irritable than usual?	○	○	○
Having tense, sore, or aching muscles?	○	○	○
Often having trouble falling or staying asleep?	○	○	○

Did you **ever** tell a **professional** about these problems? *(medical doctor, psychologist, social worker, counsellor, nurse, clergy, or other helping professional)*

- Yes

- No
- Don't know
- Prefer not to answer

Regarding times in your life when you have felt **worried, tense or anxious:** Did you **ever** use the following for the worry or the problems it caused?
Please include any treatments that you have already told us about under 'depression' if they were also for anxiety.
(Please select all that apply)

- Medications prescribed to you for at least two weeks
- Specific anti-anxiety medication prescribed to you for at least one week
- Unprescribed medication more than once
- Drugs or alcohol more than once
- Psychotherapy or other talking therapy more than once
- None of the above
- Prefer not to answer

Did you take your medication **as advised**?

- Yes, at least mostly
- No
- Don't know
- Prefer not to answer

You mention that you have tried or are trying psychotherapy or another talking therapy more than once. Please tell us which you have tried and whether you found it helpful or unhelpful.

	Therapy **helpful**	Therapy **not helpful**	Not relevant (not tried this therapy)	Don't know	Prefer not to answer
Counselling	○	○	○	○	○
Mindfullness	○	○	○	○	○
Relationship therapy	○	○	○	○	○
Group therapy	○	○	○	○	○

	Therapy **helpful**	Therapy **not helpful**	Not relevant (not tried this therapy)	Don't know	Prefer not to answer
Guided self-help	○	○	○	○	○
Family therapy	○	○	○	○	○
Cognitive behavioural therapy (CBT)	○	○	○	○	○
Workshops	○	○	○	○	○
Online therapy	○	○	○	○	○
Other	○	○	○	○	○

Regarding times in your life when you have felt **worried, tense or anxious:**

Think about your **roles** at the time of this episode, including study/employment, childcare and housework, leisure pursuits. How much did these problems **interfere** with your life or activities?

- ○ A lot
- ○ Some
- ○ A little
- ○ Not at all
- ○ Prefer not to answer

Block 8: Alcohol

In this section, we would like to ask you about alcohol, as we think it may influence mental health.

Your answers will remain confidential so please be honest.

Please state how much you agree with the following statement.

A little bit of alcohol can be good for you

- ○ Very much
- ○ Quite a bit
- ○ Somewhat
- ○ A little bit

- Not at all
- Don't know
- Prefer not to answer

The next questions are about how frequently you drink alcoholic beverages.

Have you ever had an alcoholic drink?

- Yes, I currently drink alcohol
- Yes, but I stopped drinking within the last 12 months
- Yes, but I stopped drinking more than 12 months ago
- No, I have never had an alcoholic drink

Please think about your use of alcoholic drinks in the **last year**.

How often do you have a drink containing **alcohol?**

- Never
- Monthly or less
- 2 to 4 times a month
- 2 to 3 times a week
- 4 or more times a week

Please think about your use of alcohol drinks in the **last year.**

How many units of alcohol do you normally drink in a week?

Alcoholic drink	Approx. units
1 pint of ordinary beer, cider or lager	2
1 pint of extra strength beer /lager	3
1 can of cider	2

1 litre of cider	5
1 glass of wine (175ml)	2
1 bottle of wine	10
1 bottle of fortified wine (e.g. buckfast or port) (75cl)	11
1 litre of fortified wine (e.g. buckfast or port)	15
1 small glass of sherry	1
1 bottle of sherry (100cl)	15
1 pub measure of spirits (25ml)	1
1 bottle of spirits (75cl)	30
1 bottle of alcopops	2

- [ ] ______ Approximate number of units (per week)

Please think about your use of alcoholic drinks in the **last year**.

How many drinks containing alcohol do you have on a typical day when you are drinking?

- 1 or 2
- 3 or 4
- 5 or 6
- 7, 8 or 9
- 10 or more

Please think about your use of alcoholic drinks in the **last year**.

How often do you have six or more drinks on one occasion?

- Never

- Less than monthly
- Monthly
- Weekly
- Daily or almost daily

How often during the **last year** have you found that you were **not able to stop** drinking once you had started?

- Never
- Less than monthly
- Monthly
- Weekly
- Daily or almost daily

How often during the **last year** have you failed to do what was normally expected from you because of drinking?

- Never
- Less than monthly
- Monthly
- Weekly
- Daily or almost daily

How often during the **last year** have you **needed** a first drink in the **morning** to get yourself going after a heavy drinking session?

- Never
- Less than monthly
- Monthly
- Weekly
- Daily or almost daily

How often during the **last year** have you had a feeling of **guilt or remorse** after drinking?

- Never
- Less than monthly
- Monthly

- Weekly
- Daily or almost daily

How often during the **last year** have you been **unable** to remember what happened the night before because you had been drinking?

- Never
- Less than monthly
- Monthly
- Weekly
- Daily or almost daily

We will now ask you some questions about drinking during your **lifetime**.

Have **you or someone else** been injured as a result of your drinking?

- No
- Yes, but not in the last year
- Yes, during the last year

Has a **relative or friend or a doctor** or another health worker been **concerned** about your drinking or suggested you cut down?

- No
- Yes, but not in the last year
- Yes, during the last year

About how old were you first started drinking alcohol, not counting small tastes or sips?

- [ ] Approximate age (years)

Do you think you have ever been a heavy drinker?

- Yes, now
- Yes, now **and** in the past
- Yes, in the past **but not now**

- No
- Don't know
- Prefer not to answer

Think about the period in your life when you drank the most. How old were you when that period began?

- [ ] Approximate age (years)

How long did that period last?

- [ ] Approximate length of time (years)

During that period when you drank the most, about how often did you drink?

- Monthly or less
- 2 to 4 times a month
- 2 to 3 times a week
- 4 or more times a week
- Don't know
- Prefer not to answer

During that period when you drank the most, how many **units** of alcohol did you drink on a **typical day** when you are drinking?

**Alcoholic drink**	**Approx. units**
1 pint of ordinary beer, cider or lager	2
1 pint of extra strength beer /lager	3
1 can of cider	2
1 litre of cider	5
1 glass of wine (175ml)	2
1 bottle of wine (75cl)	10
1 bottle of fortified wine (e.g. buckfast or port) (75cl)	11
1 litre of fortified wine (e.g. buckfast or port)	15
1 small glass of sherry	1

Iveson MH, *et al. BMJ Open* 2024; 14:e078246. doi: 10.1136/bmjopen-2023-078246

Alcoholic drink	Approx. units
1 bottle of sherry (100cl)	15
1 pub measure of spirits (25ml)	1
1 bottle of spirits (75cl)	30
1 bottle of alcopops	2

- [ ] Approximate number of units (per day)

How much alcohol do you have to drink before you start to feel its effects?

(If you are not sure, your best guess is fine)

- More than other people (drink doesn't seem to affect me as much as other people)
- The same as other people
- Less than other people (I am easily affected by alcohol)
- Don't know
- Prefer not to answer

How bad are your hangovers from drinking alcohol?

- Very bad
- Quite bad
- Not bad at all
- I don't drink enough to get a hangover
- Don't know
- Prefer not to answer

We will now ask you some questions about your reasons for drinking alcohol

Please state how often the following statement is true.

I feel more comfortable around others when I drink

(please think about now and in the past)

- Always or almost always

Iveson MH, *et al. BMJ Open* 2024; 14:e078246. doi: 10.1136/bmjopen-2023-078246

- Often
- Sometimes
- Rarely
- Never
- Don't know
- Prefer not to answer

Please state how often the following statement is true.

I sleep better after I drink

(please think about now and in the past)

- Always or almost always
- Often
- Sometimes
- Rarely
- Never
- Don't know
- Prefer not to answer

Please state how often the following statement is true.

I drink because I am nervous or anxious

(please think about now and in the past)

- Always or almost always
- Often
- Sometimes
- Rarely
- Never
- Don't know
- Prefer not to answer

Please state how often the following statement is true.

I drink because I am depressed

(please think about now and in the past)

- Always or almost always
- Often
- Sometimes
- Rarely
- Never
- Don't know
- Prefer not to answer

Please state how often the following statement is true.

I drink because I am in physical pain

(please think about now and in the past)

- Always or almost always
- Often
- Sometimes
- Rarely
- Never
- Don't know
- Prefer not to answer

Please state how often the following statement is true.

I drink because I am angry, annoyed or bad-tempered

(please think about now and in the past)

- Always or almost always
- Often
- Sometimes
- Rarely

- Never
- Don't know
- Prefer not to answer

Please state how often the following statement is true.

I drink to escape my memories or thoughts

(please think about now and in the past)

- Always or almost always
- Often
- Sometimes
- Rarely
- Never
- Don't know
- Prefer not to answer

Please state how often the following statement is true.

I drink because I feel tense or stressed

(please think about now and in the past)

- Always or almost always
- Often
- Sometimes
- Rarely
- Never
- Don't know
- Prefer not to answer

Block 9: Smoking

In this section, we will ask you about smoking

Iveson MH, *et al. BMJ Open* 2024; 14:e078246. doi: 10.1136/bmjopen-2023-078246

Please select the box that best describes you

- I smoke now
- I used to smoke
- I have never smoked
- Prefer not to answer

How many years has it been since you stopped smoking?
(An approximate answer is fine)

- [ ] Years since stopped smoking

Approximately how many cigarettes do you or did you smoke each day?

- [ ] Number of cigarettes per day

How many years have you / did you smoke?

- [ ] Years smoking

How many hours per week are you **exposed** to other people's tobacco smoke (*passive smoking*)?

- [ ] Hours per week

Have you ever tried an e-cigarette or vaping device?

- Yes
- No
- Don't know
- Prefer not to answer

How often do you currently use an e-cigarette or vaping device?

- Daily or almost daily

- Less than daily, but at least once a week
- Less than weekly, but at least once a month
- Less than monthly
- Not at all
- Don't know
- Prefer not to answer

Have you ever used and e-cigarette or vaping device daily for a month or more?

- Yes
- No
- Don't know
- Prefer not to answer

Block 10: Illegal substances

In this section, we would like to ask you about illegal substances, as we think they may influence mental health.

Your answers will remain confidential so please be honest.

Have you **ever** taken any **drugs** other than those prescribed for you for medical reasons?
For example ***cannabis, cocaine, speed, amphetamines, ecstasy, LSD, heroin*** *or* ***methadone*** *or substances previously called* ***"legal" highs*** *such as* ***spice****.*

We do **not** mean **prescription drugs** and will ask about these later.

- Yes
- No
- Prefer not to answer

You mention that you have taken drugs in your lifetime. Please tell us how often you took them when you were using them most.

Iveson MH, *et al. BMJ Open* 2024; 14:e078246. doi: 10.1136/bmjopen-2023-078246

*Please choose an option for **ALL** groups of drugs*

	Never	Once or twice only	Monthly or less	2 to 4 times a month	2 to 3 times a week	4 or more times a week	Prefer not to answer
Heroin or methadone	○	○	○	○	○	○	○
Cocaine	○	○	○	○	○	○	○
Cannabis or marijuana	○	○	○	○	○	○	○
Speed, amphetamines or ecstasy	○	○	○	○	○	○	○
Spice or other substance previously called a "legal" high	○	○	○	○	○	○	○
Magic mushrooms or LSD	○	○	○	○	○	○	○
Ketamine	○	○	○	○	○	○	○
Other illegal drug	○	○	○	○	○	○	○

And how long did you take heroin or methadone for?
If you are still taking them, please give the time so far.
If you are unsure an estimate is fine.

- ○ [ ] Years
- ○ Less than one year
- ○ [ ] I am still taking heroin or methadone. Years so far
- ○ Prefer not to answer

And how long did you take cocaine for?
If you are still taking it, please give the time so far.
If you are unsure an estimate is fine.

- ○ [ ] Years
- ○ Less than one year
- ○ [ ] I am still taking cocaine. Years so far
- ○ Prefer not to answer

And how long did you take cannabis or marijuana for?
If you are still taking them, please give the time so far.
If you are unsure an estimate is fine.

Iveson MH, *et al. BMJ Open* 2024; 14:e078246. doi: 10.1136/bmjopen-2023-078246

- ______ Years
- Less than one year
- ______ I am still taking cannabis or marijuana. Years so far
- Prefer not to answer

And how long did you take speed, amphetamines or ecstasy for?
If you are still taking them, please give the time so far.
If you are unsure an estimate is fine.

- ______ Years
- Less than one year
- I am still taking speed, amphetamines or ecstasy. Years so far ______
- Prefer not to answer

And how long did you take magic mushrooms or LSD for?
If you are still taking them, please give the time so far.
If you are unsure an estimate is fine.

- ______ Years
- Less than one year
- ______ I am still taking magic mushrooms or LSD. Years so far
- Prefer not to answer

And how long did you take ketamine for?
If you are still taking them, please give the time so far.
If you are unsure an estimate is fine.

- ______ Years
- Less than one year
- ______ I am still taking ketamine. Years so far
- Prefer not to answer

Iveson MH, *et al. BMJ Open* 2024; 14:e078246. doi: 10.1136/bmjopen-2023-078246

And how long did you take spice or other substance previously called a "legal" high for?

If you are still taking them, please give the time so far.
If you are unsure an estimate is fine.

- [ ] Years
- Less than one year
- I am still taking spice or other substance previously called a "legal" high. Years so far [ ]
- Prefer not to answer

Have you **ever** taken **prescription drugs** that were **not prescribed for you?**

For example: ***valium, diazepam, temazepam, other 'benzos'*** *or* ***opioids*** *such as* ***fentanyl, tramadol, buprenorphine, oxycodone****.*

- Yes
- No
- Prefer not to answer

You mention that you have taken prescription drugs (not prescribed for you) in your lifetime. Please tell us how often you took them when you were using them most.

*Please choose an option for **ALL** groups of drugs*

	Never	Once or twice only	Monthly or less	2 to 4 times a month	2 to 3 times a week	4 or more times a week	Prefer not to answer
Valium, diazepam, temazepam or other *"benzo"*	O	O	O	O	O	O	O
Fentanyl, tramadol, buprenorphine, oxycodone or other opiod (excluding heroin and methdaone)	O	O	O	O	O	O	O
Other prescription drug that was not prescribed for you	O	O	O	O	O	O	O

And how long did you take valium, diazepam, temazepam or another "benzo" for?

If you are still taking them, please give the time so far.
If you are unsure an estimate is fine.

- ○ ______ Years
- ○ Less than one year
- ○ I am still taking valium, diazepam, temazepam or another "benzo". Years so far ______
- ○ Prefer not to answer

And how long did you take an opioid such as fentanyl, tramadol, buprenorphine or oxycodone for?
If you are still taking them, please give the time so far.
If you are unsure an estimate is fine.

- ○ ______ Years
- ○ Less than one year
- ○ I am still taking an opioid such as fentanyl, tramadol, buprenorphine or oxycodone. ______ Years so far
- ○ Prefer not to answer

Block 11: Trauma

This section asks about your childhood and the stresses and strains of adult life. Some people may find these questions distressing. If you would prefer not to answer these questions, please use a **skip** button below.

If you are distressed by remembering past crime/abuse, please visit Victim Support Scotland

- ○ Continue
- ○ Skip childhood questions
- ○ Skip adult questions
- ○ Skip childhood and adult questions

When I was growing up...

	Never true	Rarely true	Sometimes true	Often true	Very often true	Prefer not to answer
I felt loved	○	○	○	○	○	○
People in my family hit me so hard that it left me with bruises or marks	○	○	○	○	○	○
I felt that someone in my family hated me	○	○	○	○	○	○
Someone molested me (sexually)	○	○	○	○	○	○
There was someone to take me to the doctor if I needed it	○	○	○	○	○	○

If you feel distressed from remembering past crime/abuse, please visit Victim Support Scotland. *If you have been upset by remembering domestic or sexual violence, there is information available at* *Scottish Women's Aid* *(for urgent assistance, give them a call on 0800 027 1234),* ManKind (01823 334244 10am-4pm) *or* LGBTdomesticabuse

Since I was sixteen...

	Never	Yes, but not in the last 12 months	Yes, within the last 12 months	Prefer not to answer
I have been in a confiding relationship	○	○	○	○
A partner or ex-partner deliberately hit me or used violence in any other way	○	○	○	○
A partner or ex-partner repeatedly belittled me to the extent that I felt worthless	○	○	○	○
A partner or ex-partner sexually interfered with me, or forced me to have sex against my wishes	○	○	○	○
I have struggled to find the money to pay my rent/ mortgage payment	○	○	○	○

If you feel distressed from remembering past crime/abuse, please visit Victim Support Scotland. *If you have been upset by remembering domestic or sexual violence, there*

Iveson MH, *et al. BMJ Open* 2024; 14:e078246. doi: 10.1136/bmjopen-2023-078246

is information available at Scottish Women's Aid (for urgent assistance, give them a call on 0800 027 1234), ManKind (01823 334244 10am-4pm) *or* LGBTdomesticabuse

You said that since you were sixteen, you have been in a confiding relationship. Please indicate how often this was true.

- Rarely true
- Sometimes true
- Often true
- Very often true
- Prefer not to answer

You said that since you were sixteen, a partner or ex-partner deliberately hit you or used violence in another way. Please indicate how often this was true.

- Rarely true
- Sometimes true
- Often true
- Very often true
- Prefer not to answer

If you feel distressed from remembering past crime/abuse, please visit Victim Support Scotland. *If you have been upset by remembering domestic or sexual violence, there is information available at Scottish Women's Aid (for urgent assistance, give them a call on 0800 027 1234),* ManKind (01823 334244 10am-4pm) *or* LGBTdomesticabuse

You said that since you were sixteen, a partner or ex-partner repeatedly belittled you to the extent that you felt worthless. Please indicate how often this was true.

- Rarely true
- Sometimes true
- Often true
- Very often true
- Prefer not to answer

Iveson MH, *et al. BMJ Open* 2024; 14:e078246. doi: 10.1136/bmjopen-2023-078246

If you feel distressed from remembering past crime/abuse, please visit Victim Support Scotland. *If you have been upset by remembering domestic or sexual violence, there is information available at Scottish Women's Aid (for urgent assistance, give them a call on 0800 027 1234),* ManKind (01823 334244 10am-4pm) *or* LGBTdomesticabuse

You said that since you were sixteen, a partner or ex-partner sexually interfered with you, or forced you to have sex against your wishes. Please indicate how often this was true.

- Rarely true
- Sometimes true
- Often true
- Very often true
- Prefer not to answer

If you feel distressed from remembering past crime/abuse, please visit Victim Support Scotland. *If you have been upset by remembering domestic or sexual violence, there is information available at Scottish Women's Aid (for urgent assistance, give them a call on 0800 027 1234),* ManKind (01823 334244 10am-4pm) *or* LGBTdomesticabuse

You said that since you were sixteen, you have struggled to find the money to pay your rent/mortgage payment. Please indicate how often this was true

- Rarely true
- Sometimes true
- Often true
- Very often true
- Prefer not to answer

In your life, have you...

	Never	Yes, but not in the last 12 months	Yes, within the last 12 months	Prefer not to answer
Been a victim of a sexual assault, whether by a stranger or someone you knew	O	O	O	O

	Never	Yes, but not in the last 12 months	Yes, within the last 12 months	Prefer not to answer
Been attacked, mugged, robbed, or been the victim of a physically violent crime	○	○	○	○
Been in a serious accident that you believed to be life-threatening at the time	○	○	○	○
Witnessed a sudden violent death (e.g. murder, suicide, aftermath of an accident)	○	○	○	○
Been diagnosed with a life-threatening illness	○	○	○	○
Been involved in combat or exposed to a war-zone (either in the military or as a civilian)	○	○	○	○

If you feel distressed from remembering past crime/abuse, please visit Victim Support Scotland. *If you have been upset by remembering domestic or sexual violence, there is information available at Scottish Women's Aid (for urgent assistance, give them a call on 0800 027 1234),* ManKind (01823 334244 10am-4pm) *or* LGBTdomesticabuse

Next, is a list of **problems and complaints** that people sometimes have in response to extremely stressful experiences. Please indicate how much you have been bothered by that problem in the **past month**:

	Not at all	A little bit	Moderately	Quite a bit	Extremely
Repeated, disturbing memories, thoughts, or images of a stressful experience?	○	○	○	○	○
Feeling very upset when something reminded you of a stressful experience?	○	○	○	○	○
Avoiding activities or situations because they reminded you of a stressful situation?	○	○	○	○	○
Feeling distant or cut off from other people?	○	○	○	○	○
Feeling irritable or having angry outbursts?	○	○	○	○	○
Difficulty concentrating?	○	○	○	○	○

Iveson MH, *et al. BMJ Open* 2024; 14:e078246. doi: 10.1136/bmjopen-2023-078246

If you feel distressed from remembering past crime/abuse, please visit Victim Support Scotland. *If you have been upset by remembering domestic or sexual violence, there is information available at* *Scottish Women's Aid* *(for urgent assistance, give them a call on 0800 027 1234),* ManKind (01823 334244 10am-4pm) *or* LGBTdomesticabuse

Block 12: Impact

Questions about your work and social life

People's problems sometimes **affect** their ability to do certain day-to-day tasks in their lives.

To rate your problems look at each section and determine on the scale provided **how much** your problem **impairs/impaired your ability** to carry out the activity.

Please complete this section thinking about problems you have been asked about like **anxiety, depression and sleep problems.**

If you have had multiple issues, please think of the issue that has impacted you **most severely**.

Because of my problem my ability to **work** is/was impaired. If you are retired or choose not to have a job for reasons unrelated to your problem, please tick NA *(not applicable).*

- Not at all
- Slightly
- Moderately
- Markedly
- Very severely, I cannot/could not work
- NA
- Prefer not to answer

Because of my problem my **home management** *(Cleaning, tidying, shopping, cooking, looking after home/children, paying bills)* is/was impaired.

- Not at all
- Slightly
- Moderately
- Markedly
- Very severely
- Prefer not to answer

Iveson MH, *et al. BMJ Open* 2024; 14:e078246. doi: 10.1136/bmjopen-2023-078246

Because of my problem my **social leisure activities** *(with other people eg. parties, bars, clubs, outings, visits, dating, home entertainment)* are impaired.

- Not at all
- Slightly
- Moderately
- Markedly
- Very severely
- Prefer not to answer

Because of my problem my **private leisure activities** *(done alone, such as reading, gardening, collecting, sewing, walking alone)* are impaired.

- Not at all
- Slightly
- Moderately
- Markedly
- Very severely
- Prefer not to answer

Because of my problem **my ability to form and maintain close relationships** with others, including those I live with, is impaired.

- Not at all
- Slightly
- Moderately
- Markedly
- Very severely
- Prefer not to answer

Block 13: Eyseneck

The following 12 questions are about your feelings

Does your mood often go up and down?

- Yes

Iveson MH, *et al. BMJ Open* 2024; 14:e078246. doi: 10.1136/bmjopen-2023-078246

- No

Do you ever feel 'just miserable' for no reason?

- Yes
- No

Are you an irritable person?

- Yes
- No

Are your feelings easily hurt?

- Yes
- No

Do you often feel 'fed-up'?

- Yes
- No

Would you call yourself a nervous person?

- Yes
- No

Are you a worrier?

- Yes
- No

Would you call yourself tense or 'highly strung'?

- Yes

- No

Do you worry too long after an embarrassing experience?

- Yes
- No

Do you suffer from nerves?

- Yes
- No

Do you often feel lonely?

- Yes
- No

Are you often troubled about feelings of guilt?

- Yes
- No

Block 14: Subjective wellbeing

This is the **last** section of the survey.
In this section we would like to know how you **feel** about things **in general.**

In general, how **happy** are you?

- Extremely happy
- Very happy
- Moderately happy
- Moderately unhappy
- Very unhappy
- Extremely unhappy

- Don't know
- Prefer not to answer

In general, how happy are you with your **health?**

- Extremely happy
- Very happy
- Moderately happy
- Moderately unhappy
- Very unhappy
- Extremely unhappy
- Don't know
- Prefer not to answer

To what extent do you feel your life to be **meaningful**?

- An extreme amount
- Very much
- A moderate amount
- A little
- Not at all
- Don't know
- Prefer not to answer

Please submit your survey by pressing the next arrow below

If you feel you need any further help with the issues in this questionnaire, we recommend talking it through with someone you trust, including your GP. For more information and support, visit The Scottish Association for Mental Health (SAMH).

If you have been upset by remembering sexual violence or any other kind of abuse/crime, please contact support groups for aid or information, such as Scottish Women's Aid (for urgent assistance, give them a call on 0800 027 1234), ManKind (01823 334244 10am-4pm), LGBTdomesticabuse or online Victim Support Scotland.

If you are in distress and need urgent help or advice, please contact someone as soon as possible, such as the **Samaritans on 116 123**

Powered by Qualtrics
