# Supplementary material for: Cohort profile: The Scottish SHARE Mental Health (SHARE-MH) cohort – linkable survey, genetic and routinely collected data for mental health research
Source: BMJ Open. 2024 Jan 12;14(1):e078246. doi: 10.1136/bmjopen-2023-078246 (PMC10806588; doi:10.1136/bmjopen-2023-078246)
Supplement: Supplementary data [file bmjopen-2023-078246supp002.pdf]

Contact email and patient information leaflet: Version 03 14/11/19

SHARE Online survey – A mental health survey for Scotland

MHDSS – A mental health survey for Scotland

Dear

You very kindly joined SHARE, the Scottish Health Research Register, as you were interested in hearing about opportunities to help with medical research. We are writing to let you know about a research opportunity that has arisen and that might appeal to you. Please see information from the study team below:

Participation in this research study involves completing **an online survey** about mental health.

You can start the survey by clicking the link below:

https://unique.url.per.participant

You will be able to complete the survey on a home computer, tablet or smart-phone.

Further details about the study are provided below.

Contact email and patient information leaflet: Version 03 14/11/19

MHDSS
Mental Health Data Science Scotland

Participant Information Leaflet

What am I being invited to do?

You are invited to take part in an online survey about mental health. The survey answers will be used for health research and there will be no direct compensation for individuals taking part in the survey.

What will the survey involve?

If you decide to take part, you will be asked a series of questions about yourself and any history of mental ill-health you may have had. We will then ask for some details about things that may have an impact on mental health, such as drinking alcohol, taking illegal substances and difficult experiences in your life. Finally, there will be some questions about your feelings. The questions will not include asking for your email or any other identifying information.

Please be aware that the survey concerns mental health and some questions may be distressing for some participants. **Participation is voluntary** and you can leave the survey at any time by closing your internet browser. We also give participants the option to skip some sections should they wish.

How long will the survey take? Can I come back later?

On average, the whole survey takes approximately **15 minutes to complete**. It may take less time if you have limited experience of mental health problems and will probably take **20-25 minutes** if you have more experience with low moods. We do not think it will take anyone more than **one hour**. If you are not able to complete the survey in one go you can **return to it later** by clicking on the link you have been sent (please note you will need to make sure you are using the same computer/device to access the survey). The link you have been sent to the survey is unique so, if more than one person in your household has been sent this email, please only use the link that was sent to you.

What is the research about?

The aim of the research is to better understand why people develop mental illnesses and how that links with their physical health. We will do that by collecting information from participants and linking this with their health records. The information collected will **not** be added to your health record, it will be stored separately and will be only be used for research purposes. It will be **strictly confidential** and only shared with

researchers in a way that means you cannot be identified. In the future, your responses may also be used by other researchers to select you for further studies of interest but your participation would be completely voluntary.

Who is conducting the research?

This survey is being carried out by researchers at the University of Edinburgh and it is funded by a grant from the Medical Research Council (MRC).

What about data security?

The questionnaire deals with sensitive information so every care is being taken with regard to data security. The software we are using to collect answers is called Qualtrics. The company behind this software works with the NHS, companies and universities and has no access to any of the answers that participants give. All answers will be collected, encrypted and stored electronically on an ISO 27001 certified, secure server. They will then be securely transferred to the University of Edinburgh and from there to an NHS data Safe Haven which also uses ISO 27001 certified servers. The information held by the University of Edinburgh will contain no identifying information. Within the Safe Haven, a separate encrypted file will be used to link the responses with anonymised electronic health records. Researchers must then do all of their work within the Safe Haven and cannot remove information about individuals. Results can only be removed about groups of people. If you would like to know more about how we approach data security and how the National Safe Havens work, please visit our website here https://mhdss.ac.uk/what-about-security-and-privacy

Do I have to take part?

No, you do not have to take part in this survey. We will ask you to consent at the beginning of the questionnaire and, if you agree, this would indicate a willingness for your data to be used by the research team. Should you wish to withdraw your data, you can email <<MHDSS email>> and give us the **email address that was used to contact you**. You do not need a reason and we do not need your name or any details of your responses. If your questionnaire answers have already been used in analyses, they cannot be removed because they will have been anonymised before analysis but we will contact the Safe Haven team and they will delete your answers so that they cannot be used again.

Where can I find more information?

You can find out more about the team behind this study and our wider work via our website Mental Health Data Science Scotland or you can email enquiries to the team working with this questionnaire at <<MHDSS email>>.

If you wish to contact someone who has knowledge of the study, but is not part of the team working on this questionnaire, please contact <<External contact>> by emailing <<External’s email address>> or writing to <<External’s postal address>>.

What should I do if I want to make a complaint?

Any concerns should be directed in the first instance to the team at Edinburgh University using <<MHDSS email>>. If you have concerns which you believe have not been addressed then complaints can be made to the Head of the Division of Psychiatry at the University of Edinburgh, <<HoD details>>. Email: <<HoD email address>> or write to <<HoD postal address>>.
