# Supplementary material for: Cohort profile: The Scottish SHARE Mental Health (SHARE-MH) cohort – linkable survey, genetic and routinely collected data for mental health research
Source: BMJ Open. 2024 Jan 12;14(1):e078246. doi: 10.1136/bmjopen-2023-078246 (PMC10806588; doi:10.1136/bmjopen-2023-078246)
Supplement: Author's manuscript [file bmjopen-2023-078246.draft_revisions.pdf]

BMJ Open is committed to open peer review. As part of this commitment we make the peer review history of every article we publish publicly available.

When an article is published we post the peer reviewers' comments and the authors' responses online. We also post the versions of the paper that were used during peer review. These are the versions that the peer review comments apply to.

The versions of the paper that follow are the versions that were submitted during the peer review process. They are not the versions of record or the final published versions. They should not be cited or distributed as the published version of this manuscript.

BMJ Open is an open access journal and the full, final, typeset and author-corrected version of record of the manuscript is available on our site with no access controls, subscription charges or pay-per-view fees (http://bmjopen.bmj.com).

If you have any questions on BMJ Open's open peer review process please email info.bmjopen@bmj.com

BMJ Open

Cohort profile: The Scottish SHARE Mental Health (SHARE-MH) cohort. Linkable survey, genetic and routinely-collected data for mental health research.

Journal:	*BMJ Open*
Manuscript ID	bmjopen-2023-078246
Article Type:	Cohort profile
Date Submitted by the Author:	27-Jul-2023
Complete List of Authors:	Iveson, Matthew; The University of Edinburgh, Centre for Clinical Brain Sciences<br>Ball, Emily; The University of Edinburgh, Centre for Clinical Brain Sciences<br>Doherty, Jason; Washington University in St Louis, Department of Neurology<br>Pugh, Carys; The University of Edinburgh, Advanced Care Research Centre<br>Vashishta, Shobna; University of Dundee, Division of Population Health and Genomics<br>Palmer, Colin; University of Dundee, Division of Population Health and Genomics<br>McIntosh, Andrew; The University of Edinburgh, Centre for Clinical Brain Sciences
Keywords:	MENTAL HEALTH, EPIDEMIOLOGY, GENETICS, Surveys and Questionnaires, PUBLIC HEALTH

SCHOLARONE™
Manuscripts

I, the Submitting Author has the right to grant and does grant on behalf of all authors of the Work (as defined in the below author licence), an exclusive licence and/or a non-exclusive licence for contributions from authors who are: i) UK Crown employees; ii) where BMJ has agreed a CC-BY licence shall apply, and/or iii) in accordance with the terms applicable for US Federal Government officers or employees acting as part of their official duties; on a worldwide, perpetual, irrevocable, royalty-free basis to BMJ Publishing Group Ltd ("BMJ") its licensees and where the relevant Journal is co-owned by BMJ to the co-owners of the Journal, to publish the Work in this journal and any other BMJ products and to exploit all rights, as set out in our licence.

The Submitting Author accepts and understands that any supply made under these terms is made by BMJ to the Submitting Author unless you are acting as an employee on behalf of your employer or a postgraduate student of an affiliated institution which is paying any applicable article publishing charge ("APC") for Open Access articles. Where the Submitting Author wishes to make the Work available on an Open Access basis (and intends to pay the relevant APC), the terms of reuse of such Open Access shall be governed by a Creative Commons licence – details of these licences and which Creative Commons licence will apply to this Work are set out in our licence referred to above.

Other than as permitted in any relevant BMJ Author's Self Archiving Policies, I confirm this Work has not been accepted for publication elsewhere, is not being considered for publication elsewhere and does not duplicate material already published. I confirm all authors consent to publication of this Work and authorise the granting of this licence.

For peer review only

Cohort profile: The Scottish SHARE Mental Health (SHARE-MH) cohort. Linkable survey, genetic and routinely-collected data for mental health research.

Authors: Matthew H. Iveson[1], Emily Ball[1], Jason Doherty[2], Carys Pugh[3], Shobna Vasishta[4], Colin N.A. Palmer[4], Andrew M McIntosh[1]

[1]Centre for Clinical Brain Sciences, The University of Edinburgh, Edinburgh

[2]Department of Neurology, Washington University, St. Louis

[3]Advanced Care Research Centre, The University of Edinburgh, Edinburgh

[4]Division of Population Health and Genomics, University of Dundee, Dundee

Corresponding author:

Matthew H. Iveson

Kennedy Tower,

Morningside Place,

United Kingdom

EH10 5HF

Matthew.Iveson@ed.ac.uk

For peer review only

Abstract

Purpose

The SHARE Mental Health (SHARE-MH) cohort was established to address the paucity of clinical and genetic data available for mental health research. The cohort brings together detailed mental health questionnaire responses, routinely-collected electronic health data and genetic data to provide researchers with an unprecedented linkable dataset. This combination of data sources allows researchers to track mental health longitudinally, across multiple settings. It will be of interest to researchers investigating the genetic and environmental determinants of mental health, the experiences of those interacting with healthcare services, and the overlap between self-reported and clinically-derived mental health outcomes.

Participants

The cohort consists of 9,829 individuals sampled from the Scottish Health Research Register (SHARE). To register for SHARE, individuals had to be over the age of 16 years and living in Scotland. Cohort participants were recruited by email and invited to take part in an online mental health survey. When signing up for SHARE, participants also consented to the use of their electronic health records and genetic data – derived from spare blood samples – for research purposes.

Future plans

The cohort will be improved through linkage to a broader variety of routinely-collected data and to increasing amounts of genetic data obtained through blood sample diversion. We see the SHARE-MH cohort as being used to drive forward novel areas of mental health research and to contribute to global efforts in psychiatric genetics.

Strengths and limitations

- This cohort combines comprehensive survey data with genetic and electronic health data to provide unprecedented detail on mental health within a large sample of adults.
- Combining data sources allows for triangulation of mental health measures – both self-reported and clinically-recorded – and for examining the genetic and psychosocial determinants of mental ill health.
- The mental health survey provides gold-standard measures of mental health, including rarely-available measures of treatment response, substance use, childhood and adult trauma, and wellbeing.

For peer review only

- As a consented cohort about mental health recruited from a health register, the cohort oversamples people with mental health diagnoses versus the general population, and is mostly white, middle-aged and female.
- Health and genetic data is only available for individuals interacting with the healthcare system, missing those that do not seek help or those that seek help in other forms.

Key words: Mental health, data linkage, survey, genetics, health record

Word count: 3259

For peer review only

Introduction

Why was the cohort created?

Mental ill-health is a major public health concern, and mental health conditions are among the leading causes of disease burden across the world (Patel et al., 2018). There is growing research interest in mental ill-health and its causes and consequences. However, these endeavours are frequently hampered by an inadequate data landscape, particularly when compared to the study of physical health conditions such as cancer. It is recognised that more and better mental health data is needed in order to improve our understanding of mental health conditions and to improve the lives of those living with mental ill-health. Recent investments and initiatives have sought to develop the mental health data available, in terms of scale, quality and diversity.

There is a long tradition of assessing mental health through questionnaires. These surveys benefit from deep phenotypic information about the condition of interest, including symptom-level data, and its impact on wellbeing and functioning. Such surveys are often enhanced using genomic data to examine genetic causes and determinants of mental ill-health (e.g., UK Biobank, GLAD) (Davies et al., 2019; Davis et al., 2020). However, recent advancements have led to a growing trend for mental health research using routinely-collected data from various healthcare settings (Davis et al., 2019; Edgcomb & Zima, 2019; John et al., 2016). Routinely-collected data can be provisioned at-scale, often for entire populations, and provide longitudinal information about interactions with healthcare services while minimising participant burden. Notably, the combination of survey, genetic and routinely-collected mental health data is rare. The few studies that do combine these sources are often limited in the detail captured by mental health surveys or in the omission of important but difficult to obtain routinely-collected data such as prescriptions.

This cohort profile introduces and describes the SHARE Mental Health Cohort (SHARE-MH). This cohort combines extensive mental health surveys with routinely-collected national health records and genetic data. The cohort will allow researchers to answer questions about the genetic and environmental causes of mental ill-health, the healthcare journeys of those living with mental health conditions, and the concordance of mental health measures across different sources, among others. It capitalises on existing resources within Scotland to build this resource, including a national research register and blood sampling framework and a national data linkage hub. While the data landscape in Scotland is unique, similar sources of data exist within other UK nations and beyond. Therefore, as well as providing a new data resource for mental health research, this cohort also provides a blueprint for building similar mental health cohorts by enhancing existing resources.

Who is in the cohort?

For peer review only

The cohort was formed from 9,829 individuals, recruited from the Scottish Health Research Register (SHARE) (McKinstry et al., 2017). SHARE is an ongoing voluntary register of individuals who are interested in taking part in health research, with individuals primarily recruited through contact with the Scottish National Health Service (NHS) (e.g., adverts in GP surgeries and outpatient clinics). Any individual living in Scotland and over the age of 16 years may volunteer for the register. Individuals consent to being contacted (primarily by email) by researchers inviting them to take part in health research, as well as the use of their routinely-collected health data for determining their suitability (e.g., where specific populations are required). Individuals can additionally consent to the diversion of their spare blood samples, left over after routine tests, to be stored and genotyped for research use.

What data has been captured?

Cohort creation

A diagram demonstrating the flow of data is shown in Figure 1.

Figure 1. Data flow. Dashed lines indicate processes external to the creation of the SHARE Mental Health cohort. The grey box indicates the Trusted Research Environment operated by the Health Informatics Centre.

--Figure 1 here--

An online mental health survey was used as the foundation of the cohort (Figure 1, left dashed box). This survey was designed to be a detailed assessment of various mental health conditions incorporating questionnaires used in previous studies and common 'gold-standard' research questionnaires (Supplementary Material). Ethical approval for the study and participant materials was given by the NHS Lothian South East Scotland Research Ethics Committee (REC: 19/SS/0074). After designing the survey, researchers in The University of Edinburgh created a list of random research IDs along with unique questionnaire URLs. These IDs and URLs were sent to the Health Informatics Centre (HIC) in The University of Dundee, who act as Trusted Third Party for all research projects using SHARE. HIC created a look-up table matching research IDs to SHARE IDs, genotype IDs, and with Community Health Index (CHI) numbers that are common across Scottish electronic health records. HIC then sent a list of SHARE IDs to be contacted to the SHARE Scotland team. Email invites (Supplementary Material) were sent on 5th February 2021 to all SHARE members with a valid email address (N ~ 179,000). At no point were researchers given access to personal identifiers or contact information for SHARE members.

Using their unique URL, participants were able to start the survey and to complete it over multiple sessions if required. Information sheets were sent along with the initial emails and

For peer review only

were also presented before any survey questions. This included a warning about the nature of the survey contents and advice about survey length (around 30 minutes to complete, depending on their experience with mental health conditions). To start the survey participants were required to confirm informed consent, including for linkage of responses to health records, and that they were 16 years of age or older. Start and end dates and completion times were recorded. All responses to the online survey were collected between 5th February 2021 and 27th November 2021. Deidentified questionnaire responses were collated by researchers at The University of Edinburgh. A copy of the survey data was deposited with the SHARE team (and is held by HIC) and can be requested through SHARE's standard data access process.

For peer review only

Data linkage

After the mental health survey, the cohort was enhanced through linkage to genetic data and routinely-collected health records, covering secondary care interactions and prescriptions. Approval for data linkage for individuals across the whole of Scotland was given under the SHARE research register's existing Public Benefit and Privacy Panel approval (Ref: 2122-0165, approved on 21/12/2021). Researchers sent survey responses and study IDs to HIC, who matched responses to the IDs (genetic IDs and Community Health Index (CHI) IDs) needed for data linkage. Matching health record and genetic data was then extracted from data held by HIC. Health record data included patient demographics, secondary care, prescribing and death records collated at a national scale by Public Health Scotland. Individual payload datasets for each data source were deposited in the Safe Haven operated by HIC. The Safe Haven is an NHS-approved Trusted Research Environment, allowing the secure analysis of sensitive data by researchers while minimising risks to privacy. Access to the data is only given to named and accredited researchers. No primary event level data can leave the Safe Haven, and all outputs are checked for statistical disclosure risks by trained HIC staff.

Demographic data

Demographic information is available both from the mental health survey and from routine health records. In the mental health survey, demographic information was collected at the start in order to ensure the best response rate. The survey collected information on age, gender, ethnicity, relationship status and sexual orientation. It also collected health-related demographics, including disability status, height, weight, diet, smoking history and frequency, and amount of sleep. Finally, the survey collected information on socioeconomic circumstances, including amount of education and attainment and employment.

Routinely-collected data provides information on patient demographics (age, sex, etc.) and area-level measures based on the data zone (a unit of geography defined by the Scottish Government to include roughly equal populations) of their residence as recorded on their CHI record and at admission to secondary care (Scottish Government, 2020). Area measures cover

the rurality and remoteness (Urban-Rural Classification) as well as the relative deprivation of their area (The Scottish Index of Multiple Deprivation; SIMD). Notably, the deprivation measure (SIMD) is calculated by combining indicators of income, employment, health, education, geographic access to services, crime and housing deprivation for each of almost 7,000 data zones in Scotland and then ranking them by their overall deprivation (Scottish Government, 2020). This provides the decile of deprivation for each individual's area, relative to all areas in Scotland, from most to least deprived.

Genetic data

Extracted records also included genetic data derived from a programme of blood sample diversion, which individuals may sign up to upon registering for SHARE. Currently, around 90% of individuals registered with SHARE consent to blood sample diversion. Participants agree to blood samples that are left over from routine care (e.g., when blood tests are ordered by a general practitioner) being sent to a local NHS Scotland Tissue Bank for storage and processing. Samples are processed for genetic data using the Illumina Global Screening Array (https://emea.illumina.com), with the resulting genetic data passed to HIC for retention. Note that this is an ongoing programme, and more genetic data is being added as more samples are diverted and processed. Currently, the blood sample diversion programme operates at the largest hospitals in Scotland, including NHS Tayside, NHS Fife, NHS Grampian, NHS Greater Glasgow and Clyde and NHS Lothian health boards. Rollout of the programme to other health boards is ongoing. Diverted samples are currently available for over 100,00 (around 30%) of consenting SHARE participants. At the time of writing 318 members of the SHARE-MH cohort had genetic data available, though this will rise substantially as more blood samples are diverted and more samples are genotyped. More details on the programme of blood sample diversion and genomic processing can be found in the SHARE cohort profile (McKinstry et al., 2017).

Mental health data

The mental health survey included several detailed questionnaires. These coved topics including self-reported diagnoses, depression, suicide, psychosis, anxiety, substance use (alcohol, tobacco, vaping, as well as illegal and non-prescribed substances), adulthood trauma, childhood trauma, serious life events, stress and post-traumatic stress disorder, the impact of mental ill-health, personality and wellbeing. Questionnaires also collected information on episodes of mental ill-health, their length, frequency and seasonality. Standardised questionnaires were included (e.g., Composite International Diagnostic Interview - Short Form (CIDI-SF), Mood Disorder Questionnaire (MDQ), Alcohol Use Disorders Identification Test (AUDIT), Childhood Trauma Screener (CTS-5), PTSD Checklist (PCL-S), Work and Social Adjustment Scale (WSAS), Eysenck Personality Questionnaire – Revised (EPQ-R)) alongside

For peer review only

questionnaires included in other studies of mental health (UK Biobank mental health online questionnaire; GLAD study online questionnaire). These were chosen to enable multi-cohort studies, greatly increasing the power to investigate risk factors associated with mental illness, and to facilitate cross-cohort comparisons.

Mental health survey responses were enhanced with data from routinely-collected records. These records covered admissions to secondary care settings (Scottish Morbidity Records; SMR) – including outpatient (SMR00; 1996 onwards), general/acute inpatient (SMR01; 1981 onwards), and mental health inpatient (SMR04; 1981 onwards) admissions – as well as community prescribing (Prescribing Information System; 2009 onwards). Records also covered date and cause of death (NHS Death Records; National Records of Scotland Death Records).

For peer review only

Collaboration

Access to the mental health survey data alone can be requested from the University of Edinburgh under Creative Commons Attribution 4.0 International Public License (https://doi.org/10.7488/ds/7491). Access to the survey data for linkage purposes can be obtained through a data access application submitted to the SHARE team. Note that linkage of the questionnaire data to health records may be covered under existing approvals obtained by SHARE or may require separate Public Benefit and Privacy Panel approval, depending on the nature and scale of the linkage. The role of HIC in the linkage and provision of data within the Safe Haven necessitates a Data Processing Agreement with the research organisation. SHARE and HIC operate a cost recovery model to enable research support.

Cohort description

Table 1 describes the SHARE-MH cohort in terms of their demographics, mental health survey response and linkage to health records.

Table 1. Summary of demographic for the SHARE-MH cohort (N = 9,829)

	N Missing	N	Mean (SD)
Age in years	53		57.16 (14.03)
Gender	70		
Female		6165	
Male		3564	
Other		30	
Sexual orientation	177		
Heterosexual		9178	

Homosexual		246	
Bisexual		204	
Other		24	
Relationship status	82		
Single		1129	
Relationship		1442	
Married/Civil Partnership		5895	
Other		1281	
Highest Educational Qualification	157		
None		527	
Standard Grade		842	
Higher Grade		644	
SVQ Level 1/2		391	
SVQ Level 3		455	
SVQ Level 4		1307	
Degree/Professional		5506	
Employment status	190		
Full-time		3120	
Part-time		1517	
Retired		3740	
Other		405	
None		706	
Unemployed		151	
Ethnicity	241		
White		9486	
Mixed		50	
Asian		41	
Other		11	
Smoking status	1263		
Current smoker		669	
Past smoker		3196	
Never smoked		4701	
Alcohol status	1162		
Current drinker		6901	
Stopped within last 12 months		271	
Stopped over 12 months ago		1117	
Never		378	
Illicit drug status	1287		
No		6001	
Yes		2541	
Scottish Index of Multiple Deprivation decile	756		
1 – Most deprived		361	
2		526	
3		581	
4		674	
5		755	
6		884	
7		1053	

For peer review only

8		1288	
9		1373	
10 – Least deprived		1578	

The SHARE-MH cohort broadly resembled the SHARE cohort from which it was selected. An early profile of the wider SHARE register membership indicated that members were predominantly female, middle-aged individuals, though relatively diverse in terms of ethnicity and deprivation (McKinstry et al., 2017). The SHARE-MH cohort also consisted of predominantly female, middle-aged, heterosexual individuals. Indeed, over a third of the Mental Health cohort were retired. Examining SIMD deciles, most participants were from affluent (less deprived) areas of Scotland (1 = 4.0%, 2 = 5.8%, 3 = 6.4%, 4 = 7.4%, 5 = 8.3%, 6 = 9.8%, 7 = 11.6%, 8 = 14.2%, 9 = 15.1%, 10 = 17.4%). Notably, proportionally fewer individuals from deprived areas were represented in the sample, relative to both the SHARE register (1 = 9.3%, 2 = 9.9%, 3 = 9.5%, 4 = 11.1%, 5 = 11.9%, 6 = 9.3%, 7 = 8.4%, 8 = 9.9%, 9 = 10.4%, 10 = 10.3%)(SHARE team, Personal Communication, July 17, 2023) and the wider Scottish population (1 = 9.1%, 2 = 9.6%, 3 = 9.4%, 4 = 9.9%, 5 = 9.9%, 6 = 10.2%, 7 = 10.4%, 8 = 10.7%, 9 = 10.5%, 10 = 10.3%)(National Records of Scotland, 2022).

Physical and Mental Health

The mental health of participants, as self-reported in the Mental Health survey, are summarised in Table 2. Notably, the majority of the cohort did not report any mental health diagnoses, with over 75% reporting being disability-free. This being said, there was a wide variety of mental health diagnoses being endorsed by those living with mental ill health.

Table 2. Summary of mental health from the Mental Health survey (N = 9,829)

	N Missing	N	Mean (SD)
Mental Health survey responses	0		
Complete		8359	
Incomplete		1470	
Disability status	416		
None		7213	
Physical only		1197	
Mental only		414	
Both		589	
Sought/received professional help	385		
No		4325	
Yes		5119	
Self-harm	450		
No		8062	

For peer review only

Yes		1317	
Self-reported diagnoses	136		
Addiction		207	
Anxiety		2641	
Attention deficit and hyperactivity disorder		45	
Autism		80	
Bipolar		134	
Depression		2845	
Eating disorder		250	
Obsessive compulsive disorder		149	
Personality		103	
Phobia		208	
Psychosis		40	
Number of diagnoses	136		
0		5640	
1		2146	
2		1386	
3		365	
4		105	
5+		51	

Routinely-collected health records

A brief summary of the SHARE-MH cohort in terms of their routinely-collected health records is presented in Table 3.

Table 3. Number of participants and records identified within routinely-collected health data, as of May 2023

Dataset	N patients	N records	Date of first record
Outpatient (SMR00)	9690	332713	April 1996
General Inpatient (SMR01)	8438	65787	January 1981
Mental Health Inpatient (SMR04)	231	769	December 1994
Community Prescriptions (PIS)	9754	1869736	January 2009
Death records	143	143	February 2021
Demographic (CHI)	9829	9829	July 1948

The most frequent speciality (i.e., discipline) among outpatient records was trauma and orthopaedic surgery (8.7%), followed by dermatology (7.9%) and ophthalmology (7.5%). General psychiatry admissions made up 5.0% of outpatient records. The most frequent operations were unspecified diagnostic testing (4.4%), diagnostic endoscopic examination of the upper gastrointestinal tract (4.0%) and other diagnostic endoscopic examination of the nasopharynx (3.5%).

For peer review only

The most frequent specialities among general inpatient records were general medicine (14.5%), general surgery (12.3%) and trauma and orthopaedic surgery (8.1%). General psychiatry admissions made up less than 1% of general inpatient records. The most frequent diagnostic International Classification of Diseases (ICD)-10 codes included malignant neoplasm of the breast (2.0%), unspecified chest pain (1.4%), and unspecified cataract (1.4%). This broadly reflects the wider SHARE register, with diagnoses of malignancy (around 6%) and heart failure (around 8%) among the most common.

Mental health inpatient admissions consisted of general psychiatry (92.7%), psychiatry of old age (4.0%) and adolescent psychiatry (3.3%) specialities. The most frequent diagnostic ICD-10 codes included an unspecified single episode major depressive disorder (14.9%), emotionally unstable personality disorder (13.5%), and unspecified bipolar disorder (6.2%).

Community prescribing records consisted mainly of prescriptions related to the cardiovascular system (British National Formulary (BNF) chapter 2; 22.7%), central nervous system (BNF chapter 4; 21.0%) and endocrine system (BNF chapter 6; 11.5%). The most frequently dispensed medications included omeprazole (4.0%), levothyroxine (3.6%) and atorvastatin (2.3%). Of the sample, over 5000 individuals (50%) were prescribed an antidepressant according to electronic prescribing records, with antidepressants making up 7.5% of all prescribing records. The rate of antidepressant prescribing was much higher than that of the wider SHARE register (25% of individuals) and the Scottish population (around 18% of individuals)(Public Health Scotland, 2021), indicating oversampling of antidepressant users. In the present sample, the distribution of specific antidepressants broadly resembled that of the Scottish population (Public Health Scotland, 2021), with amitriptyline – the most frequent – making up around 2% of all records.

Strengths and limitations

One of the main strengths of the SHARE-MH cohort is the ability to link detailed survey data with genetic and electronic health data. This allows for triangulation of mental health measures over multiple sources of data – both self-reported and clinically-recorded. The mental health survey provides gold-standard measures of mental health, including rarely-available measures of substance use, childhood and adult trauma, and wellbeing. The combination of genetic and routinely-collected health data is particularly unique, and allows researchers to examine the genetic determinants of novel mental health phenotypes, such as treatment exposure and healthcare utilisation. The cohort also provides an opportunity to contribute to mental health consortia and initiatives, such as the Psychiatric Genetics Consortium. It is important to note that the data sources cover more than just mental health, allowing the examination of mental-physical comorbidity and of psychosocial causes and consequences of mental ill health.

For peer review only

The SHARE-MH cohort is limited by the representativeness of the sample. Firstly, participants were recruited to the SHARE register through healthcare settings, and so the SHARE register itself likely captures a relatively ill portion of the Scottish population. Furthermore, while the SHARE register appears to represent the socioeconomic and ethnic composition of the wider Scottish population it over represents middle-aged, female individuals (McKinstry et al., 2017), similar to other consented health studies (e.g., the UK Biobank) (Fry et al., 2017). Secondly, as SHARE participants were told that the current study focussed on mental health, mental ill health may be overrepresented in this cohort. Indeed, over 40% of the cohort self-reported a mental health diagnosis, larger than recent estimates of the prevalence of mental ill-health in the Scottish population (around 34%) (Scottish Public Health Observatory, 2023). Similarly, around 50% of the cohort had been prescribed (and dispensed) an antidepressant, much higher than the Scottish population (around 18% of individuals)(Public Health Scotland, 2021). Notably, enriching for mental ill health and medication use may benefit particular types of approach, such as psychiatric genetics. Finally, there is some variation in the availability of linked data. Health and genetic data are only available for individuals interacting with the healthcare system. This misses individuals that experience mental ill health but do not seek help (e.g., those with mild symptoms), or those that seek help in other forms (e.g., talking therapies).

Future work

By incorporating survey, genetic and electronic health data there are a variety of research questions that can be addressed, across the fields of depression, anxiety, substance use, childhood and adult trauma and stress. One important area is examining the alignment of treatment experiences – captured by the questionnaire – with actual treatment trajectories – captured by health records. For example, subjective experiences of treatment for depression aligned with depression-related healthcare interactions and antidepressant prescribing can provide fine-grained information on treatment effectiveness. Research-grade measures from the mental health questionnaire are also being used to benchmark recent data-driven mental health phenotypes, such as those from the Health Data Research UK Phenotype Library (Chapman et al., 2021). Incorporating genetic data can also help to identify subgroups of individuals that respond (or are resistant) to certain treatments. This kind of information can be used to investigate personalised medicine and can help inform sample selection for clinical trials.

There is also ongoing work to increase the amount and breadth of routinely-collected data that can be linked to the cohort. Most notably is the increasing amount of genetic data and number of genotyped participants as sample diversion programmes continue to scale up. There is also work to incorporate novel routinely-collected health datasets, linkable through the same unique CHI number, with national primary care records (i.e., GP data) recently added. Furthermore, recent initiatives such as Research Data Scotland (https://www.researchdata.scot) are helping to increase the variety of available and linkable

For peer review only

data beyond health, including census, education and environmental data. These types of data will be especially useful for examining socioeconomic determinants and mediators of mental ill-health as well as the impact of mental ill-health on education and employment. Finally, there is also a drive to make data access easier and more secure by connecting Trusted Research Environments together. Notably, this allows linkage of new sources of data and federation of analyses across multiple cohorts without the need to move data (Gao et al., 2022; Kavianpour et al., 2022).

For peer review only

Data sharing statement

De-identified mental health survey data is available from the research team upon request. Linked survey, genetic and routinely-collected health data is available upon approval by the SHARE register team, according to their standard access protocol.

Funding declaration

This work was supported by the Wellcome Trust (Refs: 220857/Z/20/Z,226770/Z/22/Z and 216767/Z/19/Z), by UK Research and Innovation (Ref: MR/W014386/1) and by the European Union Horizon 2020 programme (Ref: 847776).

Contributorship statement

CP, MHI and AMM were responsible for designing the cohort and the mental health survey. CP, MHI, SV, CH and CNAP were involved in distributing the mental health survey to SHARE register participants. MHI and JD collated and cleaned responses to the survey. MHI, JD and EB produced statistical output summarising the cohort. MHI drafted and revised the manuscript. All authors gave feedback on drafts for manuscript revision.

Competing interests statement

CNAP is a co-founder of the SHARE Research Register. Other authors report no competing interests.

Patient and Public Involvement

A stakeholder advisory group, including experts by lived experience and those involved in delivering mental healthcare, were involved when planning the creation of the cohort and consulted regarding the use of electronic health records for mental health research. We also piloted the mental health survey with health service users and used their feedback to further shape the survey and its administration.

Acknowledgements

The authors would like to thank the individuals on the SHARE register for their interest in health research and the participants of the SHARE-MH cohort for providing their time and data. The authors would also like to thank the staff at the Health Informatics Centre Dundee,

For peer review only

particularly Chris Hall, who helped coordinate the indexing of survey, genetic and routinely-collected health data.

For peer review only

References

Chapman, M., Mumtaz, S., Rasmussen, L. V., Karwath, A., Gkoutos, G. V., Gao, C., Thayer, D., Pacheco, J. A., Parkinson, H., Richesson, R. L., Jefferson, E., Denaxas, S., & Curcin, V. (2021). Desiderata for the development of next-generation electronic health record phenotype libraries. *GigaScience*, *10*(9), giab059. https://doi.org/10.1093/gigascience/giab059

Davies, M. R., Kalsi, G., Armour, C., Jones, I. R., McIntosh, A. M., Smith, D. J., Walters, J. T. R., Bradley, J. R., Kingston, N., Ashford, S., Beange, I., Brailean, A., Cleare, A. J., Coleman, J. R. I., Curtis, C. J., Curzons, S. C. B., Davis, K. A. S., Dowey, L. R. C., Gault, V. A., … Breen, G. (2019). The Genetic Links to Anxiety and Depression (GLAD) Study: Online recruitment into the largest recontactable study of depression and anxiety. *Behaviour Research and Therapy*, *123*, 103503. https://doi.org/10.1016/j.brat.2019.103503

Davis, K. A. S., Coleman, J. R. I., Adams, M., Allen, N., Breen, G., Cullen, B., Dickens, C., Fox, E., Graham, N., Holliday, J., Howard, L. M., John, A., Lee, W., McCabe, R., McIntosh, A., Pearsall, R., Smith, D. J., Sudlow, C., Ward, J., … Hotopf, M. (2020). Mental health in UK Biobank – development, implementation and results from an online questionnaire completed by 157 366 participants: A reanalysis. *BJPsych Open*, *6*(2), e18. https://doi.org/10.1192/bjo.2019.100

Davis, K. A. S., Cullen, B., Adams, M., Brailean, A., Breen, G., Coleman, J. R. I., Dregan, A., Gaspar, H. A., Hübel, C., Lee, W., McIntosh, A. M., Nolan, J., Pearsall, R., & Hotopf, M. (2019). Indicators of mental disorders in UK Biobank—A comparison of approaches. *International Journal of Methods in Psychiatric Research*, *28*(3). https://doi.org/10.1002/mpr.1796

For peer review only

Edgcomb, J. B., & Zima, B. (2019). Machine Learning, Natural Language Processing, and the Electronic Health Record: Innovations in Mental Health Services Research. *Psychiatric Services*, *70*(4), 346–349. https://doi.org/10.1176/appi.ps.201800401

Fry, A., Littlejohns, T. J., Sudlow, C., Doherty, N., Adamska, L., Sprosen, T., Collins, R., & Allen, N. E. (2017). Comparison of Sociodemographic and Health-Related Characteristics of UK Biobank Participants With Those of the General Population. *American Journal of Epidemiology*, *186*(9), 1026–1034. https://doi.org/10.1093/aje/kwx246

Gao, C., McGilchrist, M., Mumtaz, S., Hall, C., Anderson, L. A., Zurowski, J., Gordon, S., Lumsden, J., Munro, V., Wozniak, A., Sibley, M., Banks, C., Duncan, C., Linksted, P., Hume, A., Stables, C. L., Mayor, C., Caldwell, J., Wilde, K., … Jefferson, E. (2022). A National Network of Safe Havens: Scottish Perspective. *Journal of Medical Internet Research*, *24*(3), e31684. https://doi.org/10.2196/31684

John, A., McGregor, J., Fone, D., Dunstan, F., Cornish, R., Lyons, R. A., & Lloyd, K. R. (2016). Case-finding for common mental disorders of anxiety and depression in primary care: An external validation of routinely collected data. *BMC Medical Informatics and Decision Making*, *16*(1), 35. https://doi.org/10.1186/s12911-016-0274-7

Kavianpour, S., Sutherland, J., Mansouri-Benssassi, E., Coull, N., & Jefferson, E. (2022). Next-Generation Capabilities in Trusted Research Environments: Interview Study. *Journal of Medical Internet Research*, *24*(9), e33720. https://doi.org/10.2196/33720

McKinstry, B., Sullivan, F. M., Vasishta, S., Armstrong, R., Hanley, J., Haughney, J., Philip, S., Smith, B. H., Wood, A., & Palmer, C. N. A. (2017). Cohort profile: The Scottish Research register SHARE. A register of people interested in research participation linked to NHS data sets. *BMJ Open*, *7*(2), e013351. https://doi.org/10.1136/bmjopen-2016-013351

For peer review only

National Records of Scotland. (2022). *Population Estimates by Scottish Index of Multiple Deprivation (SIMD)*. National Records of Scotland. https://www.nrscotland.gov.uk/statistics-and-data/statistics/statistics-by-theme/population/population-estimates/2011-based-special-area-population-estimates/population-estimates-by-simd-2016

Patel, V., Saxena, S., Lund, C., Thornicroft, G., Baingana, F., Bolton, P., Chisholm, D., Collins, P. Y., Cooper, J. L., Eaton, J., Herrman, H., Herzallah, M. M., Huang, Y., Jordans, M. J. D., Kleinman, A., Medina-Mora, M. E., Morgan, E., Niaz, U., Omigbodun, O., … UnÜtzer, Jü. (2018). The Lancet Commission on global mental health and sustainable development. *The Lancet*, *392*(10157), 1553–1598. https://doi.org/10.1016/S0140-6736(18)31612-X

Public Health Scotland. (2021). *Medicines used in Mental Health*. Public Health Scotland. https://publichealthscotland.scot/media/5941/2021-02-23-prescribingmentalhealth-report.pdf

Scottish Government. (2020). *Scottish Index of Multiple Deprivation 2020: Introduction*. https://www.gov.scot/publications/scottish-index-multiple-deprivation-2020/

Scottish Public Health Observatory. (2023). *Scottish Public Health Observatory Profiles Tool* [Computer software]. https://scotland.shinyapps.io/ScotPHO_profiles_tool/

For peer review only

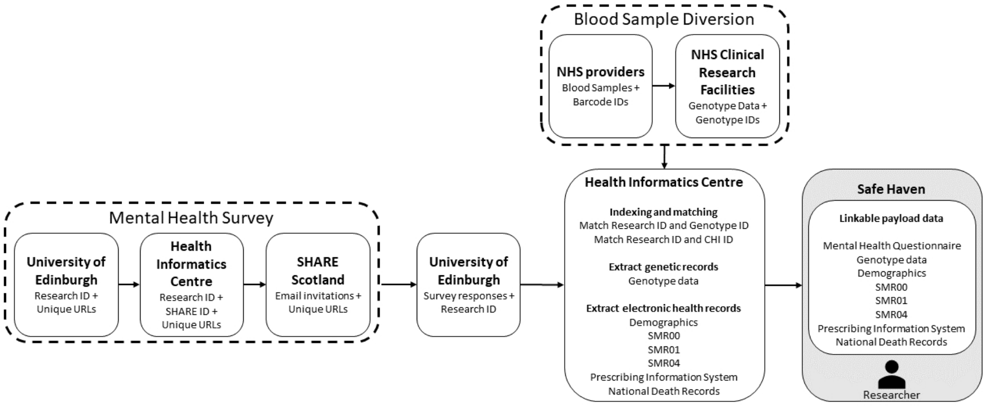

Figure 1. Data flow. Dashed lines indicate processes external to the creation of the SHARE Mental Health cohort. The grey box indicates the Trusted Research Environment operated by the Health Informatics Centre.

855x481mm (38 x 38 DPI)

Mental Health Data Science Scotland

Block 1: Introduction

Mental health questionnaire for SHARE participants

Questions about your experiences in relation to mental health

Thank you for taking the time to assist us. We are hoping to understand the causes of mental health conditions so that we can try to improve the lives of people who are affected by mental illness. We are interested in your responses whether or not you have ever experienced mental illness yourself.

We are interested in knowing more about the stresses and strains of life and your mental health. Some of the questions are sensitive and may be difficult to answer. Please try to answer as many questions as you can and give the most accurate answers possible. However, if you **cannot** answer a question, you can choose the **"don't know"** or **"prefer not to answer"** option where available. In some instances, it is possible to skip sections and move on.

All responses to questions asked in this questionnaire will be kept strictly confidential, they will only be shared with researchers in a way that means you cannot be identified.

To make it easier for you to participate, you can start the questionnaire and if you leave part way, it will remember where you were when you return to it later. You can see your progress through the survey in the blue bar at the top of the page.

Information on where to find help for the issues covered in this questionnaire is given below and at the end of the survey.

If you feel you need any further help with the issues in this questionnaire, we recommend talking

it through with someone you trust, including your GP. For more information and support, visit The Scottish Association for Mental Health (SAMH).

If you have been upset by remembering sexual violence or any other kind of abuse/crime, please contact support groups for aid or information, such as Scottish Women's Aid (for urgent assistance, give them a call on 0800 027 1234), ManKind (01823 334244 10am-4pm), LGBTdomesticabuse or online Victim Support Scotland.

If you are in distress and need urgent help or advice, please contact someone as soon as possible, such as the **Samaritans on 116 123**

If you would like to take part in the research, please read and select **all** of the statements below.

- ☐ I understand that continuing with this questionnaire is **voluntary** and that I can stop at any point without giving a reason
- ☐ I confirm that I am 16 or older
- ☐ I understand that researchers will combine my answers with an anonymised version of my health records for use in research
- ☐ I consent to take part in this study

Block 2: Basics

What age are you now?

- ○ [ ] Current age (years)

Which **gender** do you **identify** with?

- ○ Male
- ○ Female
- ○ Non-binary
- ○ Another gender identity
- ○ Don't know
- ○ Prefer not to answer

For peer review only - http://bmjopen.bmj.com/site/about/guidelines.xhtml

Thank you for your interest in taking part in this study. We appreciate your support and willingness to take part in research. You have received this message because you have said that you are **under 16** and we are currently only recruiting participants who are **16 or over**. We would be very interested in hearing from you once you are 16.

Block 3: Demographics/lifestyle

What is your **current marital/relationship status**?

- Single
- Relationship (not living together)
- Relationship (living together)
- Married
- Civil partnership
- Separated
- Divorced
- Widowed
- Other
- Prefer not to say

What is your **sexual orientation?**

- Heterosexual/straight
- Homosexual/gay/lesbian
- Bisexual/bi
- Another sexual orientation
- Don't know
- Prefer not to answer

Do you consider yourself to have a disability?

- Yes, a physical disability
- Yes, a mental health disability
- Yes, both a physical and mental health disability
- No, I don't have a disability
- Don't know
- Prefer not to answer

For peer review only - http://bmjopen.bmj.com/site/about/guidelines.xhtml

How old were you when you left High School?

- ○ [ ] Age (years)
- ○ I am still in High School

How old were you when you left full-time education?

- ○ [ ] Age (years)
- ○ I am still in full-time education

Which of these qualifications do you have?

*Please select **ALL** that apply*

- ☐ O Grade, Standard Grade, Access 3 Cluster, Intermediate 1 or 3, GCSE, CSE, Senior Certificate or equivalent
- ☐ SCE Higher Grade, Higher, Advanced Higher, CSYS, A Level, AS Level, Advanced Senior Certificate or equivalent
- ☐ GSVQ Foundation or Intermediate, SVQ level 1 or 2, SCOTVEC Module, City and Guilds Craft or equivalent
- ☐ GSVQ Advanced, SVQ level 3, ONC, OND, SCOTVEC National Diploma, City and Guilds Advanced Craft or equivalent
- ☐ HNC, HND, SVQ level 4 or equivalent
- ☐ Degree, Postgraduate qualifications, Masters, PhD, SVQ level 5 or equivalent
- ☐ Professional qualifications (for example, teaching, nursing, accountancy)
- ☐ Other school qualifications not already mentioned (including foreign qualifications)
- ☐ Other post-school but pre-Higher Education qualifications not already mentioned (including foreign qualifications)
- ☐ Other Higher Education qualifications not already mentioned (including foreign qualifications)
- ☐ No qualifications

What is your **current employment status**?
You can only choose one option so please choose the one that best describes your employment status.

- ○ In paid employment or self-employed - Full Time

For peer review only - http://bmjopen.bmj.com/site/about/guidelines.xhtml

- In paid employment or self-employed - Part Time
- Retired (may include volunteering and/or caring for home and/or family)
- Looking after home and/or family
- Unable to work because of sickness or disability
- Unemployed
- Doing unpaid or voluntary work
- Full or part-time student
- None of the above
- Prefer not to answer

How would you describe your ethnic origin?

- White
- Mixed
- Asian or Asian British
- Black or Black British
- Other ethnic group
- Don't know
- Prefer not to answer

The next question is about your **height**. Please select your preferred units of measure.

- Feet/inches
- Centimetres

What is your **current** height?
(If you are unsure, please give your best estimate)

- [ ] ______ Height (cm)
- [ ] ______ Height (feet)
- [ ] ______ Height (inches)

The next question is about your **weight**. Please select your preferred units of measure.

- Stones/pounds
- Kilograms

For peer review only - http://bmjopen.bmj.com/site/about/guidelines.xhtml

What is your **current** weight?
(If you are unsure, please give your best estimate)

- ☐ Weight (kg)
- ☐ Weight (stones)
- ☐ Weight (pounds)

Has a health professional (doctor, nurse, physiotherapist or similar) ever told you that you were overweight?
Please do not include times during pregnancy

- ○ Yes
- ○ No
- ○ Don't know
- ○ Prefer not to answer

How many **hours per day** do you **sleep** on average?
Please include night time sleep **and** day time naps.

- ○ Average number of hours per day

Do you have a specific diet?

- ○ Vegetarian
- ○ Vegan
- ○ Pescatarian
- ○ Other
- ○ No
- ○ Prefer not to answer

Block 4: Mental Health History

This section has some questions about your mental health

In your life, have you suffered from a **period of mental distress** that prevented you from doing your **usual activities**?

- Yes
- No
- Don't know
- Prefer not to answer

In your life, did you **seek or receive help** from a **professional** (medical doctor, psychologist, social worker, counsellor, nurse, clergy or other helping professional) for mental distress, psychological problems or unusual experiences?

- Yes
- No
- Don't know
- Prefer not to answer

Have you ever hurt yourself on purpose in any way (e.g. by taking an overdose of pills, or by cutting yourself)?

- Yes
- No
- Don't know
- Prefer not to answer

Have **YOU** ever been **diagnosed** with one or more of the following illnesses or conditions by a **professional**, even if you don't have it currently?
By professional we mean: any doctor, nurse or person with specialist training (such as a psychologist, psychiatrist etc.).

Please include illnesses or conditions even if you did not need treatment for them or if you did not agree with the diagnosis.

*Please select **ALL** that apply*

- Addiction or substance use disorder (such as alcohol addiction, drug addiction)

- ☐ Anxiety (such as generalised anxiety disorder, social anxiety, panic disorder or post-traumatic stress disorder)
- ☐ Autism, Asperger's or autistic spectrum disorder
- ☐ Attention deficit or attention deficit and hyperactivity disorder (ADD/ADHD)
- ☐ Bipolar disorder, mania, hypomania, manic-depression
- ☐ Depression (such as major depressive disorder, seasonal affective disorder, postnatal depression)
- ☐ Eating disorder (such as anorexia, bulimia, binge eating disorder)
- ☐ Obsessive-compulsive disorder (OCD)
- ☐ Personality disorder
- ☐ Phobia (such as social phobia, agoraphobia or another phobia, for example a disabling fear of heights or spiders
- ☐ Schizophrenia or psychosis
- ☐ None of the above
- ☐ Don't know
- ☐ Prefer not to answer

You mentioned that you have been diagnosed with anxiety (such as generalised anxiety disorder, social anxiety, panic disorder or post-traumatic stress disorder). *Please tell us what type(s) of anxiety you have been diagnosed with*

- ☐ Anxiety, nerves or generalised anxiety disorder
- ☐ Panic disorder
- ☐ Post-traumatic stress disorder (PTSD)
- ☐ Social anxiety
- ☐ Don't know
- ☐ Prefer not to answer

You mentioned that you have been diagnosed with depression (such as major depressive disorder, seasonal affective disorder or postnatal depression). *Please tell us what type(s) of depression you have been diagnosed with*

- ☐ Major depressive disorder
- ☐ Postnatal depression (depression during pregnancy for you or your partner or within a year of giving birth)
- ☐ Seasonal Affective Disorder (SAD)
- ☐ Don't know
- ☐ Prefer not to answer

You mentioned that you have had postnatal depression. Were you the person who was pregnant?

- Yes, I was pregnant
- No, my partner was pregnant

You mentioned that you have been diagnosed with an eating disorder.
Please tell us which eating disorder(s) you have been diagnosed with

- Anorexia nervosa
- Atypical anorexia nervosa
- Bulimia nervosa
- Psychological over-eating or binge eating
- Don't know
- Prefer not to answer

You mentioned that you have been diagnosed with a personality disorder.
Please tell us which personality disorder(s) you have been diagnosed with

- Borderline personality disorder
- Other personality disorder
- Don't know
- Prefer not to answer

You mentioned that you have been diagnosed with a phobia.
Please tell us which phobia(s) you have been diagnosed with

- Social phobia
- Agoraphobia
- Other phobia (such as a disabling fear of heights or spiders)
- Don't know
- Prefer not to answer

You mentioned that you have been diagnosed with schizophrenia or another type of psychosis.

For peer review only - http://bmjopen.bmj.com/site/about/guidelines.xhtml

Please tell us which type of psychosis you have been diagnosed with.

- ☐ Schizophrenia
- ☐ Other type of psychosis
- ☐ Don't know
- ☐ Prefer not to answer

Block 5: Mood

The next section asks a few questions about your **mood** and **feelings.**

Have you ever had a time in your life when you have felt sad, blue or depressed for **two weeks or more** in a row?

- ◯ Yes
- ◯ No
- ◯ Prefer not to answer

Have you ever had a time in your life lasting **two weeks or more** when you lost interest in most things like hobbies, work, or activities that usually give you pleasure?

- ◯ Yes
- ◯ No
- ◯ Prefer not to answer

In the next section, we would like to know more about your **depression or low mood**. If you think you may find this distressing, you can **skip** these questions.

- ◯ Continue
- ◯ Skip

Please think of the **two-week period** in your life when your feelings of depression or loss of interest were worst

How much of the day did these feelings usually last?

- ◯ All day long
- ◯ Most of the day
- ◯ About half of the day
- ◯ Less than half of the day

For peer review only - http://bmjopen.bmj.com/site/about/guidelines.xhtml

- Don’t know
- Prefer not to answer

Please think of the **two-week period** in your life when your feelings of depression or loss of interest were worst

Did you feel this way:

- Every day
- Almost every day
- Less often
- Don’t know
- Prefer not to answer

Please think of the **two-week period** in your life when your feelings of depression or loss of interest were worst

Did you feel more **tired** out or **low on energy** than is usual for you?

- Yes
- No
- Don't know
- Prefer not to say

Did you gain or lose weight without trying, or did you stay about the same weight?

- Gained weight
- Lost weight
- Both gained and lost weight during the same episode
- Stayed about the same or was on a diet
- Don't know
- Prefer not to say

Did your weight change by about **10lbs (4kg)** or more?

- Yes
- No
- Don't know
- Prefer not to say

Please think of the **two-week period** in your life when your feelings of depression or loss of interest were worst

Did your **sleep** change?

- Yes
- No
- Don't know
- Prefer not to say

Was that:

Please answer all four questions

	Yes	No
Trouble falling asleep	◯	◯
Waking too early	◯	◯
Sleeping too much	◯	◯
Both sleeping too much and too little during the same episode of depression or loss of interest	◯	◯

How many **hours per day** did you **sleep on average** during the episode of depression or loss of interest? Please include night time sleep **and** day time naps.

◯ [ ] Hours sleep per day

How many **hours per day** did you used to **sleep** on average when you were **not depressed**? Please include night time sleep **and** day time naps.

◯ [ ] Hours sleep per day

Please think of the **two-week period** in your life when your feelings of depression or loss of interest were worst

Did you experience a **change** in your appetite?

- Increased appetite
- Decreased appetite

For peer review only - http://bmjopen.bmj.com/site/about/guidelines.xhtml

○ Both increased and decreased appetite during the same episode of depression or loss interest
○ No changes in appetite
○ Don't know
○ Prefer not to answer

Please think of the **two-week period** in your life when your feelings of depression or loss of interest were worst

Did your **mood brighten** in response to **positive events**?

○ Yes
○ No
○ Don't know
○ Prefer not to answer

Please think of the **two-week period** in your life when your feelings of depression or loss of interest were worst

Were you **overly sensitive** to comments from other people ?

○ Yes, and this **significantly** impaired your social or work relationships
○ Yes, but this did **not significantly** impair your social or work relationships
○ No
○ Don’t know
○ Prefer not to answer

Please think of the **two-week period** in your life when your feelings of depression or loss of interest were worst

Was your mood **worse:**

☐ In the **morning**
☐ In the **afternoon**
☐ At **night**
☐ My mood did **not** vary
☐ Don't know
☐ Prefer not to answer

Please think of the **two-week period** in your life when your feelings of depression or loss of interest were worst

Did you have a lot more **trouble concentrating than usual?**

- Yes
- No
- Don't know
- Prefer not to answer

Please think of the **two-week period** in your life when your feelings of depression or loss of interest were worst

Did you notice a change in your interest in sex?

- Yes
- No
- Don't know
- Prefer not to answer

Please think of the **two-week period** in your life when your feelings of depression or loss of interest were worst

People sometimes **feel down** on themselves, no good, worthless. Did you feel this way?

- Yes
- No
- Don't know
- Prefer not to answer

Please think of the **two-week period** in your life when your feelings of depression or loss of interest were worst

Did you **think** a lot about **death** – either your own, or someone else's, or death in general?

- Yes
- No
- Don't know
- Prefer not to answer

Did you feel so low you thought a lot about ending your life?

For peer review only - http://bmjopen.bmj.com/site/about/guidelines.xhtml

- Yes
- No
- Don't know
- Prefer not to answer

Did you make a suicide plan?

- Yes
- No
- Don't know
- Prefer not to answer

Did you attempt to end your life?

- Yes
- No
- Don't know
- Prefer not to answer

Please think of the **two-week period** in your life when your feelings of depression or loss of interest were worst

Think about your **roles** at the time of this episode, including study/employment, childcare, housework and leisure pursuits. How much did your feelings of depression or loss of interest **interfere** with your life or activities?

- A lot
- Some
- A little
- Not at all
- Don't know
- Prefer not to answer

How many periods of depression or low mood have you had in your life **lasting two or more weeks?**

- One

- Two or three
- More than three
- Don't know
- Prefer not to answer

Please estimate the number of separate episodes of depression or low mood in your life lasting two or more weeks:

- [ ] Estimate of number of depressive episodes

Did any of these episodes occur **during pregnancy (for you or your partner)** or **within the first year of giving birth?** Or has it been suggested you had postnatal depression?

(We are interested in your answer regardless of gender)

- Yes
- No
- Don't know
- Prefer not to answer

You mentioned that you have had postnatal depression. Were you the person who was pregnant?”

- Yes, I was pregnant
- No, my partner was pregnant

About how old were you the **first** time you had a **period of two weeks** like this? (Whether or not you received any help for it.)
An approximate age is fine

- [ ] Approx. age of **first** depressive episode (years)

About how old were you the **last** time you had a **period of two weeks** like this? (Whether or not you received any help for it.)
An approximate age is fine

- [ ] Approx. age of **most recent** depressive episode (years)

Did you **ever** tell a professional about these problems? *(Medical doctor, psychologist, social worker, counselor, nurse, clergy, or other helping professional)*

- Yes
- No
- Don't know
- Prefer not to say

Did you **ever** try or are you **currently** trying any of the following for these problems? *(Please select **ALL** that apply)*

- Medications prescribed to you for at least two weeks
- Unprescribed medication more than once
- Drugs or alcohol more than once
- Psychotherapy or other talking therapy more than once
- None of the above
- Prefer not to answer

Did you take your medication **as advised**?

- Yes, at least mostly
- No
- Don't know
- Prefer not to answer

Do you think that the prescribed medications helped or are helping you?

- Yes
- No
- Don't know
- Prefer not to answer

You mention that one (or more) medications was helpful or not helpful to you. Some commonly prescribed medications are given below. Please tell us which you found helpful or unhelpful.

The same medications can have many different names. Each that we mention has been given its common name (e.g. Citalopram) and some common brand names (e.g. celexa, cipramil)

*Please choose an option for **ALL** medications*

	Medication **helpful**	Medication **somewhat helpful**	Medication **not helpful**	Not relevant (medication **no[t] prescribed**)
Amitriptyline<br>*Also called: Domical, Elavil, Lentizol, Tryptizol*	○	○	○	○
Citalopram<br>*Also called: Celexa, Cipramil*	○	○	○	○
Fluoxetine<br>*Also called: Prozac, Prozep, Olena, Oxactin*	○	○	○	○
Mirtazapine<br>*Also called: Zispin*	○	○	○	○
Sertraline<br>*Also called: Lustral*	○	○	○	○
Venlafaxine<br>*Also called: Efexor*	○	○	○	○
Other medication not mentioned above	○	○	○	○

You mention that you have tried or are trying psychotherapy or another talking therapy more than once. Please tell us which you have tried and whether you found it helpful or unhelpful.

*Please choose an option for **ALL** therapy types*

	Therapy **helpful**	Therapy **somewhat helpful**	Therapy **not helpful**	Not relevant (not tried this therapy)	Don't know	Prefer not to answe[r]
Counselling	○	○	○	○	○	○
Mindfulness	○	○	○	○	○	○

For peer review only - http://bmjopen.bmj.com/site/about/guidelines.xhtml

	Therapy **helpful**	Therapy **somewhat helpful**	Therapy **not helpful**	Not relevant (not tried this therapy)	Don't know	Prefer not to answer
[illegible]	◯	◯	◯	◯	◯	◯
Relationship therapy	◯	◯	◯	◯	◯	◯
Group therapy	◯	◯	◯	◯	◯	◯
Guided self-help	◯	◯	◯	◯	◯	◯
Family therapy	◯	◯	◯	◯	◯	◯
Cognitive behavioural therapy (CBT)	◯	◯	◯	◯	◯	◯
Workshops	◯	◯	◯	◯	◯	◯
Online therapy	◯	◯	◯	◯	◯	◯
Other	◯	◯	◯	◯	◯	◯

We have been asking you about a two-week period but **altogether** over your life, **how long** have your feelings of depression or loss of interest lasted?

If you are unsure, please estimate

- ◯ Less than a month
- ◯ Between **one** and **three months**
- ◯ **Over** three months, but **less** than six months
- ◯ **Over** six months, but **less** than 12 months
- ◯ Between **one** and **two years**
- ◯ **Over** two years
- ◯ Don't know
- ◯ Prefer not to answer

Was your depression or loss of interest **worse:**

- ☐ In the **Spring**
- ☐ In the **Summer**
- ☐ In the **Autumn**
- ☐ In the **Winter**
- ☐ My mood did **not** vary with the time of year
- ☐ Don't know
- ☐ Prefer not to answer

Block 6: Bipolar illness and psychosis

Now we would like to know about some different symptoms in your **lifetime**. Please answer each question to the best of your ability.

Has there **ever** been a **period of time** when you were not your usual self and...

(Please respond to each item)

	Yes	No
...you felt so **good** or so **hyper** that other people thought you were not your normal self or you were so hyper that you got into **trouble**?	○	○
...you were so **irritable** that you shouted at people or started fights or arguments?	○	○
...you felt **much more self-confident** than usual?	○	○
...you got much **less** sleep than usual and found you didn't really **miss** it?	○	○
…you were much more talkative or spoke **much faster** than usual?	○	○
...thoughts raced through your head or you couldn't slow your mind down?	○	○
...you were so **easily distracted** by things around you that you had trouble concentrating or staying on track?	○	○
...you had **much more energy** than usual?	○	○
...you were **much more active** or did many more things than usual?	○	○
...you were **much more social** or outgoing than usual, for example, you telephoned friends in the middle of the night?	○	○
...you were **much more interested** in sex than usual?	○	○
...you did things that were unusual for you or that other people might have thought were **excessive, foolish**, or **risky**?	○	○
...spending money got you or your family into trouble?	○	○

You ticked **'yes'** to more than one of the previous symptoms – have **several** of these ever happened during the **same period of time**?

- ○ Yes
- ○ No

You indicated that you experienced the following. Please select **all** that occurred during the **same period of time**

- ☐ Prefer not to answer
- ☐ Don't know

What is the **longest time** that these "high" or "irritable" periods have lasted?

- ○ Less than 24 hours

- At least a day, but less than a week
- A week or more
- Don't know
- Prefer not to answer

How much of a **problem** did any of these cause you - like being unable to work; having family, money or legal troubles; getting into arguments or fights?

- No problem
- Minor problem
- Moderate problem
- Serious problem
- Prefer not to answer

Block 7: Anxiety or nerves

This section asks some questions about **anxiety** or **nerves**.

People differ a lot in how much they worry about things. Did you ever have a time when you worried a lot **more than most people** would in your situation?

- Yes
- No
- Don't know
- Prefer not to answer

Have you ever had a period lasting **one month or longer** when most of the time you felt worried, tense, or anxious?

- Yes
- No
- Don't know
- Prefer not to answer

What is the **longest period of time** that this kind of worrying has ever continued?
(If you are not sure of the exact amount of time, please give an estimate)

For peer review only - http://bmjopen.bmj.com/site/about/guidelines.xhtml

☐ ____ Years

☐ ____ Months

Please think of the period in your life when you have felt **worried, tense, anxious,** or **more worried** than most people would in your situation. This could be in the past, or it could be continuing now.
The following questions refer to this period of time.

During that period, was your worry **stronger** than in other people?

- Yes
- No
- Don't know
- Prefer not to answer

Please think of the period in your life when you have felt **worried, tense, anxious,** or **more worried** than most people would in your situation. This could be in the past, or it could be continuing now.
The following questions refer to this period of time.

Did you worry **most days**?

- Yes
- No
- Don't know
- Prefer not to answer

Please think of the period in your life when you have felt **worried, tense, anxious,** or **more worried** than most people would in your situation. This could be in the past, or it could be continuing now.
The following questions refer to this period of time.

Did you usually worry about **one particular thing**, such as your job security or the failing health of a loved one, or **more than one thing**?

- One thing
- More than one thing
- Don't know
- Prefer not to say

Please think of the period in your life when you have felt **worried, tense, anxious,** or **more worried** than most people would in your situation. This could be in the past, or it could be continuing now.
The following questions refer to this period of time.

Did you find it **difficult to stop** worrying?

- Yes
- No
- Don't know
- Prefer not to answer

Please think of the period in your life when you have felt **worried, tense, anxious,** or **more worried** than most people would in your situation. This could be in the past, or it could be continuing now.
The following questions refer to this period of time.

Did you ever have different worries on your mind **at the same time**?

- Yes
- No
- Don't know
- Prefer not to answer

Please think of the period in your life when you have felt **worried, tense, anxious,** or **more worried** than most people would in your situation. This could be in the past, or it could be continuing now.
The following questions refer to this period of time.

How often was your worry so strong that you **couldn't** put it out of your mind no matter how hard you tried?

- Often
- Sometimes
- Rarely
- Never
- Don't know
- Prefer not to answer

Please think of the period in your life when you have felt **worried, tense, anxious,** or **more worried** than most people would in your situation. This could be in the past, or it could be continuing now.
The following questions refer to this period of time.

How often did you find it **difficult to control** your worry?

- Often
- Sometimes
- Rarely
- Never
- Don't know
- Prefer not to answer

When you were **worried or anxious**, were you also:

	Yes	No	Don't know
Restless?	○	○	○
Keyed up or on edge?	○	○	○
Easily tired?	○	○	○
Having difficulty keeping your mind on what you were doing?	○	○	○
More irritable than usual?	○	○	○
Having tense, sore, or aching muscles?	○	○	○
Often having trouble falling or staying asleep?	○	○	○

Did you **ever** tell a **professional** about these problems? *(medical doctor, psychologist, social worker, counsellor, nurse, clergy, or other helping professional)*

- Yes

- No
- Don't know
- Prefer not to answer

Regarding times in your life when you have felt **worried, tense or anxious:** Did you **ever** use the following for the worry or the problems it caused?
Please include any treatments that you have already told us about under 'depression' if they were also for anxiety.
(Please select all that apply)

- Medications prescribed to you for at least two weeks
- Specific anti-anxiety medication prescribed to you for at least one week
- Unprescribed medication more than once
- Drugs or alcohol more than once
- Psychotherapy or other talking therapy more than once
- None of the above
- Prefer not to answer

Did you take your medication **as advised**?

- Yes, at least mostly
- No
- Don't know
- Prefer not to answer

You mention that you have tried or are trying psychotherapy or another talking therapy more than once. Please tell us which you have tried and whether you found it helpful or unhelpful.

	Therapy **helpful**	Therapy **not helpful**	Not relevant (not tried this therapy)	Don't know	Prefer not to answer
Counselling	○	○	○	○	○
Mindfullness	○	○	○	○	○
Relationship therapy	○	○	○	○	○
Group therapy	○	○	○	○	○

For peer review only - http://bmjopen.bmj.com/site/about/guidelines.xhtml

	Therapy **helpful**	Therapy **not helpful**	Not relevant (not tried this therapy)	Don't know	Prefer not to answer
Guided self-help	○	○	○	○	○
Family therapy	○	○	○	○	○
Cognitive behavioural therapy (CBT)	○	○	○	○	○
Workshops	○	○	○	○	○
Online therapy	○	○	○	○	○
Other	○	○	○	○	○

Regarding times in your life when you have felt **worried, tense or anxious:**

Think about your **roles** at the time of this episode, including study/employment, childcare and housework, leisure pursuits. How much did these problems **interfere** with your life or activities?

- ○ A lot
- ○ Some
- ○ A little
- ○ Not at all
- ○ Prefer not to answer

Block 8: Alcohol

In this section, we would like to ask you about alcohol, as we think it may influence mental health.

Your answers will remain confidential so please be honest.

Please state how much you agree with the following statement.

A little bit of alcohol can be good for you

- ○ Very much
- ○ Quite a bit
- ○ Somewhat
- ○ A little bit

- Not at all
- Don't know
- Prefer not to answer

The next questions are about how frequently you drink alcoholic beverages.

Have you ever had an alcoholic drink?

- Yes, I currently drink alcohol
- Yes, but I stopped drinking within the last 12 months
- Yes, but I stopped drinking more than 12 months ago
- No, I have never had an alcoholic drink

Please think about your use of alcoholic drinks in the **last year**.

How often do you have a drink containing **alcohol?**

- Never
- Monthly or less
- 2 to 4 times a month
- 2 to 3 times a week
- 4 or more times a week

Please think about your use of alcohol drinks in the **last year.**

How many units of alcohol do you normally drink in a week?

Alcoholic drink	Approx. units
1 pint of ordinary beer, cider or lager	2
1 pint of extra strength beer /lager	3
1 can of cider	2

For peer review only - http://bmjopen.bmj.com/site/about/guidelines.xhtml

1 litre of cider	5
1 glass of wine (175ml)	2
1 bottle of wine	10
1 bottle of fortified wine (e.g. buckfast or port) (75cl)	11
1 litre of fortified wine (e.g. buckfast or port)	15
1 small glass of sherry	1
1 bottle of sherry (100cl)	15
1 pub measure of spirits (25ml)	1
1 bottle of spirits (75cl)	30
1 bottle of alcopops	2

○ ________ Approximate number of units (per week)

Please think about your use of alcoholic drinks in the **last year**.

How many drinks containing alcohol do you have on a typical day when you are drinking?

○ 1 or 2
○ 3 or 4
○ 5 or 6
○ 7, 8 or 9
○ 10 or more

Please think about your use of alcoholic drinks in the **last year**.

How often do you have six or more drinks on one occasion?

○ Never

- Less than monthly
- Monthly
- Weekly
- Daily or almost daily

How often during the **last year** have you found that you were **not able to stop** drinking once you had started?

- Never
- Less than monthly
- Monthly
- Weekly
- Daily or almost daily

How often during the **last year** have you failed to do what was normally expected from you because of drinking?

- Never
- Less than monthly
- Monthly
- Weekly
- Daily or almost daily

How often during the **last year** have you **needed** a first drink in the **morning** to get yourself going after a heavy drinking session?

- Never
- Less than monthly
- Monthly
- Weekly
- Daily or almost daily

How often during the **last year** have you had a feeling of **guilt or remorse** after drinking?

- Never
- Less than monthly
- Monthly

- Weekly
- Daily or almost daily

How often during the **last year** have you been **unable** to remember what happened the night before because you had been drinking?

- Never
- Less than monthly
- Monthly
- Weekly
- Daily or almost daily

We will now ask you some questions about drinking during your **lifetime**.

Have **you or someone else** been injured as a result of your drinking?

- No
- Yes, but not in the last year
- Yes, during the last year

Has a **relative or friend or a doctor** or another health worker been **concerned** about your drinking or suggested you cut down?

- No
- Yes, but not in the last year
- Yes, during the last year

About how old were you first started drinking alcohol, not counting small tastes or sips?

- ________ Approximate age (years)

Do you think you have ever been a heavy drinker?

- Yes, now
- Yes, now **and** in the past
- Yes, in the past **but not now**

- No
- Don't know
- Prefer not to answer

Think about the period in your life when you drank the most. How old were you when that period began?

- [ ] Approximate age (years)

How long did that period last?

- [ ] Approximate length of time (years)

During that period when you drank the most, about how often did you drink?

- Monthly or less
- 2 to 4 times a month
- 2 to 3 times a week
- 4 or more times a week
- Don't know
- Prefer not to answer

During that period when you drank the most, how many **units** of alcohol did you drink on a **typical day** when you are drinking?

**Alcoholic drink**	**Approx. units**
1 pint of ordinary beer, cider or lager	2
1 pint of extra strength beer /lager	3
1 can of cider	2
1 litre of cider	5
1 glass of wine (175ml)	2
1 bottle of wine (75cl)	10
1 bottle of fortified wine (e.g. buckfast or port) (75cl)	11
1 litre of fortified wine (e.g. buckfast or port)	15
1 small glass of sherry	1

For peer review only - http://bmjopen.bmj.com/site/about/guidelines.xhtml

Alcoholic drink	Approx. units
1 bottle of sherry (100cl)	15
1 pub measure of spirits (25ml)	1
1 bottle of spirits (75cl)	30
1 bottle of alcopops	2

○ [ ] Approximate number of units (per day)

How much alcohol do you have to drink before you start to feel its effects?

(If you are not sure, your best guess is fine)

○ More than other people (drink doesn't seem to affect me as much as other people)
○ The same as other people
○ Less than other people (I am easily affected by alcohol)
○ Don't know
○ Prefer not to answer

How bad are your hangovers from drinking alcohol?

○ Very bad
○ Quite bad
○ Not bad at all
○ I don't drink enough to get a hangover
○ Don't know
○ Prefer not to answer

We will now ask you some questions about your reasons for drinking alcohol

Please state how often the following statement is true.

I feel more comfortable around others when I drink

(please think about now and in the past)

○ Always or almost always

- Often
- Sometimes
- Rarely
- Never
- Don't know
- Prefer not to answer

Please state how often the following statement is true.

I sleep better after I drink

(please think about now and in the past)

- Always or almost always
- Often
- Sometimes
- Rarely
- Never
- Don't know
- Prefer not to answer

Please state how often the following statement is true.

I drink because I am nervous or anxious

(please think about now and in the past)

- Always or almost always
- Often
- Sometimes
- Rarely
- Never
- Don't know
- Prefer not to answer

Please state how often the following statement is true.

For peer review only - http://bmjopen.bmj.com/site/about/guidelines.xhtml

I drink because I am depressed

(please think about now and in the past)

- Always or almost always
- Often
- Sometimes
- Rarely
- Never
- Don't know
- Prefer not to answer

Please state how often the following statement is true.

I drink because I am in physical pain

(please think about now and in the past)

- Always or almost always
- Often
- Sometimes
- Rarely
- Never
- Don't know
- Prefer not to answer

Please state how often the following statement is true.

I drink because I am angry, annoyed or bad-tempered

(please think about now and in the past)

- Always or almost always
- Often
- Sometimes
- Rarely

- Never
- Don't know
- Prefer not to answer

Please state how often the following statement is true.

I drink to escape my memories or thoughts

(please think about now and in the past)

- Always or almost always
- Often
- Sometimes
- Rarely
- Never
- Don't know
- Prefer not to answer

Please state how often the following statement is true.

I drink because I feel tense or stressed

(please think about now and in the past)

- Always or almost always
- Often
- Sometimes
- Rarely
- Never
- Don't know
- Prefer not to answer

Block 9: Smoking

In this section, we will ask you about smoking

Please select the box that best describes you

- ◯ I smoke now
- ◯ I used to smoke
- ◯ I have never smoked
- ◯ Prefer not to answer

How many years has it been since you stopped smoking?
(An approximate answer is fine)

◯ ____ Years since stopped smoking

Approximately how many cigarettes do you or did you smoke each day?

◯ ____ Number of cigarettes per day

How many years have you / did you smoke?

◯ ____ Years smoking

How many hours per week are you **exposed** to other people's tobacco smoke (*passive smoking*)?

◯ ____ Hours per week

Have you ever tried an e-cigarette or vaping device?

- ◯ Yes
- ◯ No
- ◯ Don't know
- ◯ Prefer not to answer

How often do you currently use an e-cigarette or vaping device?

- ◯ Daily or almost daily

- Less than daily, but at least once a week
- Less than weekly, but at least once a month
- Less than monthly
- Not at all
- Don't know
- Prefer not to answer

Have you ever used and e-cigarette or vaping device daily for a month or more?

- Yes
- No
- Don't know
- Prefer not to answer

Block 10: Illegal substances

In this section, we would like to ask you about illegal substances, as we think they may influence mental health.

Your answers will remain confidential so please be honest.

Have you **ever** taken any **drugs** other than those prescribed for you for medical reasons?
*For example **cannabis, cocaine, speed, amphetamines, ecstasy, LSD, heroin** or **methadone** or substances previously called **"legal" highs** such as **spice**.*

We do **not** mean **prescription drugs** and will ask about these later.

- Yes
- No
- Prefer not to answer

You mention that you have taken drugs in your lifetime. Please tell us how often you took them when you were using them most.

*Please choose an option for **ALL** groups of drugs*

	Never	Once or twice only	Monthly or less	2 to 4 times a month	2 to 3 times a week	4 or more times a week	Prefer not to answer
Heroin or methadone	○	○	○	○	○	○	○
Cocaine	○	○	○	○	○	○	○
Cannabis or marijuana	○	○	○	○	○	○	○
Speed, amphetamines or ecstasy	○	○	○	○	○	○	○
Spice or other substance previously called a "legal" high	○	○	○	○	○	○	○
Magic mushrooms or LSD	○	○	○	○	○	○	○
Ketamine	○	○	○	○	○	○	○
Other illegal drug	○	○	○	○	○	○	○

And how long did you take heroin or methadone for?
If you are still taking them, please give the time so far.
If you are unsure an estimate is fine.

- ○ ______ Years
- ○ Less than one year
- ○ ______ I am still taking heroin or methadone. Years so far
- ○ Prefer not to answer

And how long did you take cocaine for?
If you are still taking it, please give the time so far.
If you are unsure an estimate is fine.

- ○ ______ Years
- ○ Less than one year
- ○ ______ I am still taking cocaine. Years so far
- ○ Prefer not to answer

And how long did you take cannabis or marijuana for?
If you are still taking them, please give the time so far.
If you are unsure an estimate is fine.

For peer review only - http://bmjopen.bmj.com/site/about/guidelines.xhtml

- [ ] Years
- Less than one year
- [ ] I am still taking cannabis or marijuana. Years so far
- Prefer not to answer

And how long did you take speed, amphetamines or ecstasy for?
If you are still taking them, please give the time so far.
If you are unsure an estimate is fine.

- [ ] Years
- Less than one year
- I am still taking speed, amphetamines or ecstasy. Years so far [ ]
- Prefer not to answer

And how long did you take magic mushrooms or LSD for?
If you are still taking them, please give the time so far.
If you are unsure an estimate is fine.

- [ ] Years
- Less than one year
- [ ] I am still taking magic mushrooms or LSD. Years so far
- Prefer not to answer

And how long did you take ketamine for?
If you are still taking them, please give the time so far.
If you are unsure an estimate is fine.

- [ ] Years
- Less than one year
- [ ] I am still taking ketamine. Years so far
- Prefer not to answer

And how long did you take spice or other substance previously called a "legal" high for?

If you are still taking them, please give the time so far.

If you are unsure an estimate is fine.

- ◯ [ ] Years
- ◯ Less than one year
- ◯ I am still taking spice or other substance previously called a "legal" high. Years so far [ ]
- ◯ Prefer not to answer

Have you **ever** taken **prescription drugs** that were **not prescribed for you?**

*For example: **valium, diazepam, temazepam, other 'benzos'** or **opioids** such as **fentanyl, tramadol, buprenorphine, oxycodone**.*

- ◯ Yes
- ◯ No
- ◯ Prefer not to answer

You mention that you have taken prescription drugs (not prescribed for you) in your lifetime. Please tell us how often you took them when you were using them most.

*Please choose an option for **ALL** groups of drugs*

	Never	Once or twice only	Monthly or less	2 to 4 times a month	2 to 3 times a week	4 or more times a week	Prefer not to answer
Valium, diazepam, temazepam or other *"benzo"*	◯	◯	◯	◯	◯	◯	◯
Fentanyl, tramadol, buprenorphine, oxycodone or other opiod (excluding heroin and methdaone)	◯	◯	◯	◯	◯	◯	◯
Other prescription drug that was not prescribed for you	◯	◯	◯	◯	◯	◯	◯

And how long did you take valium, diazepam, temazepam or another "benzo" for?

If you are still taking them, please give the time so far.

If you are unsure an estimate is fine.

- [ ] Years
- Less than one year
- I am still taking valium, diazepam, temazepam or another "benzo". Years so far
- Prefer not to answer

And how long did you take an opioid such as fentanyl, tramadol, buprenorphine or oxycodone for?

If you are still taking them, please give the time so far.

If you are unsure an estimate is fine.

- [ ] Years
- Less than one year
- I am still taking an opioid such as fentanyl, tramadol, buprenorphine or oxycodone. Years so far
- Prefer not to answer

Block 11: Trauma

This section asks about your childhood and the stresses and strains of adult life. Some people may find these questions distressing. If you would prefer not to answer these questions, please use a **skip** button below.

If you are distressed by remembering past crime/abuse, please visit Victim Support Scotland

- Continue
- Skip childhood questions
- Skip adult questions
- Skip childhood and adult questions

When I was growing up...

For peer review only - http://bmjopen.bmj.com/site/about/guidelines.xhtml

	Never true	Rarely true	Sometimes true	Often true	Very often true	Prefer not to answer
I felt loved	O	O	O	O	O	O
People in my family hit me so hard that it left me with bruises or marks	O	O	O	O	O	O
I felt that someone in my family hated me	O	O	O	O	O	O
Someone molested me (sexually)	O	O	O	O	O	O
There was someone to take me to the doctor if I needed it	O	O	O	O	O	O

If you feel distressed from remembering past crime/abuse, please visit Victim Support Scotland. *If you have been upset by remembering domestic or sexual violence, there is information available at Scottish Women's Aid (for urgent assistance, give them a call on 0800 027 1234),* ManKind (01823 334244 10am-4pm) *or* LGBTdomesticabuse

Since I was sixteen...

	Never	Yes, but not in the last 12 months	Yes, within the last 12 months	Prefer not to answer
I have been in a confiding relationship	O	O	O	O
A partner or ex-partner deliberately hit me or used violence in any other way	O	O	O	O
A partner or ex-partner repeatedly belittled me to the extent that I felt worthless	O	O	O	O
A partner or ex-partner sexually interfered with me, or forced me to have sex against my wishes	O	O	O	O
I have struggled to find the money to pay my rent/ mortgage payment	O	O	O	O

If you feel distressed from remembering past crime/abuse, please visit Victim Support Scotland. *If you have been upset by remembering domestic or sexual violence, there*

is information available at [Scottish Women's Aid](#) (for urgent assistance, give them a call on 0800 027 1234), ManKind (01823 334244 10am-4pm) *or* LGBTdomesticabuse

You said that since you were sixteen, you have been in a confiding relationship. Please indicate how often this was true.

- Rarely true
- Sometimes true
- Often true
- Very often true
- Prefer not to answer

You said that since you were sixteen, a partner or ex-partner deliberately hit you or used violence in another way. Please indicate how often this was true.

- Rarely true
- Sometimes true
- Often true
- Very often true
- Prefer not to answer

If you feel distressed from remembering past crime/abuse, please visit Victim Support Scotland. *If you have been upset by remembering domestic or sexual violence, there is information available at* ***Scottish Women's Aid*** *(for urgent assistance, give them a call on 0800 027 1234),* ManKind (01823 334244 10am-4pm) *or* LGBTdomesticabuse

You said that since you were sixteen, a partner or ex-partner repeatedly belittled you to the extent that you felt worthless. Please indicate how often this was true.

- Rarely true
- Sometimes true
- Often true
- Very often true
- Prefer not to answer

If you feel distressed from remembering past crime/abuse, please visit Victim Support Scotland. *If you have been upset by remembering domestic or sexual violence, there is information available at Scottish Women's Aid (for urgent assistance, give them a call on 0800 027 1234),* ManKind (01823 334244 10am-4pm) *or* LGBTdomesticabuse

You said that since you were sixteen, a partner or ex-partner sexually interfered with you, or forced you to have sex against your wishes. Please indicate how often this was true.

- ○ Rarely true
- ○ Sometimes true
- ○ Often true
- ○ Very often true
- ○ Prefer not to answer

If you feel distressed from remembering past crime/abuse, please visit Victim Support Scotland. *If you have been upset by remembering domestic or sexual violence, there is information available at Scottish Women's Aid (for urgent assistance, give them a call on 0800 027 1234),* ManKind (01823 334244 10am-4pm) *or* LGBTdomesticabuse

You said that since you were sixteen, you have struggled to find the money to pay your rent/mortgage payment. Please indicate how often this was true

- ○ Rarely true
- ○ Sometimes true
- ○ Often true
- ○ Very often true
- ○ Prefer not to answer

In your life, have you...

	Never	Yes, but not in the last 12 months	Yes, within the last 12 months	Prefer not to answer
Been a victim of a sexual assault, whether by a stranger or someone you knew	○	○	○	○

	Never	Yes, but not in the last 12 months	Yes, within the last 12 months	Prefer not to answer
Been attacked, mugged, robbed, or been the victim of a physically violent crime	○	○	○	○
Been in a serious accident that you believed to be life-threatening at the time	○	○	○	○
Witnessed a sudden violent death (e.g. murder, suicide, aftermath of an accident)	○	○	○	○
Been diagnosed with a life-threatening illness	○	○	○	○
Been involved in combat or exposed to a war-zone (either in the military or as a civilian)	○	○	○	○

If you feel distressed from remembering past crime/abuse, please visit Victim Support Scotland. *If you have been upset by remembering domestic or sexual violence, there is information available at Scottish Women's Aid (for urgent assistance, give them a call on 0800 027 1234),* ManKind (01823 334244 10am-4pm) *or* LGBTdomesticabuse

Next, is a list of **problems and complaints** that people sometimes have in response to extremely stressful experiences. Please indicate how much you have been bothered by that problem in the **past month**:

	Not at all	A little bit	Moderately	Quite a bit	Extremely
Repeated, disturbing memories, thoughts, or images of a stressful experience?	○	○	○	○	○
Feeling very upset when something reminded you of a stressful experience?	○	○	○	○	○
Avoiding activities or situations because they reminded you of a stressful situation?	○	○	○	○	○
Feeling distant or cut off from other people?	○	○	○	○	○
Feeling irritable or having angry outbursts?	○	○	○	○	○
Difficulty concentrating?	○	○	○	○	○

For peer review only - http://bmjopen.bmj.com/site/about/guidelines.xhtml

If you feel distressed from remembering past crime/abuse, please visit Victim Support Scotland. *If you have been upset by remembering domestic or sexual violence, there is information available at Scottish Women's Aid (for urgent assistance, give them a call on 0800 027 1234),* ManKind (01823 334244 10am-4pm) *or* LGBTdomesticabuse

Block 12: Impact

Questions about your work and social life

People's problems sometimes **affect** their ability to do certain day-to-day tasks in their lives.

To rate your problems look at each section and determine on the scale provided **how much** your problem **impairs/impaired your ability** to carry out the activity.

Please complete this section thinking about problems you have been asked about like **anxiety, depression and sleep problems.**

If you have had multiple issues, please think of the issue that has impacted you **most severely**.

Because of my problem my ability to **work** is/was impaired. If you are retired or choose not to have a job for reasons unrelated to your problem, please tick NA *(not applicable).*

- Not at all
- Slightly
- Moderately
- Markedly
- Very severely, I cannot/could not work
- NA
- Prefer not to answer

Because of my problem my **home management** *(Cleaning, tidying, shopping, cooking, looking after home/children, paying bills)* is/was impaired.

- Not at all
- Slightly
- Moderately
- Markedly
- Very severely
- Prefer not to answer

For peer review only - http://bmjopen.bmj.com/site/about/guidelines.xhtml

Because of my problem my **social leisure activities** *(with other people eg. parties, bars, clubs, outings, visits, dating, home entertainment)* are impaired.

- Not at all
- Slightly
- Moderately
- Markedly
- Very severely
- Prefer not to answer

Because of my problem my **private leisure activities** *(done alone, such as reading, gardening, collecting, sewing, walking alone)* are impaired.

- Not at all
- Slightly
- Moderately
- Markedly
- Very severely
- Prefer not to answer

Because of my problem **my ability to form and maintain close relationships** with others, including those I live with, is impaired.

- Not at all
- Slightly
- Moderately
- Markedly
- Very severely
- Prefer not to answer

Block 13: Eyseneck

The following 12 questions are about your feelings

Does your mood often go up and down?

- Yes

No

Do you ever feel 'just miserable' for no reason?

Yes

No

Are you an irritable person?

Yes

No

Are your feelings easily hurt?

Yes

No

Do you often feel 'fed-up'?

Yes

No

Would you call yourself a nervous person?

Yes

No

Are you a worrier?

Yes

No

Would you call yourself tense or 'highly strung'?

Yes

No

Do you worry too long after an embarrassing experience?

Yes

No

Do you suffer from nerves?

Yes

No

Do you often feel lonely?

Yes

No

Are you often troubled about feelings of guilt?

Yes

No

Block 14: Subjective wellbeing

This is the **last** section of the survey.
In this section we would like to know how you **feel** about things **in general.**

In general, how **happy** are you?

Extremely happy

Very happy

Moderately happy

Moderately unhappy

Very unhappy

Extremely unhappy

For peer review only - http://bmjopen.bmj.com/site/about/guidelines.xhtml

- Don't know
- Prefer not to answer

In general, how happy are you with your **health?**

- Extremely happy
- Very happy
- Moderately happy
- Moderately unhappy
- Very unhappy
- Extremely unhappy
- Don't know
- Prefer not to answer

To what extent do you feel your life to be **meaningful**?

- An extreme amount
- Very much
- A moderate amount
- A little
- Not at all
- Don't know
- Prefer not to answer

Please submit your survey by pressing the next arrow below

If you feel you need any further help with the issues in this questionnaire, we recommend talking it through with someone you trust, including your GP. For more information and support, visit The Scottish Association for Mental Health (SAMH).

If you have been upset by remembering sexual violence or any other kind of abuse/crime, please contact support groups for aid or information, such as Scottish Women's Aid (for urgent assistance, give them a call on 0800 027 1234), ManKind (01823 334244 10am-4pm), LGBTdomesticabuse or online Victim Support Scotland.

If you are in distress and need urgent help or advice, please contact someone as soon as possible, such as the **Samaritans on 116 123**

Powered by Qualtrics

Contact email and patient information leaflet: Version 03 14/11/19

SHARE Online survey – A mental health survey for Scotland

MHDSS – A mental health survey for Scotland

Dear

You very kindly joined SHARE, the Scottish Health Research Register, as you were interested in hearing about opportunities to help with medical research. We are writing to let you know about a research opportunity that has arisen and that might appeal to you. Please see information from the study team below:

Participation in this research study involves completing **an online survey** about mental health.

You can start the survey by clicking the link below:

https://unique.url.per.participant

You will be able to complete the survey on a home computer, tablet or smart-phone.

Further details about the study are provided below.

For peer review only

Contact email and patient information leaflet: Version 03 14/11/19

MHDSS Mental Health Data Science Scotland

Participant Information Leaflet

What am I being invited to do?

You are invited to take part in an online survey about mental health. The survey answers will be used for health research and there will be no direct compensation for individuals taking part in the survey.

What will the survey involve?

If you decide to take part, you will be asked a series of questions about yourself and any history of mental ill-health you may have had. We will then ask for some details about things that may have an impact on mental health, such as drinking alcohol, taking illegal substances and difficult experiences in your life. Finally, there will be some questions about your feelings. The questions will not include asking for your email or any other identifying information.

Please be aware that the survey concerns mental health and some questions may be distressing for some participants. **Participation is voluntary** and you can leave the survey at any time by closing your internet browser. We also give participants the option to skip some sections should they wish.

How long will the survey take? Can I come back later?

On average, the whole survey takes approximately **15 minutes to complete**. It may take less time if you have limited experience of mental health problems and will probably take **20-25 minutes** if you have more experience with low moods. We do not think it will take anyone more than **one hour**. If you are not able to complete the survey in one go you can **return to it later** by clicking on the link you have been sent (please note you will need to make sure you are using the same computer/device to access the survey). The link you have been sent to the survey is unique so, if more than one person in your household has been sent this email, please only use the link that was sent to you.

What is the research about?

The aim of the research is to better understand why people develop mental illnesses and how that links with their physical health. We will do that by collecting information from participants and linking this with their health records. The information collected will **not** be added to your health record, it will be stored separately and will be only be used for research purposes. It will be **strictly confidential** and only shared with

researchers in a way that means you cannot be identified. In the future, your responses may also be used by other researchers to select you for further studies of interest but your participation would be completely voluntary.

Who is conducting the research?

This survey is being carried out by researchers at the University of Edinburgh and it is funded by a grant from the Medical Research Council (MRC).

What about data security?

The questionnaire deals with sensitive information so every care is being taken with regard to data security. The software we are using to collect answers is called Qualtrics. The company behind this software works with the NHS, companies and universities and has no access to any of the answers that participants give. All answers will be collected, encrypted and stored electronically on an ISO 27001 certified, secure server. They will then be securely transferred to the University of Edinburgh and from there to an NHS data Safe Haven which also uses ISO 27001 certified servers. The information held by the University of Edinburgh will contain no identifying information. Within the Safe Haven, a separate encrypted file will be used to link the responses with anonymised electronic health records. Researchers must then do all of their work within the Safe Haven and cannot remove information about individuals. Results can only be removed about groups of people. If you would like to know more about how we approach data security and how the National Safe Havens work, please visit our website here https://mhdss.ac.uk/what-about-security-and-privacy

Do I have to take part?

No, you do not have to take part in this survey. We will ask you to consent at the beginning of the questionnaire and, if you agree, this would indicate a willingness for your data to be used by the research team. Should you wish to withdraw your data, you can email <<MHDSS email>> and give us the **email address that was used to contact you**. You do not need a reason and we do not need your name or any details of your responses. If your questionnaire answers have already been used in analyses, they cannot be removed because they will have been anonymised before analysis but we will contact the Safe Haven team and they will delete your answers so that they cannot be used again.

Where can I find more information?

You can find out more about the team behind this study and our wider work via our website Mental Health Data Science Scotland or you can email enquiries to the team working with this questionnaire at <<MHDSS email>>.

If you wish to contact someone who has knowledge of the study, but is not part of the team working on this questionnaire, please contact <<External contact>> by emailing <<External’s email address>> or writing to <<External’s postal address>>.

Contact email and patient information leaflet: Version 03 14/11/19

What should I do if I want to make a complaint?

Any concerns should be directed in the first instance to the team at Edinburgh University using <<MHDSS email>>. If you have concerns which you believe have not been addressed then complaints can be made to the Head of the Division of Psychiatry at the University of Edinburgh, <<HoD details>>. Email: <<HoD email address>> or write to <<HoD postal address>>.

For peer review only

STROBE Statement—Checklist of items that should be included in reports of ***cohort studies***

	Item No	Recommendation	Page No
**Title and abstract**	1	(*a*) Indicate the study's design with a commonly used term in the title or the abstract	1
		(*b*) Provide in the abstract an informative and balanced summary of what was done and what was found	2
**Introduction**			
Background/rationale	2	Explain the scientific background and rationale for the investigation being reported	4
Objectives	3	State specific objectives, including any prespecified hypotheses	4
**Methods**			
Study design	4	Present key elements of study design early in the paper	5
Setting	5	Describe the setting, locations, and relevant dates, including periods of recruitment, exposure, follow-up, and data collection	5
Participants	6	(*a*) Give the eligibility criteria, and the sources and methods of selection of participants. Describe methods of follow-up	5
		(*b*) For matched studies, give matching criteria and number of exposed and unexposed	NA
Variables	7	Clearly define all outcomes, exposures, predictors, potential confounders, and effect modifiers. Give diagnostic criteria, if applicable	6-8
Data sources/ measurement	8*	For each variable of interest, give sources of data and details of methods of assessment (measurement). Describe comparability of assessment methods if there is more than one group	6-8
Bias	9	Describe any efforts to address potential sources of bias	5
Study size	10	Explain how the study size was arrived at	5
Quantitative variables	11	Explain how quantitative variables were handled in the analyses. If applicable, describe which groupings were chosen and why	NA
Statistical methods	12	(*a*) Describe all statistical methods, including those used to control for confounding	NA
		(*b*) Describe any methods used to examine subgroups and interactions	NA
		(*c*) Explain how missing data were addressed	NA
		(*d*) If applicable, explain how loss to follow-up was addressed	NA
		(*e*) Describe any sensitivity analyses	NA
**Results**			
Participants	13*	(a) Report numbers of individuals at each stage of study—eg numbers potentially eligible, examined for eligibility, confirmed eligible, included in the study, completing follow-up, and analysed	5-6; 10-11
		(b) Give reasons for non-participation at each stage	5-6; 10-11
		(c) Consider use of a flow diagram	5
Descriptive data	14*	(a) Give characteristics of study participants (eg demographic, clinical, social) and information on exposures and potential confounders	8-12
		(b) Indicate number of participants with missing data for each variable of interest	8-9
		(c) Summarise follow-up time (eg, average and total amount)	11
Outcome data	15*	Report numbers of outcome events or summary measures over time	8-12

For peer review only - http://bmjopen.bmj.com/site/about/guidelines.xhtml

Main results	16	(*a*) Give unadjusted estimates and, if applicable, confounder-adjusted estimates and their precision (eg, 95% confidence interval). Make clear which confounders were adjusted for and why they were included	NA
		(*b*) Report category boundaries when continuous variables were categorized	NA
		(*c*) If relevant, consider translating estimates of relative risk into absolute risk for a meaningful time period	NA
Other analyses	17	Report other analyses done—eg analyses of subgroups and interactions, and sensitivity analyses	NA
**Discussion**			
Key results	18	Summarise key results with reference to study objectives	12
Limitations	19	Discuss limitations of the study, taking into account sources of potential bias or imprecision. Discuss both direction and magnitude of any potential bias	12-13
Interpretation	20	Give a cautious overall interpretation of results considering objectives, limitations, multiplicity of analyses, results from similar studies, and other relevant evidence	12-13
Generalisability	21	Discuss the generalisability (external validity) of the study results	12-13
**Other information**			
Funding	22	Give the source of funding and the role of the funders for the present study and, if applicable, for the original study on which the present article is based	15

*Give information separately for exposed and unexposed groups.

Note: An Explanation and Elaboration article discusses each checklist item and gives methodological background and published examples of transparent reporting. The STROBE checklist is best used in conjunction with this article (freely available on the Web sites of PLoS Medicine at http://www.plosmedicine.org/, Annals of Internal Medicine at http://www.annals.org/, and Epidemiology at http://www.epidem.com/). Information on the STROBE Initiative is available at http://www.strobe-statement.org.

For peer review only

For peer review only - http://bmjopen.bmj.com/site/about/guidelines.xhtml

Cohort profile: The Scottish SHARE Mental Health (SHARE-MH) cohort. Linkable survey, genetic and routinely-collected data for mental health research.

Journal:	*BMJ Open*
Manuscript ID	bmjopen-2023-078246.R1
Article Type:	Cohort profile
Date Submitted by the Author:	28-Nov-2023
Complete List of Authors:	Iveson, Matthew; The University of Edinburgh, Centre for Clinical Brain Sciences<br>Ball, Emily; The University of Edinburgh, Centre for Clinical Brain Sciences<br>Doherty, Jason; Washington University in St Louis, Department of Neurology<br>Pugh, Carys; The University of Edinburgh, Advanced Care Research Centre<br>Vashishta, Shobna; University of Dundee, Division of Population Health and Genomics<br>Palmer, Colin; University of Dundee, Division of Population Health and Genomics<br>McIntosh, Andrew; The University of Edinburgh, Centre for Clinical Brain Sciences
**Primary Subject Heading**:	Mental health
Secondary Subject Heading:	Epidemiology, Genetics and genomics, Health informatics, Research methods
Keywords:	MENTAL HEALTH, EPIDEMIOLOGY, GENETICS, Surveys and Questionnaires

SCHOLARONE™
Manuscripts

I, the Submitting Author has the right to grant and does grant on behalf of all authors of the Work (as defined in the below author licence), an exclusive licence and/or a non-exclusive licence for contributions from authors who are: i) UK Crown employees; ii) where BMJ has agreed a CC-BY licence shall apply, and/or iii) in accordance with the terms applicable for US Federal Government officers or employees acting as part of their official duties; on a worldwide, perpetual, irrevocable, royalty-free basis to BMJ Publishing Group Ltd ("BMJ") its licensees and where the relevant Journal is co-owned by BMJ to the co-owners of the Journal, to publish the Work in this journal and any other BMJ products and to exploit all rights, as set out in our licence.

The Submitting Author accepts and understands that any supply made under these terms is made by BMJ to the Submitting Author unless you are acting as an employee on behalf of your employer or a postgraduate student of an affiliated institution which is paying any applicable article publishing charge ("APC") for Open Access articles. Where the Submitting Author wishes to make the Work available on an Open Access basis (and intends to pay the relevant APC), the terms of reuse of such Open Access shall be governed by a Creative Commons licence – details of these licences and which Creative Commons licence will apply to this Work are set out in our licence referred to above.

Other than as permitted in any relevant BMJ Author's Self Archiving Policies, I confirm this Work has not been accepted for publication elsewhere, is not being considered for publication elsewhere and does not duplicate material already published. I confirm all authors consent to publication of this Work and authorise the granting of this licence.

For peer review only

Cohort profile: The Scottish SHARE Mental Health (SHARE-MH) cohort. Linkable survey, genetic and routinely-collected data for mental health research.

Authors: Matthew H. Iveson[1], Emily Ball[1], Jason Doherty[2], Carys Pugh[3], Shobna Vasishta[4], Colin N.A. Palmer[4], Andrew M McIntosh[1]

[1]Centre for Clinical Brain Sciences, The University of Edinburgh, Edinburgh

[2]Department of Neurology, Washington University, St. Louis

[3]Advanced Care Research Centre, The University of Edinburgh, Edinburgh

[4]Division of Population Health and Genomics, University of Dundee, Dundee

Corresponding author:

Matthew H. Iveson

Kennedy Tower,

Morningside Place,

United Kingdom

EH10 5HF

Matthew.Iveson@ed.ac.uk

For peer review only

Abstract

Purpose

The SHARE Mental Health (SHARE-MH) cohort was established to address the paucity of clinical and genetic data available for mental health research. The cohort brings together detailed mental health questionnaire responses, routinely-collected electronic health data and genetic data to provide researchers with an unprecedented linkable dataset. This combination of data sources allows researchers to track mental health longitudinally, across multiple settings. It will be of interest to researchers investigating the genetic and environmental determinants of mental health, the experiences of those interacting with healthcare services, and the overlap between self-reported and clinically-derived mental health outcomes.

Participants

The cohort consists of individuals sampled from the Scottish Health Research Register (SHARE). To register for SHARE, individuals had to be over the age of 16 years and living in Scotland. Cohort participants were recruited by email and invited to take part in an online mental health survey. When signing up for SHARE, participants also provided written consent to the use of their electronic health records and genetic data – derived from spare blood samples – for research purposes.

Findings to date

From 5th February 2021 to 27th November 2021, 9,829 individuals completed a survey of various mental health topics, capturing information on symptoms, diagnoses, impact and treatment. Survey responses have been made linkable to electronic health records and genetic data using a single patient identifier. Linked data have been used to describe the cohort in terms of their demographics, self-reported mental health, inpatient and outpatient hospitalisations and dispensed prescriptions.

Future plans

The cohort will be improved through linkage to a broader variety of routinely-collected data and to increasing amounts of genetic data obtained through blood sample diversion. We see the SHARE-MH cohort being used to drive forward novel areas of mental health research and to contribute to global efforts in psychiatric genetics.

Strengths and limitations

For peer review only

- This cohort combines comprehensive survey data with genetic and electronic health data to provide unprecedented detail on mental health within a large sample of adults.
- Combining data sources allows for triangulation of mental health measures – both self-reported and clinically-recorded – and for examining the genetic and psychosocial determinants of mental ill health.
- The mental health survey provides gold-standard measures of mental health, including rarely-available measures of treatment response, substance use, childhood and adult trauma, and wellbeing.
- The cohort is recruited from a health register, not the general population, resulting in a risk of selection bias towards those with ill-health.
- Linked health and genetic data is only available for individuals interacting with the healthcare system, resulting in key data that is missing but not at random.

Key words: Mental health, data linkage, survey, genetics, health record

Word count: 3372

For peer review only

Introduction

Mental ill-health is a major public health concern, and mental health conditions are among the leading causes of disease burden across the world [1]. There is growing research interest in mental ill-health and its causes and consequences. However, these endeavours are frequently hampered by an inadequate data landscape in terms of availability, transparency and usability [2]. It is recognised that more and better mental health data is needed in order to improve our understanding of mental health conditions and to improve the lives of those living with mental ill-health. Recent investments and initiatives such as the Health Data Research UK DATAMIND hub (https://datamind.org.uk/) and the Common Measures in Mental Health Science Initiative [3] have sought to develop the mental health data available, in terms of scale, quality and diversity.

In research studies, mental health is commonly assessed using questionnaires, both clinician-rated and self-reported. These questionnaires capture deep experiential and phenotypic information about the condition of interest, including symptom-level data, and its impact on wellbeing and functioning. Such questionnaires, grouped into surveys, are often enhanced using genomic data to examine genetic causes and determinants of mental ill-health (e.g., UK Biobank, GLAD) [4,5]. However, recent advancements have led to a growing trend for mental health research using data collected during routine healthcare from various healthcare settings [6–8]. Routinely-collected data can be provisioned at-scale, often for entire populations, and provide longitudinal information about interactions with healthcare services (diagnoses, treatment, etc.) while minimising participant burden. Notably, the combination of survey, genetic and routinely-collected mental health data is rare. The few studies that do combine these sources are often limited in the detail captured by mental health surveys or in the omission of important but difficult to obtain routinely-collected data such as prescriptions.

This cohort profile introduces and describes the SHARE Mental Health Cohort (SHARE-MH). This cohort combines extensive mental health surveys with routinely-collected national health records and genetic data. The cohort will allow researchers to answer questions about the genetic and environmental causes of mental ill-health, the healthcare journeys of those living with mental health conditions, and the concordance of mental health measures across different sources, among others. It capitalises on existing resources within Scotland to build this resource, including a national research register and blood sampling framework and a national data linkage hub. While the data landscape in Scotland is unique, similar sources of data exist within other UK nations and beyond. Therefore, as well as providing a new data resource for mental health research, this cohort also provides a blueprint for building similar mental health cohorts by enhancing existing resources.

Cohort description

For peer review only

The cohort was formed from 9,829 individuals, recruited from the Scottish Health Research Register (SHARE) [9]. SHARE is an ongoing voluntary register of individuals who are interested in taking part in health research, with individuals primarily recruited through contact with the Scottish National Health Service (NHS) (e.g., adverts in GP surgeries and outpatient clinics). Any individual living in Scotland and over the age of 16 years may volunteer for the register. Individuals provide written consent to being contacted (primarily by email) by researchers inviting them to take part in health research, as well as the use of their routinely-collected health data for determining their suitability (e.g., where specific populations are required). Individuals can additionally consent to the diversion of their spare blood samples, left over after routine tests, to be stored and genotyped for research use.

<u>Cohort creation</u>

A diagram demonstrating the flow of data is shown in Figure 1.

Figure 1. Data flow. Dashed lines indicate processes external to the creation of the SHARE Mental Health cohort. The grey box indicates the Trusted Research Environment operated by the Health Informatics Centre.

--Figure 1 here--

Footnote: SHARE = Scottish Health Research Register; NHS = National Health Service; CHI = Community Health Index; SMR = Scottish Morbidity Records; SMR00 = Outpatients; SMR01 = General/Acute Inpatients; SMR04 = Mental Health Inpatients.

An online mental health survey was used as the foundation of the cohort (Figure 1, left dashed box). This survey was designed to be a detailed assessment of various mental health conditions incorporating questionnaires used in previous studies and common 'gold-standard' research questionnaires (Supplementary File 1). Ethical approval for the study and participant materials was given by the NHS Lothian South East Scotland Research Ethics Committee (REC: 19/SS/0074). After designing the survey, researchers in The University of Edinburgh created a list of random research IDs along with unique questionnaire URLs. These IDs and URLs were sent to the Health Informatics Centre (HIC) in The University of Dundee, who act as Trusted Third Party for all research projects using SHARE. HIC created a look-up table matching research IDs to SHARE IDs, genotype IDs, and with Community Health Index (CHI) numbers that are common across Scottish electronic health records. HIC then sent a list of SHARE IDs to be contacted to the SHARE Scotland team. Email invites (Supplementary File 2) were sent on 5th February 2021 to all SHARE members with a valid email address (N ~ 179,000). At no point were researchers given access to personal identifiers or contact information for SHARE members.

For peer review only

Using their unique URL, participants were able to start the survey and to complete it over multiple sessions if required. Information sheets were sent along with the initial emails and were also presented before any survey questions. This included a warning about the nature of the survey contents and advice about survey length (around 30 minutes to complete, depending on their experience with mental health conditions). To start the survey participants were required to confirm written informed consent, including for linkage of responses to health records, and that they were 16 years of age or older. Start and end dates and completion times were recorded. All responses to the online survey were collected between 5th February 2021 and 27th November 2021. Deidentified questionnaire responses were collated by researchers at The University of Edinburgh. A copy of the survey data was deposited with the SHARE team (and is held by HIC) and can be requested through SHARE's standard data access process.

Data linkage

After the mental health survey, the cohort was enhanced through linkage to genetic data and routinely-collected health records, covering secondary care interactions and prescriptions. Approval for data linkage for individuals across the whole of Scotland was given under the SHARE research register's existing Public Benefit and Privacy Panel approval (Ref: 2122-0165, approved on 21/12/2021). Researchers sent survey responses and study IDs to HIC, who matched responses to the IDs (genetic IDs and Community Health Index (CHI) IDs) needed for data linkage. Matching health record and genetic data was then extracted from data held by HIC. Health record data included patient demographics, secondary care, prescribing and death records collated at a national scale by Public Health Scotland. Individual payload datasets for each data source were deposited in the Safe Haven operated by HIC. The Safe Haven is an NHS-approved Trusted Research Environment, allowing the secure analysis of sensitive data by researchers while minimising risks to privacy. Access to the data is only given to named and accredited researchers. No primary event level data can leave the Safe Haven, and all outputs are checked for statistical disclosure risks by trained HIC staff.

Demographic data

Demographic information is available both from the mental health survey and from routine health records. In the mental health survey, demographic information was collected at the start in order to ensure the best response rate. The survey collected information on age, gender, ethnicity, relationship status and sexual orientation. It also collected health-related demographics, including disability status, height, weight, diet, smoking history and frequency, and amount of sleep. Finally, the survey collected information on socioeconomic circumstances, including amount of education and attainment and employment.

For peer review only

Routinely-collected data provides information on patient demographics (age, sex, etc.) and area-level measures based on the data zone (a unit of geography defined by the Scottish Government to include roughly equal populations) of their residence as recorded on their CHI record and at admission to secondary care [10]. Area measures cover the rurality and remoteness (Urban-Rural Classification) as well as the relative deprivation of their area (The Scottish Index of Multiple Deprivation; SIMD). Notably, the deprivation measure (SIMD) is calculated by combining indicators of income, employment, health, education, geographic access to services, crime and housing deprivation for each of almost 7,000 data zones in Scotland and then ranking them by their overall deprivation [10]. This provides the decile of deprivation for each individual's area, relative to all areas in Scotland, from most to least deprived.

Genetic data

Extracted records also included genetic data derived from a programme of blood sample diversion, which individuals may sign up to upon registering for SHARE. Currently, around 90% of individuals registered with SHARE consent to blood sample diversion. Participants agree to blood samples that are left over from routine care (e.g., when blood tests are ordered by a general practitioner) being sent to a local NHS Scotland Tissue Bank for storage and processing. Samples are processed for genetic data using the Illumina Global Screening Array (https://emea.illumina.com), with the resulting genetic data passed to HIC for retention. Note that this is an ongoing programme, and more genetic data is being added as more samples are diverted and processed. Currently, the blood sample diversion programme operates at the largest hospitals in Scotland, including NHS Tayside, NHS Fife, NHS Grampian, NHS Greater Glasgow and Clyde and NHS Lothian health boards. Rollout of the programme to other health boards is ongoing. Diverted samples are currently available for over 100,00 (around 30%) of consenting SHARE participants. At the time of writing 318 members of the SHARE-MH cohort had genetic data available, though this will rise substantially as more blood samples are diverted and more samples are genotyped. More details on the programme of blood sample diversion and genomic processing can be found in the SHARE cohort profile [9].

Mental health data

The mental health survey included several detailed questionnaires. These covered topics including self-reported diagnoses, depression, suicide, psychosis, anxiety, substance use (alcohol, tobacco, vaping, as well as illegal and non-prescribed substances), adulthood trauma, childhood trauma, serious life events, stress and post-traumatic stress disorder, the impact of mental ill-health, personality and wellbeing. Questionnaires also collected information on episodes of mental ill-health, their length, frequency and seasonality. Standardised questionnaires were included (e.g., Composite International Diagnostic Interview - Short Form

For peer review only - http://bmjopen.bmj.com/site/about/guidelines.xhtml

(CIDI-SF), Mood Disorder Questionnaire (MDQ), Alcohol Use Disorders Identification Test (AUDIT), Childhood Trauma Screener (CTS-5), PTSD Checklist (PCL-S), Work and Social Adjustment Scale (WSAS), Eysenck Personality Questionnaire – Revised (EPQ-R)) alongside questionnaires included in other studies of mental health (UK Biobank mental health online questionnaire; GLAD study online questionnaire). These were chosen to enable multi-cohort studies, greatly increasing the power to investigate risk factors associated with mental illness, and to facilitate cross-cohort comparisons.

Mental health survey responses were enhanced with data from routinely-collected records. These records covered admissions to secondary care settings (Scottish Morbidity Records; SMR) – including outpatient (SMR00; 1996 onwards), general/acute inpatient (SMR01; 1981 onwards), and mental health inpatient (SMR04; 1981 onwards) admissions – as well as community prescribing (Prescribing Information System; 2009 onwards). Records also covered date and cause of death (NHS Death Records; National Records of Scotland Death Records).

Collaboration

Access to the mental health survey data alone can be requested from the University of Edinburgh under Creative Commons Attribution 4.0 International Public License (https://doi.org/10.7488/ds/7491). Access to the survey data for linkage purposes can be obtained through a data access application submitted to the SHARE team (https://www.registerforshare.org/information-for-researchers). Note that linkage of the questionnaire data to health records may be covered under existing approvals obtained by SHARE or may require separate Public Benefit and Privacy Panel approval (https://www.informationgovernance.scot.nhs.uk/pbpphsc/), depending on the nature and scale of the linkage. The role of HIC in the linkage and provision of data within the Safe Haven necessitates a Data Processing Agreement with the research organisation. Under current agreements, data within the Safe Haven can be accessed remotely given appropriate security arrangements. SHARE and HIC operate a cost recovery model to enable research support.

Findings to date

Table 1 describes the SHARE-MH cohort in terms of their demographics, mental health survey response and linkage to health records.

Table 1. Summary of demographic for the SHARE-MH cohort (N = 9,829)

	N Missing (%)	N (%)	Mean (SD)	% Scottish population
Age in years	53 (<1%)		57.16 (14.03)	

For peer review only

16-19 years-old		9 (<1%)		6%
20-34 years-old		793 (8%)		24%
35-49 years-old		1828 (19%)		26%
50-64 years-old		3763 (38%)		24%
65-79 years-old		3141 (32%)		15%
80+ years-old		242 (2%)		5%
Gender	70 (<1%)			
Female		6165 (63%)		52%
Male		3564 (36%)		48%
Other		30 (<1%)		-
Sexual orientation	177 (2%)			
Heterosexual		9178 (93%)		-
Homosexual		246 (3%)		-
Bisexual		204 (2%)		-
Other		24 (<1%)		-
Relationship status	82 (1%)			
Single		1129 (11%)		35%
Relationship		1442 (15%)		-
Married/Civil Partnership		5895 (60%)		46%
Other		1281 (13%)		19%
Highest Educational Qualification	157 (2%)			
None		527 (5%)		27%
Standard Grade		842 (9%)		23%
SVQ Level 1/2		455 (5%)		-
Higher Grade		644 (6%)		14%
SVQ Level 3		391 (4%)		-
SVQ Level 4		1307 (13%)		10%
Degree/Professional		5506 (56%)		26%
Employment status	190 (2%)			
Full-time		3120 (32%)		40%
Part-time		1517 (15%)		13%
Retired		3740 (38%)		15%
Other		405 (4%)		11%
None		706 (7%)		16%
Unemployed		151 (2%)		5%
Ethnicity	241 (2%)			
White		9486 (97%)		96%
Mixed		50 (<1%)		<1%
Asian		41 (<1%)		3%
Other		11 (<1%)		<1%
Smoking status	1263 (13%)			
Current smoker		669 (7%)		-
Past smoker		3196 (32%)		-
Never smoked		4701 (48%)		-
Alcohol status	1162 (12%)			
Current drinker		6901 (70%)		-
Stopped within last 12 months		271 (3%)		-
Stopped over 12 months ago		1117 (11%)		-

For peer review only

Never		378 (4%)		-
Illicit drug status	1287 (13%)			
No		6001 (61%)		-
Yes		2541 (26%)		-
Scottish Index of Multiple Deprivation decile	756 (8%)			
1 – Most deprived		361 (3%)		9%
2		526 (5%)		10%
3		581 (6%)		9%
4		674 (7%)		10%
5		755 (8%)		10%
6		884 (9%)		10%
7		1053 (11%)		10%
8		1288 (13%)		11%
9		1373 (14%)		11%
10 – Least deprived		1578 (16%)		10%

Footnote: Scottish population estimates are taken from Scottish Census 2011 output [11]*, based on a population of N = 4,379,072 individuals aged 16 years and over, with employment status estimates based on a population of N = 3,970,530 aged 16-74 years. The exception is for Scottish Index of Multiple Deprivation deciles, for which population estimates are taken from the 2021 mid-year estimates with N = 4,568,378 individuals aged 16 years and over* [12]

The SHARE-MH cohort broadly resembled the SHARE cohort from which it was selected. An early profile of the wider SHARE register membership indicated that members were predominantly female, middle-aged individuals, though relatively diverse in terms of ethnicity and deprivation [9]. The SHARE-MH cohort also consisted of predominantly female, middle-aged, heterosexual individuals. Indeed, over a third of the Mental Health cohort were retired. Examining SIMD deciles, most participants were from affluent (less deprived) areas of Scotland (1 = 4.0%, 2 = 5.8%, 3 = 6.4%, 4 = 7.4%, 5 = 8.3%, 6 = 9.8%, 7 = 11.6%, 8 = 14.2%, 9 = 15.1%, 10 = 17.4%). Notably, proportionally fewer individuals from deprived areas were represented in the sample, relative to both the SHARE register (1 = 9.3%, 2 = 9.9%, 3 = 9.5%, 4 = 11.1%, 5 = 11.9%, 6 = 9.3%, 7 = 8.4%, 8 = 9.9%, 9 = 10.4%, 10 = 10.3%)(SHARE team, Personal Communication, July 17, 2023) and the wider Scottish population (1 = 9.1%, 2 = 9.6%, 3 = 9.4%, 4 = 9.9%, 5 = 9.9%, 6 = 10.2%, 7 = 10.4%, 8 = 10.7%, 9 = 10.5%, 10 = 10.3%)[12].

Physical and Mental Health

The mental health of participants, as self-reported in the Mental Health survey, are summarised in Table 2. Notably, the majority of the cohort did not report any mental health diagnoses, with over 75% reporting being disability-free. This being said, there was a wide variety of mental health diagnoses being endorsed by those living with mental ill health.

Table 2. Summary of mental health from the Mental Health survey (N = 9,829)

For peer review only

	N Missing (%)	N (%)	Mean (SD)
Mental Health survey responses	0 (0%)		
Complete		8359 (85%)	
Incomplete		1470 (15%)	
Disability status	416 (4%)		
None		7213 (74%)	
Physical only		1197 (12%)	
Mental only		414 (4%)	
Both		589 (6%)	
Sought/received professional help	385 (4%)		
No		4325 (44%)	
Yes		5119 (52%)	
Self-harm	450 (5%)		
No		8062 (82%)	
Yes		1317 (13%)	
Self-reported diagnoses	136 (1%)		
Addiction		207 (2%)	
Anxiety		2641 (27%)	
Attention deficit and hyperactivity disorder		45 (<1%)	
Autism		80 (<1%)	
Bipolar		134 (1%)	
Depression		2845 (29%)	
Eating disorder		250 (3%)	
Obsessive compulsive disorder		149 (2%)	
Personality		103 (1%)	
Phobia		208 (2%)	
Psychosis		40 (<1%)	
Number of diagnoses	136 (1%)		
0		5640 (57%)	
1		2146 (22%)	
2		1386 (14%)	
3		365 (4%)	
4		105 (1%)	
5+		51 (<1%)	

For peer review only

Routinely-collected health records

A brief summary of the SHARE-MH cohort in terms of their routinely-collected health records is presented in Table 3.

Table 3. Number of participants and records identified within routinely-collected health data, as of May 2023

Dataset	N patients (%)	N records	Date of first record

Outpatient (SMR00)	9690 (99%)	332713	April 1996
General Inpatient (SMR01)	8438 (86%)	65787	January 1981
Mental Health Inpatient (SMR04)	231 (2%)	769	December 1994
Community Prescriptions (PIS)	9754 (99%)	1869736	January 2009
Death records	143 (1%)	143	February 2021
Demographic (CHI)	9829 (100%)	9829	July 1948

The most frequent speciality (i.e., discipline) among outpatient records was trauma and orthopaedic surgery (8.7%), followed by dermatology (7.9%) and ophthalmology (7.5%). General psychiatry admissions made up 5.0% of outpatient records. The most frequent operations were unspecified diagnostic testing (4.4%), diagnostic endoscopic examination of the upper gastrointestinal tract (4.0%) and other diagnostic endoscopic examination of the nasopharynx (3.5%).

The most frequent specialities among general inpatient records were general medicine (14.5%), general surgery (12.3%) and trauma and orthopaedic surgery (8.1%). General psychiatry admissions made up less than 1% of general inpatient records. The most frequent diagnostic International Classification of Diseases (ICD)-10 codes included malignant neoplasm of the breast (2.0%), unspecified chest pain (1.4%), and unspecified cataract (1.4%). This broadly reflects the wider SHARE register, with diagnoses of malignancy (around 6%) and heart failure (around 8%) among the most common.

Mental health inpatient admissions consisted of general psychiatry (92.7%), psychiatry of old age (4.0%) and adolescent psychiatry (3.3%) specialities. The most frequent diagnostic ICD-10 codes included an unspecified single episode major depressive disorder (14.9%), emotionally unstable personality disorder (13.5%), and unspecified bipolar disorder (6.2%).

Community prescribing records consisted mainly of prescriptions related to the cardiovascular system (British National Formulary (BNF) chapter 2; 22.7%), central nervous system (BNF chapter 4; 21.0%) and endocrine system (BNF chapter 6; 11.5%). The most frequently dispensed medications included omeprazole (4.0%), levothyroxine (3.6%) and atorvastatin (2.3%). Of the sample, over 5000 individuals (50%) were prescribed an antidepressant according to electronic prescribing records, with antidepressants making up 7.5% of all prescribing records. The rate of antidepressant prescribing was much higher than that of the wider SHARE register (25% of individuals) and the Scottish population (around 18% of individuals)[13], indicating oversampling of antidepressant users. In the present sample, the distribution of specific antidepressants broadly resembled that of the Scottish population [13], with amitriptyline – the most frequent – making up around 2% of all records.

Strengths and limitations

For peer review only

One of the main strengths of the SHARE-MH cohort is the ability to link detailed survey data with genetic and electronic health data. This allows for triangulation of mental health measures over multiple sources of data – both self-reported and clinically-recorded. The mental health survey provides gold-standard measures of mental health, including rarely-available measures of substance use, childhood and adult trauma, and wellbeing. The combination of genetic and routinely-collected health data is particularly unique, and allows researchers to examine the genetic determinants of novel mental health phenotypes, such as treatment exposure and healthcare utilisation. The cohort also provides an opportunity to contribute to mental health consortia and initiatives, such as the Psychiatric Genetics Consortium. It is important to note that the data sources cover more than just mental health, allowing the examination of mental-physical comorbidity and of psychosocial causes and consequences of mental ill health.

The SHARE-MH cohort is limited by the representativeness of the sample. Firstly, participants were recruited to the SHARE register through healthcare settings, and so the SHARE register itself likely captures a relatively ill portion of the Scottish population. Furthermore, while the SHARE register appears to represent the socioeconomic and ethnic composition of the wider Scottish population it over represents middle-aged, female individuals [9], similar to other consented health studies (e.g., the UK Biobank)[14]. Secondly, as SHARE participants were told that the current study focussed on mental health, mental ill health may be overrepresented in this cohort. Indeed, over 40% of the cohort self-reported a mental health diagnosis, larger than recent estimates of the prevalence of mental ill-health in the Scottish population (around 34%) [15]. Similarly, around 50% of the cohort had been prescribed (and dispensed) an antidepressant, much higher than the Scottish population (around 18% of individuals)[13]. Notably, enriching for mental ill health and medication use may benefit particular types of approach, such as psychiatric genetics. Finally, there is some variation in the availability of linked data. Health and genetic data are only available for individuals interacting with the healthcare system. This misses individuals that experience mental ill health but do not seek help (e.g., those with mild symptoms), or those that seek help in other forms (e.g., talking therapies).

Future work

By incorporating survey, genetic and electronic health data there are a variety of research questions that can be addressed, across the fields of depression, anxiety, substance use, childhood and adult trauma and stress. One important area is examining the alignment of treatment experiences – captured by the questionnaire – with actual treatment trajectories – captured by health records. For example, subjective experiences of treatment for depression aligned with depression-related healthcare interactions and antidepressant prescribing can provide fine-grained information on treatment effectiveness. Research-grade measures from the mental health questionnaire are also being used to benchmark recent data-driven mental health phenotypes, such as those from the Health Data Research UK Phenotype Library [16].

For peer review only

Incorporating genetic data can also help to identify subgroups of individuals that respond (or are resistant) to certain treatments. This kind of information can be used to investigate personalised medicine and can help inform sample selection for clinical trials.

There is also ongoing work to increase the amount and breadth of routinely-collected data that can be linked to the cohort. Most notably is the increasing amount of genetic data and number of genotyped participants as sample diversion programmes continue to scale up. There is also work to incorporate novel routinely-collected health datasets, linkable through the same unique CHI number, with national primary care records (i.e., GP data) recently added. Furthermore, recent initiatives such as Research Data Scotland (https://www.researchdata.scot) are helping to increase the variety of available and linkable data beyond health, including census, education and environmental data. These types of data will be especially useful for examining socioeconomic determinants and mediators of mental ill-health as well as the impact of mental ill-health on education and employment. Finally, there is also a drive to make data access easier and more secure by connecting Trusted Research Environments together. Notably, this allows linkage of new sources of data and federation of analyses across multiple cohorts without the need to move data [17,18].

For peer review only

Data sharing statement

De-identified mental health survey data is available from the research team upon request. Linked survey, genetic and routinely-collected health data is available upon approval by the SHARE register team, according to their standard access protocol.

Funding declaration

This work was supported by the Wellcome Trust (Refs: 220857/Z/20/Z,226770/Z/22/Z and 216767/Z/19/Z), by UK Research and Innovation (Ref: MR/W014386/1) and by the European Union Horizon 2020 programme (Ref: 847776).

Contributorship statement

CP, MHI and AMM were responsible for designing the cohort and the mental health survey. CP, MHI, SV, CH and CNAP were involved in distributing the mental health survey to SHARE register participants. MHI and JD collated and cleaned responses to the survey. MHI, JD and EB produced statistical output summarising the cohort. MHI drafted and revised the manuscript. All authors gave feedback on drafts for manuscript revision.

Competing interests statement

CNAP is a co-founder of the SHARE Research Register. Other authors report no competing interests.

Patient and Public Involvement

A stakeholder advisory group, including experts by lived experience and those involved in delivering mental healthcare, were involved when planning the creation of the cohort and consulted regarding the use of electronic health records for mental health research. We also piloted the mental health survey with health service users and used their feedback to further shape the survey and its administration.

Acknowledgements

The authors would like to thank the individuals on the SHARE register for their interest in health research and the participants of the SHARE-MH cohort for providing their time and data. The authors would also like to thank the staff at the Health Informatics Centre Dundee,

For peer review only

particularly Chris Hall, who helped coordinate the indexing of survey, genetic and routinely-collected health data.

For peer review only

References

1 Patel V, Saxena S, Lund C, *et al.* The Lancet Commission on global mental health and sustainable development. *The Lancet*. 2018;392:1553–98.

2 Ford T, Mansfield KL, Markham S, *et al.* The challenges and opportunities of mental health data sharing in the UK. *The Lancet Digital Health*. 2021;3:e333–6.

3 Farber GK, Gage S, Kemmer D, *et al.* Common measures in mental health: a joint initiative by funders and journals. *The Lancet Psychiatry*. 2023;10:465–70.

4 Davies MR, Kalsi G, Armour C, *et al.* The Genetic Links to Anxiety and Depression (GLAD) Study: Online recruitment into the largest recontactable study of depression and anxiety. *Behaviour Research and Therapy*. 2019;123:103503.

5 Davis KAS, Coleman JRI, Adams M, *et al.* Mental health in UK Biobank – development, implementation and results from an online questionnaire completed by 157 366 participants: a reanalysis. *BJPsych open*. 2020;6:e18.

6 Davis KAS, Cullen B, Adams M, *et al.* Indicators of mental disorders in UK Biobank—A comparison of approaches. *Int J Methods Psychiatr Res*. 2019;28. doi: 10.1002/mpr.1796

7 Edgcomb JB, Zima B. Machine Learning, Natural Language Processing, and the Electronic Health Record: Innovations in Mental Health Services Research. *PS*. 2019;70:346–9.

8 John A, McGregor J, Fone D, *et al.* Case-finding for common mental disorders of anxiety and depression in primary care: an external validation of routinely collected data. *BMC Med Inform Decis Mak*. 2016;16:35.

9 McKinstry B, Sullivan FM, Vasishta S, *et al.* Cohort profile: the Scottish Research register SHARE. A register of people interested in research participation linked to NHS data sets. *BMJ Open*. 2017;7:e013351.

10 Scottish Government. *Scottish Index of Multiple Deprivation 2020: Introduction*. 2020. https://www.gov.scot/publications/scottish-index-multiple-deprivation-2020/

11 National Records of Scotland. *Data tables KS102SC, LC1107SC, DC1117SC, LC2101SC, DC5102SC and LC6107SC.* National Records of Scotland 2023. http://www.scotlandscensus.gov.uk/

12 National Records of Scotland. *Population Estimates by Scottish Index of Multiple Deprivation (SIMD)*. Edinburgh: National Records of Scotland 2022. https://www.nrscotland.gov.uk/statistics-and-data/statistics/statistics-by-theme/population/population-estimates/2011-based-special-area-population-estimates/population-estimates-by-simd-2016

13 Public Health Scotland. *Medicines used in Mental Health*. Edinburgh: Public Health Scotland 2021. https://publichealthscotland.scot/media/5941/2021-02-23-prescribingmentalhealth-report.pdf

For peer review only

14 Fry A, Littlejohns TJ, Sudlow C, *et al.* Comparison of Sociodemographic and Health-Related Characteristics of UK Biobank Participants With Those of the General Population. *American Journal of Epidemiology*. 2017;186:1026–34.

15 Scottish Public Health Observatory. Scottish Public Health Observatory Profiles Tool. 2023. https://scotland.shinyapps.io/ScotPHO_profiles_tool/ (accessed 23 March 2023)

16 Chapman M, Mumtaz S, Rasmussen LV, *et al.* Desiderata for the development of next-generation electronic health record phenotype libraries. *GigaScience*. 2021;10:giab059.

17 Gao C, McGilchrist M, Mumtaz S, *et al.* A National Network of Safe Havens: Scottish Perspective. *J Med Internet Res*. 2022;24:e31684.

18 Kavianpour S, Sutherland J, Mansouri-Benssassi E, *et al.* Next-Generation Capabilities in Trusted Research Environments: Interview Study. *J Med Internet Res*. 2022;24:e33720.

For peer review only

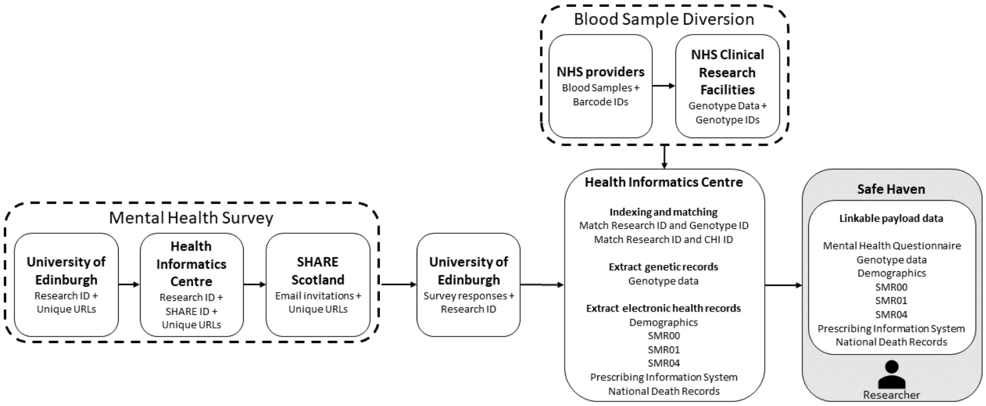

Figure 1. Data flow. Dashed lines indicate processes external to the creation of the SHARE Mental Health cohort. The grey box indicates the Trusted Research Environment operated by the Health Informatics Centre.

855x481mm (38 x 38 DPI)

Block 1: Introduction

Mental health questionnaire for SHARE participants

Questions about your experiences in relation to mental health

Thank you for taking the time to assist us. We are hoping to understand the causes of mental health conditions so that we can try to improve the lives of people who are affected by mental illness. We are interested in your responses whether or not you have ever experienced mental illness yourself.

We are interested in knowing more about the stresses and strains of life and your mental health. Some of the questions are sensitive and may be difficult to answer. Please try to answer as many questions as you can and give the most accurate answers possible. However, if you **cannot** answer a question, you can choose the **"don't know"** or **"prefer not to answer"** option where available. In some instances, it is possible to skip sections and move on.

All responses to questions asked in this questionnaire will be kept strictly confidential, they will only be shared with researchers in a way that means you cannot be identified.

To make it easier for you to participate, you can start the questionnaire and if you leave part way, it will remember where you were when you return to it later. You can see your progress through the survey in the blue bar at the top of the page.

Information on where to find help for the issues covered in this questionnaire is given below and at the end of the survey.

If you feel you need any further help with the issues in this questionnaire, we recommend talking

it through with someone you trust, including your GP. For more information and support, visit The Scottish Association for Mental Health (SAMH).

If you have been upset by remembering sexual violence or any other kind of abuse/crime, please contact support groups for aid or information, such as Scottish Women's Aid (for urgent assistance, give them a call on 0800 027 1234), ManKind (01823 334244 10am-4pm), LGBTdomesticabuse or online Victim Support Scotland.

If you are in distress and need urgent help or advice, please contact someone as soon as possible, such as the **Samaritans on 116 123**

If you would like to take part in the research, please read and select **all** of the statements below.

- ☐ I understand that continuing with this questionnaire is **voluntary** and that I can stop at any point without giving a reason
- ☐ I confirm that I am 16 or older
- ☐ I understand that researchers will combine my answers with an anonymised version of my health records for use in research
- ☐ I consent to take part in this study

Block 2: Basics

What age are you now?

- ○ ________ Current age (years)

Which **gender** do you **identify** with?

- ○ Male
- ○ Female
- ○ Non-binary
- ○ Another gender identity
- ○ Don't know
- ○ Prefer not to answer

Thank you for your interest in taking part in this study. We appreciate your support and willingness to take part in research. You have received this message because you have said that you are **under 16** and we are currently only recruiting participants who are **16 or over**. We would be very interested in hearing from you once you are 16.

Block 3: Demographics/lifestyle

What is your **current marital/relationship status**?

- Single
- Relationship (not living together)
- Relationship (living together)
- Married
- Civil partnership
- Separated
- Divorced
- Widowed
- Other
- Prefer not to say

What is your **sexual orientation?**

- Heterosexual/straight
- Homosexual/gay/lesbian
- Bisexual/bi
- Another sexual orientation
- Don't know
- Prefer not to answer

Do you consider yourself to have a disability?

- Yes, a physical disability
- Yes, a mental health disability
- Yes, both a physical and mental health disability
- No, I don't have a disability
- Don't know
- Prefer not to answer

How old were you when you left High School?

○ [ ] Age (years)

○ I am still in High School

How old were you when you left full-time education?

○ [ ] Age (years)

○ I am still in full-time education

Which of these qualifications do you have?

*Please select **ALL** that apply*

☐ O Grade, Standard Grade, Access 3 Cluster, Intermediate 1 or 3, GCSE, CSE, Senior Certificate or equivalent

☐ SCE Higher Grade, Higher, Advanced Higher, CSYS, A Level, AS Level, Advanced Senior Certificate or equivalent

☐ GSVQ Foundation or Intermediate, SVQ level 1 or 2, SCOTVEC Module, City and Guilds Craft or equivalent

☐ GSVQ Advanced, SVQ level 3, ONC, OND, SCOTVEC National Diploma, City and Guilds Advanced Craft or equivalent

☐ HNC, HND, SVQ level 4 or equivalent

☐ Degree, Postgraduate qualifications, Masters, PhD, SVQ level 5 or equivalent

☐ Professional qualifications (for example, teaching, nursing, accountancy)

☐ Other school qualifications not already mentioned (including foreign qualifications)

☐ Other post-school but pre-Higher Education qualifications not already mentioned (including foreign qualifications)

☐ Other Higher Education qualifications not already mentioned (including foreign qualifications)

☐ No qualifications

What is your **current employment status**?
You can only choose one option so please choose the one that best describes your employment status.

○ In paid employment or self-employed - Full Time

- ◯ In paid employment or self-employed - Part Time
- ◯ Retired (may include volunteering and/or caring for home and/or family)
- ◯ Looking after home and/or family
- ◯ Unable to work because of sickness or disability
- ◯ Unemployed
- ◯ Doing unpaid or voluntary work
- ◯ Full or part-time student
- ◯ None of the above
- ◯ Prefer not to answer

How would you describe your ethnic origin?

- ◯ White
- ◯ Mixed
- ◯ Asian or Asian British
- ◯ Black or Black British
- ◯ Other ethnic group
- ◯ Don't know
- ◯ Prefer not to answer

The next question is about your **height**. Please select your preferred units of measure.

- ◯ Feet/inches
- ◯ Centimetres

What is your **current** height?
(If you are unsure, please give your best estimate)

- ☐ ________ Height (cm)
- ☐ ________ Height (feet)
- ☐ ________ Height (inches)

The next question is about your **weight**. Please select your preferred units of measure.

- ◯ Stones/pounds
- ◯ Kilograms

For peer review only - http://bmjopen.bmj.com/site/about/guidelines.xhtml

What is your **current** weight?
(If you are unsure, please give your best estimate)

☐ ______ Weight (kg)
☐ ______ Weight (stones)
☐ ______ Weight (pounds)

Has a health professional (doctor, nurse, physiotherapist or similar) ever told you that you were overweight?
Please do not include times during pregnancy

○ Yes
○ No
○ Don't know
○ Prefer not to answer

How many **hours per day** do you **sleep** on average?
Please include night time sleep **and** day time naps.

○ ______ Average number of hours per day

Do you have a specific diet?

○ Vegetarian
○ Vegan
○ Pescatarian
○ Other
○ No
○ Prefer not to answer

Block 4: Mental Health History

This section has some questions about your mental health

For peer review only - http://bmjopen.bmj.com/site/about/guidelines.xhtml

In your life, have you suffered from a **period of mental distress** that prevented you from doing your **usual activities**?

- Yes
- No
- Don't know
- Prefer not to answer

In your life, did you **seek or receive help** from a **professional** (medical doctor, psychologist, social worker, counsellor, nurse, clergy or other helping professional) for mental distress, psychological problems or unusual experiences?

- Yes
- No
- Don't know
- Prefer not to answer

Have you ever hurt yourself on purpose in any way (e.g. by taking an overdose of pills, or by cutting yourself)?

- Yes
- No
- Don't know
- Prefer not to answer

Have **YOU** ever been **diagnosed** with one or more of the following illnesses or conditions by a **professional**, even if you don't have it currently?
By professional we mean: any doctor, nurse or person with specialist training (such as a psychologist, psychiatrist etc.).

Please include illnesses or conditions even if you did not need treatment for them or if you did not agree with the diagnosis.

*Please select **ALL** that apply*

- [ ] Addiction or substance use disorder (such as alcohol addiction, drug addiction)

For peer review only - http://bmjopen.bmj.com/site/about/guidelines.xhtml

- [ ] Anxiety (such as generalised anxiety disorder, social anxiety, panic disorder or post-traumatic stress disorder)
- [ ] Autism, Asperger's or autistic spectrum disorder
- [ ] Attention deficit or attention deficit and hyperactivity disorder (ADD/ADHD)
- [ ] Bipolar disorder, mania, hypomania, manic-depression
- [ ] Depression (such as major depressive disorder, seasonal affective disorder, postnatal depression)
- [ ] Eating disorder (such as anorexia, bulimia, binge eating disorder)
- [ ] Obsessive-compulsive disorder (OCD)
- [ ] Personality disorder
- [ ] Phobia (such as social phobia, agoraphobia or another phobia, for example a disabling fear of heights or spiders
- [ ] Schizophrenia or psychosis
- [ ] None of the above
- [ ] Don't know
- [ ] Prefer not to answer

You mentioned that you have been diagnosed with anxiety (such as generalised anxiety disorder, social anxiety, panic disorder or post-traumatic stress disorder). *Please tell us what type(s) of anxiety you have been diagnosed with*

- [ ] Anxiety, nerves or generalised anxiety disorder
- [ ] Panic disorder
- [ ] Post-traumatic stress disorder (PTSD)
- [ ] Social anxiety
- [ ] Don't know
- [ ] Prefer not to answer

You mentioned that you have been diagnosed with depression (such as major depressive disorder, seasonal affective disorder or postnatal depression). *Please tell us what type(s) of depression you have been diagnosed with*

- [ ] Major depressive disorder
- [ ] Postnatal depression (depression during pregnancy for you or your partner or within a year of giving birth)
- [ ] Seasonal Affective Disorder (SAD)
- [ ] Don't know
- [ ] Prefer not to answer

You mentioned that you have had postnatal depression. Were you the person who was pregnant?

- ○ Yes, I was pregnant
- ○ No, my partner was pregnant

You mentioned that you have been diagnosed with an eating disorder.
Please tell us which eating disorder(s) you have been diagnosed with

- ☐ Anorexia nervosa
- ☐ Atypical anorexia nervosa
- ☐ Bulimia nervosa
- ☐ Psychological over-eating or binge eating
- ☐ Don't know
- ☐ Prefer not to answer

You mentioned that you have been diagnosed with a personality disorder.
Please tell us which personality disorder(s) you have been diagnosed with

- ☐ Borderline personality disorder
- ☐ Other personality disorder
- ☐ Don't know
- ☐ Prefer not to answer

You mentioned that you have been diagnosed with a phobia.
Please tell us which phobia(s) you have been diagnosed with

- ☐ Social phobia
- ☐ Agoraphobia
- ☐ Other phobia (such as a disabling fear of heights or spiders)
- ☐ Don't know
- ☐ Prefer not to answer

You mentioned that you have been diagnosed with schizophrenia or another type of psychosis.

For peer review only - http://bmjopen.bmj.com/site/about/guidelines.xhtml

Please tell us which type of psychosis you have been diagnosed with.

- ☐ Schizophrenia
- ☐ Other type of psychosis
- ☐ Don't know
- ☐ Prefer not to answer

Block 5: Mood

The next section asks a few questions about your **mood** and **feelings.**

Have you ever had a time in your life when you have felt sad, blue or depressed for **two weeks or more** in a row?

- ○ Yes
- ○ No
- ○ Prefer not to answer

Have you ever had a time in your life lasting **two weeks or more** when you lost interest in most things like hobbies, work, or activities that usually give you pleasure?

- ○ Yes
- ○ No
- ○ Prefer not to answer

In the next section, we would like to know more about your **depression or low mood**. If you think you may find this distressing, you can **skip** these questions.

- ○ Continue
- ○ Skip

Please think of the **two-week period** in your life when your feelings of depression or loss of interest were worst

How much of the day did these feelings usually last?

- ○ All day long
- ○ Most of the day
- ○ About half of the day
- ○ Less than half of the day

For peer review only - http://bmjopen.bmj.com/site/about/guidelines.xhtml

- Don’t know
- Prefer not to answer

Please think of the **two-week period** in your life when your feelings of depression or loss of interest were worst

Did you feel this way:

- Every day
- Almost every day
- Less often
- Don’t know
- Prefer not to answer

Please think of the **two-week period** in your life when your feelings of depression or loss of interest were worst

Did you feel more **tired** out or **low on energy** than is usual for you?

- Yes
- No
- Don't know
- Prefer not to say

Did you gain or lose weight without trying, or did you stay about the same weight?

- Gained weight
- Lost weight
- Both gained and lost weight during the same episode
- Stayed about the same or was on a diet
- Don't know
- Prefer not to say

Did your weight change by about **10lbs (4kg)** or more?

- Yes
- No
- Don't know
- Prefer not to say

Please think of the **two-week period** in your life when your feelings of depression or loss of interest were worst

Did your **sleep** change?

- Yes
- No
- Don't know
- Prefer not to say

Was that:

Please answer all four questions

	Yes	No
Trouble falling asleep	○	○
Waking too early	○	○
Sleeping too much	○	○
Both sleeping too much and too little during the same episode of depression or loss of interest	○	○

How many **hours per day** did you **sleep on average** during the episode of depression or loss of interest? Please include night time sleep **and** day time naps.

- ______ Hours sleep per day

How many **hours per day** did you used to **sleep** on average when you were **not depressed**? Please include night time sleep **and** day time naps.

- ______ Hours sleep per day

Please think of the **two-week period** in your life when your feelings of depression or loss of interest were worst

Did you experience a **change** in your appetite?

- Increased appetite
- Decreased appetite

For peer review only - http://bmjopen.bmj.com/site/about/guidelines.xhtml

○ Both increased and decreased appetite during the same episode of depression or loss interest

○ No changes in appetite

○ Don't know

○ Prefer not to answer

Please think of the **two-week period** in your life when your feelings of depression or loss of interest were worst

Did your **mood brighten** in response to **positive events**?

○ Yes

○ No

○ Don't know

○ Prefer not to answer

Please think of the **two-week period** in your life when your feelings of depression or loss of interest were worst

Were you **overly sensitive** to comments from other people ?

○ Yes, and this **significantly** impaired your social or work relationships

○ Yes, but this did **not significantly** impair your social or work relationships

○ No

○ Don’t know

○ Prefer not to answer

Please think of the **two-week period** in your life when your feelings of depression or loss of interest were worst

Was your mood **worse:**

☐ In the **morning**

☐ In the **afternoon**

☐ At **night**

☐ My mood did **not** vary

☐ Don't know

☐ Prefer not to answer

Please think of the **two-week period** in your life when your feelings of depression or loss of interest were worst

Did you have a lot more **trouble concentrating than usual?**

- Yes
- No
- Don't know
- Prefer not to answer

Please think of the **two-week period** in your life when your feelings of depression or loss of interest were worst

Did you notice a change in your interest in sex?

- Yes
- No
- Don't know
- Prefer not to answer

Please think of the **two-week period** in your life when your feelings of depression or loss of interest were worst

People sometimes **feel down** on themselves, no good, worthless. Did you feel this way?

- Yes
- No
- Don't know
- Prefer not to answer

Please think of the **two-week period** in your life when your feelings of depression or loss of interest were worst

Did you **think** a lot about **death** – either your own, or someone else's, or death in general?

- Yes
- No
- Don't know
- Prefer not to answer

Did you feel so low you thought a lot about ending your life?

- Yes
- No
- Don't know
- Prefer not to answer

Did you make a suicide plan?

- Yes
- No
- Don't know
- Prefer not to answer

Did you attempt to end your life?

- Yes
- No
- Don't know
- Prefer not to answer

Please think of the **two-week period** in your life when your feelings of depression or loss of interest were worst

Think about your **roles** at the time of this episode, including study/employment, childcare, housework and leisure pursuits. How much did your feelings of depression or loss of interest **interfere** with your life or activities?

- A lot
- Some
- A little
- Not at all
- Don't know
- Prefer not to answer

How many periods of depression or low mood have you had in your life **lasting two or more weeks?**

- One

- Two or three
- More than three
- Don't know
- Prefer not to answer

Please estimate the number of separate episodes of depression or low mood in your life lasting two or more weeks:

- [ ] Estimate of number of depressive episodes

Did any of these episodes occur **during pregnancy (for you or your partner)** or **within the first year of giving birth?** Or has it been suggested you had postnatal depression?

(We are interested in your answer regardless of gender)

- Yes
- No
- Don't know
- Prefer not to answer

You mentioned that you have had postnatal depression. Were you the person who was pregnant?"

- Yes, I was pregnant
- No, my partner was pregnant

About how old were you the **first** time you had a **period of two weeks** like this? (Whether or not you received any help for it.)
An approximate age is fine

- [ ] Approx. age of **first** depressive episode (years)

About how old were you the **last** time you had a **period of two weeks** like this? (Whether or not you received any help for it.)

An approximate age is fine

- [ ] Approx. age of **most recen**t depressive episode (years)

Did you **ever** tell a professional about these problems? *(Medical doctor, psychologist, social worker, counselor, nurse, clergy, or other helping professional)*

- ○ Yes
- ○ No
- ○ Don't know
- ○ Prefer not to say

Did you **ever** try or are you **currently** trying any of the following for these problems? *(Please select **ALL** that apply)*

- ☐ Medications prescribed to you for at least two weeks
- ☐ Unprescribed medication more than once
- ☐ Drugs or alcohol more than once
- ☐ Psychotherapy or other talking therapy more than once
- ☐ None of the above
- ☐ Prefer not to answer

Did you take your medication **as advised**?

- ○ Yes, at least mostly
- ○ No
- ○ Don't know
- ○ Prefer not to answer

Do you think that the prescribed medications helped or are helping you?

- ○ Yes
- ○ No
- ○ Don't know
- ○ Prefer not to answer

You mention that one (or more) medications was helpful or not helpful to you. Some commonly prescribed medications are given below. Please tell us which you found helpful or unhelpful.

For peer review only - http://bmjopen.bmj.com/site/about/guidelines.xhtml

The same medications can have many different names. Each that we mention has been given its common name (e.g. Citalopram) and some common brand names (e.g. celexa, cipramil)

*Please choose an option for **ALL** medications*

	Medication **helpful**	Medication **somewhat helpful**	Medication **not helpful**	Not relevant (medication **no[illegible] prescribed**)
Amitriptyline *Also called: Domical, Elavil, Lentizol, Tryptizol*	○	○	○	○
Citalopram *Also called: Celexa, Cipramil*	○	○	○	○
Fluoxetine *Also called: Prozac, Prozep, Olena, Oxactin*	○	○	○	○
Mirtazapine *Also called: Zispin*	○	○	○	○
Sertraline *Also called: Lustral*	○	○	○	○
Venlafaxine *Also called: Efexor*	○	○	○	○
Other medication not mentioned above	○	○	○	○

You mention that you have tried or are trying psychotherapy or another talking therapy more than once. Please tell us which you have tried and whether you found it helpful or unhelpful.

*Please choose an option for **ALL** therapy types*

	Therapy **helpful**	Therapy **somewhat helpful**	Therapy **not helpful**	Not relevant (not tried this therapy)	Don't know	Prefer not to answe[illegible]
Counselling	○	○	○	○	○	○
Mindfulness	○	○	○	○	○	○

For peer review only - http://bmjopen.bmj.com/site/about/guidelines.xhtml

	Therapy **helpful**	Therapy **somewhat helpful**	Therapy **not helpful**	Not relevant (not tried this therapy)	Don't know	Prefer not to answer
Relationship therapy	○	○	○	○	○	○
Group therapy	○	○	○	○	○	○
Guided self-help	○	○	○	○	○	○
Family therapy	○	○	○	○	○	○
Cognitive behavioural therapy (CBT)	○	○	○	○	○	○
Workshops	○	○	○	○	○	○
Online therapy	○	○	○	○	○	○
Other	○	○	○	○	○	○

We have been asking you about a two-week period but **altogether** over your life, **how long** have your feelings of depression or loss of interest lasted?

If you are unsure, please estimate

- ○ Less than a month
- ○ Between **one** and **three months**
- ○ **Over** three months, but **less** than six months
- ○ **Over** six months, but **less** than 12 months
- ○ Between **one** and **two years**
- ○ **Over** two years
- ○ Don't know
- ○ Prefer not to answer

Was your depression or loss of interest **worse:**

- ☐ In the **Spring**
- ☐ In the **Summer**
- ☐ In the **Autumn**
- ☐ In the **Winter**
- ☐ My mood did **not** vary with the time of year
- ☐ Don't know
- ☐ Prefer not to answer

Block 6: Bipolar illness and psychosis

Now we would like to know about some different symptoms in your **lifetime**. Please answer each question to the best of your ability.

For peer review only - http://bmjopen.bmj.com/site/about/guidelines.xhtml

Has there **ever** been a **period of time** when you were not your usual self and...

(Please respond to each item)

	Yes	No
...you felt so **good** or so **hyper** that other people thought you were not your normal self or you were so hyper that you got into **trouble**?	◯	◯
...you were so **irritable** that you shouted at people or started fights or arguments?	◯	◯
...you felt **much more self-confident** than usual?	◯	◯
...you got much **less** sleep than usual and found you didn't really **miss** it?	◯	◯
…you were much more talkative or spoke **much faster** than usual?	◯	◯
...thoughts raced through your head or you couldn't slow your mind down?	◯	◯
...you were so **easily distracted** by things around you that you had trouble concentrating or staying on track?	◯	◯
...you had **much more energy** than usual?	◯	◯
...you were **much more active** or did many more things than usual?	◯	◯
...you were **much more social** or outgoing than usual, for example, you telephoned friends in the middle of the night?	◯	◯
...you were **much more interested** in sex than usual?	◯	◯
...you did things that were unusual for you or that other people might have thought were **excessive, foolish**, or **risky**?	◯	◯
...spending money got you or your family into trouble?	◯	◯

You ticked **'yes'** to more than one of the previous symptoms – have **several** of these ever happened during the **same period of time**?

- ◯ Yes
- ◯ No

You indicated that you experienced the following. Please select **all** that occurred during the **same period of time**

- ☐ Prefer not to answer
- ☐ Don't know

What is the **longest time** that these "high" or "irritable" periods have lasted?

- ◯ Less than 24 hours

- At least a day, but less than a week
- A week or more
- Don't know
- Prefer not to answer

How much of a **problem** did any of these cause you - like being unable to work; having family, money or legal troubles; getting into arguments or fights?

- No problem
- Minor problem
- Moderate problem
- Serious problem
- Prefer not to answer

Block 7: Anxiety or nerves

This section asks some questions about **anxiety** or **nerves**.

People differ a lot in how much they worry about things. Did you ever have a time when you worried a lot **more than most people** would in your situation?

- Yes
- No
- Don't know
- Prefer not to answer

Have you ever had a period lasting **one month or longer** when most of the time you felt worried, tense, or anxious?

- Yes
- No
- Don't know
- Prefer not to answer

What is the **longest period of time** that this kind of worrying has ever continued?
(If you are not sure of the exact amount of time, please give an estimate)

For peer review only - http://bmjopen.bmj.com/site/about/guidelines.xhtml

☐ Years

☐ Months

Please think of the period in your life when you have felt **worried, tense, anxious,** or **more worried** than most people would in your situation. This could be in the past, or it could be continuing now.
The following questions refer to this period of time.

During that period, was your worry **stronger** than in other people?

- Yes
- No
- Don't know
- Prefer not to answer

Please think of the period in your life when you have felt **worried, tense, anxious,** or **more worried** than most people would in your situation. This could be in the past, or it could be continuing now.
The following questions refer to this period of time.

Did you worry **most days**?

- Yes
- No
- Don't know
- Prefer not to answer

Please think of the period in your life when you have felt **worried, tense, anxious,** or **more worried** than most people would in your situation. This could be in the past, or it could be continuing now.
The following questions refer to this period of time.

Did you usually worry about **one particular thing**, such as your job security or the failing health of a loved one, or **more than one thing**?

- One thing
- More than one thing
- Don't know
- Prefer not to say

Please think of the period in your life when you have felt **worried, tense, anxious,** or **more worried** than most people would in your situation. This could be in the past, or it could be continuing now.
The following questions refer to this period of time.

Did you find it **difficult to stop** worrying?

- Yes
- No
- Don't know
- Prefer not to answer

Please think of the period in your life when you have felt **worried, tense, anxious,** or **more worried** than most people would in your situation. This could be in the past, or it could be continuing now.
The following questions refer to this period of time.

Did you ever have different worries on your mind **at the same time**?

- Yes
- No
- Don't know
- Prefer not to answer

Please think of the period in your life when you have felt **worried, tense, anxious,** or **more worried** than most people would in your situation. This could be in the past, or it could be continuing now.
The following questions refer to this period of time.

How often was your worry so strong that you **couldn't** put it out of your mind no matter how hard you tried?

- Often
- Sometimes
- Rarely
- Never
- Don't know
- Prefer not to answer

Please think of the period in your life when you have felt **worried, tense, anxious,** or **more worried** than most people would in your situation. This could be in the past, or it could be continuing now.
The following questions refer to this period of time.

How often did you find it **difficult to control** your worry?

- Often
- Sometimes
- Rarely
- Never
- Don't know
- Prefer not to answer

When you were **worried or anxious**, were you also:

	Yes	No	Don't know
Restless?	○	○	○
Keyed up or on edge?	○	○	○
Easily tired?	○	○	○
Having difficulty keeping your mind on what you were doing?	○	○	○
More irritable than usual?	○	○	○
Having tense, sore, or aching muscles?	○	○	○
Often having trouble falling or staying asleep?	○	○	○

Did you **ever** tell a **professional** about these problems? *(medical doctor, psychologist, social worker, counsellor, nurse, clergy, or other helping professional)*

- Yes

- No
- Don't know
- Prefer not to answer

Regarding times in your life when you have felt **worried, tense or anxious:** Did you **ever** use the following for the worry or the problems it caused?
Please include any treatments that you have already told us about under 'depression' if they were also for anxiety.
(Please select all that apply)

- Medications prescribed to you for at least two weeks
- Specific anti-anxiety medication prescribed to you for at least one week
- Unprescribed medication more than once
- Drugs or alcohol more than once
- Psychotherapy or other talking therapy more than once
- None of the above
- Prefer not to answer

Did you take your medication **as advised**?

- Yes, at least mostly
- No
- Don't know
- Prefer not to answer

You mention that you have tried or are trying psychotherapy or another talking therapy more than once. Please tell us which you have tried and whether you found it helpful or unhelpful.

	Therapy **helpful**	Therapy **not helpful**	Not relevant (not tried this therapy)	Don't know	Prefer not to answer
Counselling	○	○	○	○	○
Mindfullness	○	○	○	○	○
Relationship therapy	○	○	○	○	○
Group therapy	○	○	○	○	○

For peer review only - http://bmjopen.bmj.com/site/about/guidelines.xhtml

	Therapy **helpful**	Therapy **not helpful**	Not relevant (not tried this therapy)	Don't know	Prefer not to answer
Guided self-help	○	○	○	○	○
Family therapy	○	○	○	○	○
Cognitive behavioural therapy (CBT)	○	○	○	○	○
Workshops	○	○	○	○	○
Online therapy	○	○	○	○	○
Other	○	○	○	○	○

Regarding times in your life when you have felt **worried, tense or anxious:**

Think about your **roles** at the time of this episode, including study/employment, childcare and housework, leisure pursuits. How much did these problems **interfere** with your life or activities?

- ○ A lot
- ○ Some
- ○ A little
- ○ Not at all
- ○ Prefer not to answer

Block 8: Alcohol

In this section, we would like to ask you about alcohol, as we think it may influence mental health.

Your answers will remain confidential so please be honest.

Please state how much you agree with the following statement.

A little bit of alcohol can be good for you

- ○ Very much
- ○ Quite a bit
- ○ Somewhat
- ○ A little bit

○ Not at all
○ Don't know
○ Prefer not to answer

The next questions are about how frequently you drink alcoholic beverages.

Have you ever had an alcoholic drink?

○ Yes, I currently drink alcohol
○ Yes, but I stopped drinking within the last 12 months
○ Yes, but I stopped drinking more than 12 months ago
○ No, I have never had an alcoholic drink

Please think about your use of alcoholic drinks in the **last year**.

How often do you have a drink containing **alcohol?**

○ Never
○ Monthly or less
○ 2 to 4 times a month
○ 2 to 3 times a week
○ 4 or more times a week

Please think about your use of alcohol drinks in the **last year.**

How many units of alcohol do you normally drink in a week?

Alcoholic drink	Approx. units
1 pint of ordinary beer, cider or lager	2
1 pint of extra strength beer /lager	3
1 can of cider	2

For peer review only - http://bmjopen.bmj.com/site/about/guidelines.xhtml

1 litre of cider	5
1 glass of wine (175ml)	2
1 bottle of wine	10
1 bottle of fortified wine (e.g. buckfast or port) (75cl)	11
1 litre of fortified wine (e.g. buckfast or port)	15
1 small glass of sherry	1
1 bottle of sherry (100cl)	15
1 pub measure of spirits (25ml)	1
1 bottle of spirits (75cl)	30
1 bottle of alcopops	2

- ○ [ ] Approximate number of units (per week)

Please think about your use of alcoholic drinks in the **last year**.

How many drinks containing alcohol do you have on a typical day when you are drinking?

- ○ 1 or 2
- ○ 3 or 4
- ○ 5 or 6
- ○ 7, 8 or 9
- ○ 10 or more

Please think about your use of alcoholic drinks in the **last year**.

How often do you have six or more drinks on one occasion?

- ○ Never

For peer review only - http://bmjopen.bmj.com/site/about/guidelines.xhtml

- Less than monthly
- Monthly
- Weekly
- Daily or almost daily

How often during the **last year** have you found that you were **not able to stop** drinking once you had started?

- Never
- Less than monthly
- Monthly
- Weekly
- Daily or almost daily

How often during the **last year** have you failed to do what was normally expected from you because of drinking?

- Never
- Less than monthly
- Monthly
- Weekly
- Daily or almost daily

How often during the **last year** have you **needed** a first drink in the **morning** to get yourself going after a heavy drinking session?

- Never
- Less than monthly
- Monthly
- Weekly
- Daily or almost daily

How often during the **last year** have you had a feeling of **guilt or remorse** after drinking?

- Never
- Less than monthly
- Monthly

For peer review only - http://bmjopen.bmj.com/site/about/guidelines.xhtml

○ Weekly
○ Daily or almost daily

How often during the **last year** have you been **unable** to remember what happened the night before because you had been drinking?

○ Never
○ Less than monthly
○ Monthly
○ Weekly
○ Daily or almost daily

We will now ask you some questions about drinking during your **lifetime**.

Have **you or someone else** been injured as a result of your drinking?

○ No
○ Yes, but not in the last year
○ Yes, during the last year

Has a **relative or friend or a doctor** or another health worker been **concerned** about your drinking or suggested you cut down?

○ No
○ Yes, but not in the last year
○ Yes, during the last year

About how old were you first started drinking alcohol, not counting small tastes or sips?

○ [____] Approximate age (years)

Do you think you have ever been a heavy drinker?

○ Yes, now
○ Yes, now **and** in the past
○ Yes, in the past **but not now**

For peer review only - http://bmjopen.bmj.com/site/about/guidelines.xhtml

- No
- Don't know
- Prefer not to answer

Think about the period in your life when you drank the most. How old were you when that period began?

- [ ] Approximate age (years)

How long did that period last?

- [ ] Approximate length of time (years)

During that period when you drank the most, about how often did you drink?

- Monthly or less
- 2 to 4 times a month
- 2 to 3 times a week
- 4 or more times a week
- Don't know
- Prefer not to answer

During that period when you drank the most, how many **units** of alcohol did you drink on a **typical day** when you are drinking?

**Alcoholic drink**	**Approx. units**
1 pint of ordinary beer, cider or lager	2
1 pint of extra strength beer /lager	3
1 can of cider	2
1 litre of cider	5
1 glass of wine (175ml)	2
1 bottle of wine (75cl)	10
1 bottle of fortified wine (e.g. buckfast or port) (75cl)	11
1 litre of fortified wine (e.g. buckfast or port)	15
1 small glass of sherry	1

For peer review only - http://bmjopen.bmj.com/site/about/guidelines.xhtml

Alcoholic drink	Approx. units
1 bottle of sherry (100cl)	15
1 pub measure of spirits (25ml)	1
1 bottle of spirits (75cl)	30
1 bottle of alcopops	2

- [ ] Approximate number of units (per day)

How much alcohol do you have to drink before you start to feel its effects?

(If you are not sure, your best guess is fine)

- More than other people (drink doesn't seem to affect me as much as other people)
- The same as other people
- Less than other people (I am easily affected by alcohol)
- Don't know
- Prefer not to answer

How bad are your hangovers from drinking alcohol?

- Very bad
- Quite bad
- Not bad at all
- I don't drink enough to get a hangover
- Don't know
- Prefer not to answer

We will now ask you some questions about your reasons for drinking alcohol

Please state how often the following statement is true.

I feel more comfortable around others when I drink

(please think about now and in the past)

- Always or almost always

- Often
- Sometimes
- Rarely
- Never
- Don't know
- Prefer not to answer

Please state how often the following statement is true.

I sleep better after I drink

(please think about now and in the past)

- Always or almost always
- Often
- Sometimes
- Rarely
- Never
- Don't know
- Prefer not to answer

Please state how often the following statement is true.

I drink because I am nervous or anxious

(please think about now and in the past)

- Always or almost always
- Often
- Sometimes
- Rarely
- Never
- Don't know
- Prefer not to answer

Please state how often the following statement is true.

For peer review only - http://bmjopen.bmj.com/site/about/guidelines.xhtml

I drink because I am depressed

(please think about now and in the past)

- Always or almost always
- Often
- Sometimes
- Rarely
- Never
- Don't know
- Prefer not to answer

Please state how often the following statement is true.

I drink because I am in physical pain

(please think about now and in the past)

- Always or almost always
- Often
- Sometimes
- Rarely
- Never
- Don't know
- Prefer not to answer

Please state how often the following statement is true.

I drink because I am angry, annoyed or bad-tempered

(please think about now and in the past)

- Always or almost always
- Often
- Sometimes
- Rarely

- Never
- Don't know
- Prefer not to answer

Please state how often the following statement is true.

I drink to escape my memories or thoughts

(please think about now and in the past)

- Always or almost always
- Often
- Sometimes
- Rarely
- Never
- Don't know
- Prefer not to answer

Please state how often the following statement is true.

I drink because I feel tense or stressed

(please think about now and in the past)

- Always or almost always
- Often
- Sometimes
- Rarely
- Never
- Don't know
- Prefer not to answer

Block 9: Smoking

In this section, we will ask you about smoking

Please select the box that best describes you

- I smoke now
- I used to smoke
- I have never smoked
- Prefer not to answer

How many years has it been since you stopped smoking?
(An approximate answer is fine)

- [ ] Years since stopped smoking

Approximately how many cigarettes do you or did you smoke each day?

- [ ] Number of cigarettes per day

How many years have you / did you smoke?

- [ ] Years smoking

How many hours per week are you **exposed** to other people's tobacco smoke (*passive smoking*)?

- [ ] Hours per week

Have you ever tried an e-cigarette or vaping device?

- Yes
- No
- Don't know
- Prefer not to answer

How often do you currently use an e-cigarette or vaping device?

- Daily or almost daily

- Less than daily, but at least once a week
- Less than weekly, but at least once a month
- Less than monthly
- Not at all
- Don't know
- Prefer not to answer

Have you ever used and e-cigarette or vaping device daily for a month or more?

- Yes
- No
- Don't know
- Prefer not to answer

Block 10: Illegal substances

In this section, we would like to ask you about illegal substances, as we think they may influence mental health.

Your answers will remain confidential so please be honest.

Have you **ever** taken any **drugs** other than those prescribed for you for medical reasons?

For example ***cannabis, cocaine, speed, amphetamines, ecstasy, LSD, heroin*** *or* ***methadone*** *or substances previously called* ***"legal" highs*** *such as* ***spice****.*

We do **not** mean **prescription drugs** and will ask about these later.

- Yes
- No
- Prefer not to answer

You mention that you have taken drugs in your lifetime. Please tell us how often you took them when you were using them most.

For peer review only - http://bmjopen.bmj.com/site/about/guidelines.xhtml

*Please choose an option for **ALL** groups of drugs*

	Never	Once or twice only	Monthly or less	2 to 4 times a month	2 to 3 times a week	4 or more times a week	Prefer not to answer
Heroin or methadone	○	○	○	○	○	○	○
Cocaine	○	○	○	○	○	○	○
Cannabis or marijuana	○	○	○	○	○	○	○
Speed, amphetamines or ecstasy	○	○	○	○	○	○	○
Spice or other substance previously called a "legal" high	○	○	○	○	○	○	○
Magic mushrooms or LSD	○	○	○	○	○	○	○
Ketamine	○	○	○	○	○	○	○
Other illegal drug	○	○	○	○	○	○	○

And how long did you take heroin or methadone for?
If you are still taking them, please give the time so far.
If you are unsure an estimate is fine.

- ○ [ ] Years
- ○ Less than one year
- ○ [ ] I am still taking heroin or methadone. Years so far
- ○ Prefer not to answer

And how long did you take cocaine for?
If you are still taking it, please give the time so far.
If you are unsure an estimate is fine.

- ○ [ ] Years
- ○ Less than one year
- ○ [ ] I am still taking cocaine. Years so far
- ○ Prefer not to answer

And how long did you take cannabis or marijuana for?
If you are still taking them, please give the time so far.
If you are unsure an estimate is fine.

For peer review only - http://bmjopen.bmj.com/site/about/guidelines.xhtml

- [ ] Years
- Less than one year
- [ ] I am still taking cannabis or marijuana. Years so far
- Prefer not to answer

And how long did you take speed, amphetamines or ecstasy for?
If you are still taking them, please give the time so far.
If you are unsure an estimate is fine.

- [ ] Years
- Less than one year
- I am still taking speed, amphetamines or ecstasy. Years so far [ ]
- Prefer not to answer

And how long did you take magic mushrooms or LSD for?
If you are still taking them, please give the time so far.
If you are unsure an estimate is fine.

- [ ] Years
- Less than one year
- [ ] I am still taking magic mushrooms or LSD. Years so far
- Prefer not to answer

And how long did you take ketamine for?
If you are still taking them, please give the time so far.
If you are unsure an estimate is fine.

- [ ] Years
- Less than one year
- [ ] I am still taking ketamine. Years so far
- Prefer not to answer

And how long did you take spice or other substance previously called a "legal" high for?

If you are still taking them, please give the time so far.

If you are unsure an estimate is fine.

- ◯ [ ] Years
- ◯ Less than one year
- ◯ I am still taking spice or other substance previously called a "legal" high. Years so far [ ]
- ◯ Prefer not to answer

Have you **ever** taken **prescription drugs** that were **not prescribed for you?**

*For example: **valium, diazepam, temazepam, other 'benzos'** or **opioids** such as **fentanyl, tramadol, buprenorphine, oxycodone**.*

- ◯ Yes
- ◯ No
- ◯ Prefer not to answer

You mention that you have taken prescription drugs (not prescribed for you) in your lifetime. Please tell us how often you took them when you were using them most.

*Please choose an option for **ALL** groups of drugs*

	Never	Once or twice only	Monthly or less	2 to 4 times a month	2 to 3 times a week	4 or more times a week	Prefer not to answer
Valium, diazepam, temazepam or other *"benzo"*	◯	◯	◯	◯	◯	◯	◯
Fentanyl, tramadol, buprenorphine, oxycodone or other opiod (excluding heroin and methdaone)	◯	◯	◯	◯	◯	◯	◯
Other prescription drug that was not prescribed for you	◯	◯	◯	◯	◯	◯	◯

And how long did you take valium, diazepam, temazepam or another "benzo" for?

If you are still taking them, please give the time so far.

If you are unsure an estimate is fine.

- ◯ ______ Years
- ◯ Less than one year
- ◯ I am still taking valium, diazepam, temazepam or another "benzo". Years so far ______
- ◯ Prefer not to answer

And how long did you take an opioid such as fentanyl, tramadol, buprenorphine or oxycodone for?

If you are still taking them, please give the time so far.

If you are unsure an estimate is fine.

- ◯ ______ Years
- ◯ Less than one year
- ◯ I am still taking an opioid such as fentanyl, tramadol, buprenorphine or oxycodone. ______ Years so far
- ◯ Prefer not to answer

Block 11: Trauma

This section asks about your childhood and the stresses and strains of adult life. Some people may find these questions distressing. If you would prefer not to answer these questions, please use a **skip** button below.

If you are distressed by remembering past crime/abuse, please visit [Victim Support Scotland]

- ◯ Continue
- ◯ Skip childhood questions
- ◯ Skip adult questions
- ◯ Skip childhood and adult questions

When I was growing up...

	Never true	Rarely true	Sometimes true	Often true	Very often true	Prefer not to answer
I felt loved	○	○	○	○	○	○
People in my family hit me so hard that it left me with bruises or marks	○	○	○	○	○	○
I felt that someone in my family hated me	○	○	○	○	○	○
Someone molested me (sexually)	○	○	○	○	○	○
There was someone to take me to the doctor if I needed it	○	○	○	○	○	○

If you feel distressed from remembering past crime/abuse, please visit Victim Support Scotland. *If you have been upset by remembering domestic or sexual violence, there is information available at Scottish Women's Aid (for urgent assistance, give them a call on 0800 027 1234),* ManKind (01823 334244 10am-4pm) *or* LGBTdomesticabuse

Since I was sixteen...

	Never	Yes, but not in the last 12 months	Yes, within the last 12 months	Prefer not to answer
I have been in a confiding relationship	○	○	○	○
A partner or ex-partner deliberately hit me or used violence in any other way	○	○	○	○
A partner or ex-partner repeatedly belittled me to the extent that I felt worthless	○	○	○	○
A partner or ex-partner sexually interfered with me, or forced me to have sex against my wishes	○	○	○	○
I have struggled to find the money to pay my rent/ mortgage payment	○	○	○	○

If you feel distressed from remembering past crime/abuse, please visit Victim Support Scotland. *If you have been upset by remembering domestic or sexual violence, there*

For peer review only - http://bmjopen.bmj.com/site/about/guidelines.xhtml

is information available at [Scottish Women's Aid](#) (for urgent assistance, give them a call on 0800 027 1234), [ManKind](#) (01823 334244 10am-4pm) *or* [LGBTdomesticabuse](#)

You said that since you were sixteen, you have been in a confiding relationship. Please indicate how often this was true.

- Rarely true
- Sometimes true
- Often true
- Very often true
- Prefer not to answer

You said that since you were sixteen, a partner or ex-partner deliberately hit you or used violence in another way. Please indicate how often this was true.

- Rarely true
- Sometimes true
- Often true
- Very often true
- Prefer not to answer

If you feel distressed from remembering past crime/abuse, please visit [Victim Support Scotland](#). *If you have been upset by remembering domestic or sexual violence, there is information available at [Scottish Women's Aid](#) (for urgent assistance, give them a call on 0800 027 1234),* [ManKind](#) (01823 334244 10am-4pm) *or* [LGBTdomesticabuse](#)

You said that since you were sixteen, a partner or ex-partner repeatedly belittled you to the extent that you felt worthless. Please indicate how often this was true.

- Rarely true
- Sometimes true
- Often true
- Very often true
- Prefer not to answer

If you feel distressed from remembering past crime/abuse, please visit Victim Support Scotland. *If you have been upset by remembering domestic or sexual violence, there is information available at* *Scottish Women's Aid* *(for urgent assistance, give them a call on 0800 027 1234),* ManKind (01823 334244 10am-4pm) *or* LGBTdomesticabuse

You said that since you were sixteen, a partner or ex-partner sexually interfered with you, or forced you to have sex against your wishes. Please indicate how often this was true.

- Rarely true
- Sometimes true
- Often true
- Very often true
- Prefer not to answer

If you feel distressed from remembering past crime/abuse, please visit Victim Support Scotland. *If you have been upset by remembering domestic or sexual violence, there is information available at* *Scottish Women's Aid* *(for urgent assistance, give them a call on 0800 027 1234),* ManKind (01823 334244 10am-4pm) *or* LGBTdomesticabuse

You said that since you were sixteen, you have struggled to find the money to pay your rent/mortgage payment. Please indicate how often this was true

- Rarely true
- Sometimes true
- Often true
- Very often true
- Prefer not to answer

In your life, have you...

	Never	Yes, but not in the last 12 months	Yes, within the last 12 months	Prefer not to answer
Been a victim of a sexual assault, whether by a stranger or someone you knew	O	O	O	O

	Never	Yes, but not in the last 12 months	Yes, within the last 12 months	Prefer not to answer
Been attacked, mugged, robbed, or been the victim of a physically violent crime	○	○	○	○
Been in a serious accident that you believed to be life-threatening at the time	○	○	○	○
Witnessed a sudden violent death (e.g. murder, suicide, aftermath of an accident)	○	○	○	○
Been diagnosed with a life-threatening illness	○	○	○	○
Been involved in combat or exposed to a war-zone (either in the military or as a civilian)	○	○	○	○

If you feel distressed from remembering past crime/abuse, please visit Victim Support Scotland. *If you have been upset by remembering domestic or sexual violence, there is information available at Scottish Women's Aid (for urgent assistance, give them a call on 0800 027 1234),* ManKind (01823 334244 10am-4pm) *or* LGBTdomesticabuse

Next, is a list of **problems and complaints** that people sometimes have in response to extremely stressful experiences. Please indicate how much you have been bothered by that problem in the **past month**:

	Not at all	A little bit	Moderately	Quite a bit	Extremely
Repeated, disturbing memories, thoughts, or images of a stressful experience?	○	○	○	○	○
Feeling very upset when something reminded you of a stressful experience?	○	○	○	○	○
Avoiding activities or situations because they reminded you of a stressful situation?	○	○	○	○	○
Feeling distant or cut off from other people?	○	○	○	○	○
Feeling irritable or having angry outbursts?	○	○	○	○	○
Difficulty concentrating?	○	○	○	○	○

If you feel distressed from remembering past crime/abuse, please visit Victim Support Scotland. *If you have been upset by remembering domestic or sexual violence, there is information available at Scottish Women's Aid (for urgent assistance, give them a call on 0800 027 1234),* ManKind (01823 334244 10am-4pm) *or* LGBTdomesticabuse

Block 12: Impact

Questions about your work and social life

People's problems sometimes **affect** their ability to do certain day-to-day tasks in their lives.

To rate your problems look at each section and determine on the scale provided **how much** your problem **impairs/impaired your ability** to carry out the activity.

Please complete this section thinking about problems you have been asked about like **anxiety, depression and sleep problems.**

If you have had multiple issues, please think of the issue that has impacted you **most severely**.

Because of my problem my ability to **work** is/was impaired. If you are retired or choose not to have a job for reasons unrelated to your problem, please tick NA *(not applicable).*

- Not at all
- Slightly
- Moderately
- Markedly
- Very severely, I cannot/could not work
- NA
- Prefer not to answer

Because of my problem my **home management** *(Cleaning, tidying, shopping, cooking, looking after home/children, paying bills)* is/was impaired.

- Not at all
- Slightly
- Moderately
- Markedly
- Very severely
- Prefer not to answer

Because of my problem my **social leisure activities** *(with other people eg. parties, bars, clubs, outings, visits, dating, home entertainment)* are impaired.

- Not at all
- Slightly
- Moderately
- Markedly
- Very severely
- Prefer not to answer

Because of my problem my **private leisure activities** *(done alone, such as reading, gardening, collecting, sewing, walking alone)* are impaired.

- Not at all
- Slightly
- Moderately
- Markedly
- Very severely
- Prefer not to answer

Because of my problem **my ability to form and maintain close relationships** with others, including those I live with, is impaired.

- Not at all
- Slightly
- Moderately
- Markedly
- Very severely
- Prefer not to answer

Block 13: Eyseneck

The following 12 questions are about your feelings

Does your mood often go up and down?

- Yes

No

Do you ever feel 'just miserable' for no reason?

Yes

No

Are you an irritable person?

Yes

No

Are your feelings easily hurt?

Yes

No

Do you often feel 'fed-up'?

Yes

No

Would you call yourself a nervous person?

Yes

No

Are you a worrier?

Yes

No

Would you call yourself tense or 'highly strung'?

Yes

For peer review only - http://bmjopen.bmj.com/site/about/guidelines.xhtml

No

Do you worry too long after an embarrassing experience?

Yes

No

Do you suffer from nerves?

Yes

No

Do you often feel lonely?

Yes

No

Are you often troubled about feelings of guilt?

Yes

No

Block 14: Subjective wellbeing

This is the **last** section of the survey.
In this section we would like to know how you **feel** about things **in general.**

In general, how **happy** are you?

Extremely happy

Very happy

Moderately happy

Moderately unhappy

Very unhappy

Extremely unhappy

For peer review only - http://bmjopen.bmj.com/site/about/guidelines.xhtml

- Don't know
- Prefer not to answer

In general, how happy are you with your **health?**

- Extremely happy
- Very happy
- Moderately happy
- Moderately unhappy
- Very unhappy
- Extremely unhappy
- Don't know
- Prefer not to answer

To what extent do you feel your life to be **meaningful**?

- An extreme amount
- Very much
- A moderate amount
- A little
- Not at all
- Don't know
- Prefer not to answer

Please submit your survey by pressing the next arrow below

If you feel you need any further help with the issues in this questionnaire, we recommend talking it through with someone you trust, including your GP. For more information and support, visit The Scottish Association for Mental Health (SAMH).

If you have been upset by remembering sexual violence or any other kind of abuse/crime, please contact support groups for aid or information, such as Scottish Women's Aid (for urgent assistance, give them a call on 0800 027 1234), ManKind (01823 334244 10am-4pm), LGBTdomesticabuse or online Victim Support Scotland.

If you are in distress and need urgent help or advice, please contact someone as soon as possible, such as the **Samaritans on 116 123**

For peer review only - http://bmjopen.bmj.com/site/about/guidelines.xhtml

Powered by Qualtrics

Contact email and patient information leaflet: Version 03 14/11/19

SHARE Online survey – A mental health survey for Scotland

MHDSS – A mental health survey for Scotland

Dear

You very kindly joined SHARE, the Scottish Health Research Register, as you were interested in hearing about opportunities to help with medical research. We are writing to let you know about a research opportunity that has arisen and that might appeal to you. Please see information from the study team below:

Participation in this research study involves completing **an online survey** about mental health.

You can start the survey by clicking the link below:

https://unique.url.per.participant

You will be able to complete the survey on a home computer, tablet or smart-phone.

Further details about the study are provided below.

For peer review only

Contact email and patient information leaflet: Version 03 14/11/19

Mental Health Data Science Scotland

Participant Information Leaflet

What am I being invited to do?

You are invited to take part in an online survey about mental health. The survey answers will be used for health research and there will be no direct compensation for individuals taking part in the survey.

What will the survey involve?

If you decide to take part, you will be asked a series of questions about yourself and any history of mental ill-health you may have had. We will then ask for some details about things that may have an impact on mental health, such as drinking alcohol, taking illegal substances and difficult experiences in your life. Finally, there will be some questions about your feelings. The questions will not include asking for your email or any other identifying information.

Please be aware that the survey concerns mental health and some questions may be distressing for some participants. **Participation is voluntary** and you can leave the survey at any time by closing your internet browser. We also give participants the option to skip some sections should they wish.

How long will the survey take? Can I come back later?

On average, the whole survey takes approximately **15 minutes to complete**. It may take less time if you have limited experience of mental health problems and will probably take **20-25 minutes** if you have more experience with low moods. We do not think it will take anyone more than **one hour**. If you are not able to complete the survey in one go you can **return to it later** by clicking on the link you have been sent (please note you will need to make sure you are using the same computer/device to access the survey). The link you have been sent to the survey is unique so, if more than one person in your household has been sent this email, please only use the link that was sent to you.

What is the research about?

The aim of the research is to better understand why people develop mental illnesses and how that links with their physical health. We will do that by collecting information from participants and linking this with their health records. The information collected will **not** be added to your health record, it will be stored separately and will be only be used for research purposes. It will be **strictly confidential** and only shared with

researchers in a way that means you cannot be identified. In the future, your responses may also be used by other researchers to select you for further studies of interest but your participation would be completely voluntary.

Who is conducting the research?

This survey is being carried out by researchers at the University of Edinburgh and it is funded by a grant from the Medical Research Council (MRC).

What about data security?

The questionnaire deals with sensitive information so every care is being taken with regard to data security. The software we are using to collect answers is called Qualtrics. The company behind this software works with the NHS, companies and universities and has no access to any of the answers that participants give. All answers will be collected, encrypted and stored electronically on an ISO 27001 certified, secure server. They will then be securely transferred to the University of Edinburgh and from there to an NHS data Safe Haven which also uses ISO 27001 certified servers. The information held by the University of Edinburgh will contain no identifying information. Within the Safe Haven, a separate encrypted file will be used to link the responses with anonymised electronic health records. Researchers must then do all of their work within the Safe Haven and cannot remove information about individuals. Results can only be removed about groups of people. If you would like to know more about how we approach data security and how the National Safe Havens work, please visit our website here https://mhdss.ac.uk/what-about-security-and-privacy

Do I have to take part?

No, you do not have to take part in this survey. We will ask you to consent at the beginning of the questionnaire and, if you agree, this would indicate a willingness for your data to be used by the research team. Should you wish to withdraw your data, you can email <<MHDSS email>> and give us the **email address that was used to contact you**. You do not need a reason and we do not need your name or any details of your responses. If your questionnaire answers have already been used in analyses, they cannot be removed because they will have been anonymised before analysis but we will contact the Safe Haven team and they will delete your answers so that they cannot be used again.

Where can I find more information?

You can find out more about the team behind this study and our wider work via our website Mental Health Data Science Scotland or you can email enquiries to the team working with this questionnaire at <<MHDSS email>>.

If you wish to contact someone who has knowledge of the study, but is not part of the team working on this questionnaire, please contact <<External contact>> by emailing <<External's email address>> or writing to <<External's postal address>>.

What should I do if I want to make a complaint?

Any concerns should be directed in the first instance to the team at Edinburgh University using <<MHDSS email>>. If you have concerns which you believe have not been addressed then complaints can be made to the Head of the Division of Psychiatry at the University of Edinburgh, <<HoD details>>. Email: <<HoD email address>> or write to <<HoD postal address>>.

For peer review only

STROBE Statement—Checklist of items that should be included in reports of ***cohort studies***

	Item No	Recommendation	Page No
**Title and abstract**	1	(*a*) Indicate the study's design with a commonly used term in the title or the abstract	1
		(*b*) Provide in the abstract an informative and balanced summary of what was done and what was found	2
**Introduction**			
Background/rationale	2	Explain the scientific background and rationale for the investigation being reported	4
Objectives	3	State specific objectives, including any prespecified hypotheses	4
**Methods**			
Study design	4	Present key elements of study design early in the paper	5
Setting	5	Describe the setting, locations, and relevant dates, including periods of recruitment, exposure, follow-up, and data collection	5
Participants	6	(*a*) Give the eligibility criteria, and the sources and methods of selection of participants. Describe methods of follow-up	5
		(*b*) For matched studies, give matching criteria and number of exposed and unexposed	NA
Variables	7	Clearly define all outcomes, exposures, predictors, potential confounders, and effect modifiers. Give diagnostic criteria, if applicable	6-8
Data sources/ measurement	8*	For each variable of interest, give sources of data and details of methods of assessment (measurement). Describe comparability of assessment methods if there is more than one group	6-8
Bias	9	Describe any efforts to address potential sources of bias	5
Study size	10	Explain how the study size was arrived at	5
Quantitative variables	11	Explain how quantitative variables were handled in the analyses. If applicable, describe which groupings were chosen and why	NA
Statistical methods	12	(*a*) Describe all statistical methods, including those used to control for confounding	NA
		(*b*) Describe any methods used to examine subgroups and interactions	NA
		(*c*) Explain how missing data were addressed	NA
		(*d*) If applicable, explain how loss to follow-up was addressed	NA
		(*e*) Describe any sensitivity analyses	NA
**Results**			
Participants	13*	(a) Report numbers of individuals at each stage of study—eg numbers potentially eligible, examined for eligibility, confirmed eligible, included in the study, completing follow-up, and analysed	5-6; 10-11
		(b) Give reasons for non-participation at each stage	5-6; 10-11
		(c) Consider use of a flow diagram	5
Descriptive data	14*	(a) Give characteristics of study participants (eg demographic, clinical, social) and information on exposures and potential confounders	8-12
		(b) Indicate number of participants with missing data for each variable of interest	8-9
		(c) Summarise follow-up time (eg, average and total amount)	11
Outcome data	15*	Report numbers of outcome events or summary measures over time	8-12

For peer review only - http://bmjopen.bmj.com/site/about/guidelines.xhtml

Main results	16	(*a*) Give unadjusted estimates and, if applicable, confounder-adjusted estimates and their precision (eg, 95% confidence interval). Make clear which confounders were adjusted for and why they were included	NA
		(*b*) Report category boundaries when continuous variables were categorized	NA
		(*c*) If relevant, consider translating estimates of relative risk into absolute risk for a meaningful time period	NA
Other analyses	17	Report other analyses done—eg analyses of subgroups and interactions, and sensitivity analyses	NA
**Discussion**			
Key results	18	Summarise key results with reference to study objectives	12
Limitations	19	Discuss limitations of the study, taking into account sources of potential bias or imprecision. Discuss both direction and magnitude of any potential bias	12-13
Interpretation	20	Give a cautious overall interpretation of results considering objectives, limitations, multiplicity of analyses, results from similar studies, and other relevant evidence	12-13
Generalisability	21	Discuss the generalisability (external validity) of the study results	12-13
**Other information**			
Funding	22	Give the source of funding and the role of the funders for the present study and, if applicable, for the original study on which the present article is based	15

*Give information separately for exposed and unexposed groups.

Note: An Explanation and Elaboration article discusses each checklist item and gives methodological background and published examples of transparent reporting. The STROBE checklist is best used in conjunction with this article (freely available on the Web sites of PLoS Medicine at http://www.plosmedicine.org/, Annals of Internal Medicine at http://www.annals.org/, and Epidemiology at http://www.epidem.com/). Information on the STROBE Initiative is available at http://www.strobe-statement.org.

For peer review only
